# Decoding Adsorption Mechanisms in Biomass-Derived Adsorbents: From Experimental Evidence and Molecular Simulation to Explainable and Transferable Prediction

**DOI:** 10.3390/molecules31214018

**Published:** 2026-11-07

**Authors:** Ximena Jaramillo-Fierro, Julián Hidalgo, Eduardo Valarezo

**Affiliations:** 1 Departamento de Química y Producción, Facultad de Ciencias Exactas y Naturales, Universidad Técnica Particular de Loja, San Cayetano Alto, Loja 110107, Ecuador; bevalarezo@utpl.edu.ec; 2 Carrera de Ingeniería Química, Facultad de Ciencias Exactas y Naturales, Universidad Técnica Particular de Loja, San Cayetano Alto, Loja 110107, Ecuador

**Keywords:** biochar, surface complexation, density functional theory, molecular dynamics, SHAP, external validation

## Abstract

Biomass-derived adsorbents are increasingly used for aqueous pollutant removal, yet adsorption mechanisms are often inferred from model fitting or isolated characterization rather than convergent molecular evidence. This scoping evidence review integrates experimental surface chemistry, density functional theory (DFT), molecular dynamics (MD), and machine learning (ML) to examine the transition from mechanistic interpretation to transferable prediction. Using a PRISMA-ScR-informed systematic evidence mapping approach, a relevance-prioritized analytical corpus of 125 primary studies was constructed, comprising 70 mechanistic and 55 predictive studies. Evidence charting was closed after the mechanistic and predictive categories stabilized during relevance-prioritized synthesis. Electrostatic interactions, hydrogen bonding, surface complexation/coordination, π interactions, and pore filling were the most recurrent mechanisms and frequently coexisted. Static adsorbate–surface DFT predominated, whereas solvent/speciation-aware calculations and dynamic interfacial models remained comparatively limited. The synthesis shows that kinetic or isotherm fitting cannot uniquely establish molecular mechanisms and that ML feature importance or SHapley Additive exPlanations (SHAP) attribution should not be interpreted as causal evidence. Predictive transferability was more convincingly demonstrated through grouped, external, or experimental validation than through random data partitioning. Based on these findings, we propose the Molecular Mechanistic Evidence Framework (MMEF), integrating molecular depth, evidence convergence, environmental realism, interpretive discipline, and predictive transferability to guide adsorption research toward molecularly supported, experimentally testable, and prospectively predictive adsorbent design.

## 1. Introduction

The adsorption of aqueous pollutants by biomass-derived materials has evolved from a performance-oriented field toward an increasingly molecular and data-driven research domain. Biochar, hydrochar, biomass-derived activated carbons, lignocellulosic adsorbents, and their modified derivatives combine heterogeneous pore structures, surface functional groups, mineral phases, and tunable interfacial chemistry, enabling interactions with chemically diverse contaminants. Consequently, current research increasingly seeks not only to quantify pollutant removal but also to determine why adsorption occurs, which surface sites and interactions dominate, how solution chemistry modifies these processes, and whether mechanistic knowledge can ultimately support prediction and rational adsorbent design.

A wide range of mechanisms has consequently been proposed, including pore filling and confinement, electrostatic attraction, hydrogen bonding, π-mediated interactions, hydrophobic association, surface complexation and coordination, ion exchange and bridging, precipitation, and adsorption-coupled redox processes [1,2,3,4]. However, the central challenge is no longer the absence of proposed mechanisms, but the unequal depth, independence, and environmental relevance of the evidence used to support them. Kinetic and equilibrium models remain useful for describing adsorption behavior, but goodness of fit does not uniquely identify a molecular mechanism. In particular, translating pseudo-second-order fitting directly into chemisorption or Langmuir fitting into proof of monolayer adsorption constitutes an evidentiary overreach when independent chemical evidence is absent [5,6,7]. Surface characterization through Fourier-transform infrared spectroscopy (FTIR), X-ray photoelectron spectroscopy (XPS), Raman spectroscopy, zeta potential, pH at the point of zero charge (pH_pzc_), elemental analysis, and related techniques provides additional evidence, but mechanistic confidence ultimately depends on whether independent observations converge on the same chemical interpretation.

Molecular simulation provides a deeper level of mechanistic resolution. Density functional theory (DFT) can identify preferential adsorption configurations and sites, quantify interaction energies, and examine charge redistribution, orbital interactions, and noncovalent interactions, whereas molecular dynamics (MD) can additionally describe diffusion, pore entry, hydration, competition, and dynamic interfacial organization. Nevertheless, “DFT included” is not itself a meaningful measure of mechanistic strength. Molecular representations range from isolated-pollutant descriptors to explicit adsorbate–surface calculations, solvent- and speciation-aware DFT, and explicit water or experimentally constrained atomistic MD. Recent studies illustrate this progression from solvated electronic-structure calculations [8] toward explicit water MD [9] and experimentally informed atomistic models [10,11]. Thus, the mechanistic value of molecular simulation depends primarily on model–system correspondence, including representation of surface heterogeneity, solvation, pollutant speciation, competing species, and pore environments.

In parallel, machine learning (ML) is increasingly being used to predict adsorption performance, optimize biochar synthesis, and identify influential material, molecular, and operational descriptors. Previous reviews have established the growing role of ML in biochar research [12,13], while recent studies increasingly combine molecular descriptors, fingerprints, DFT-derived information, and explainable ML [14,15]. However, predictive accuracy, interpretability, mechanistic validity, and transferability are distinct outcomes. Random train/test partitions primarily evaluate interpolation within represented data, whereas grouped validation, genuinely independent external datasets, and prospective experiments assess distinct forms of generalization beyond the model development domain [16]. Likewise, SHAP values, partial-dependence plots, and feature importance identify model associations but do not independently establish molecular causation. In this sense, “high SHAP importance = mechanism” and “pseudo-second-order (PSO) fit = chemisorption” represent analogous inferential risks when association is promoted to mechanism without independent corroboration.

Some emerging studies are beginning to bridge these traditionally separated evidence domains by combining experimental characterization, molecular simulation, and interpretable prediction [8,15,17,18]. Particularly informative are emerging closed-loop approaches in which prediction generates a molecular hypothesis, DFT evaluates the proposed interaction, and targeted experiments test the resulting prediction [19]. Yet existing reviews largely address adsorption mechanisms, molecular simulation, biochar engineering, or ML as related but incompletely integrated domains. The unresolved question is therefore what evidence is required for an adsorption mechanism to progress from being proposed to independently supported, molecularly resolved, environmentally tested, and ultimately predictively transferable.

Accordingly, this scoping evidence review systematically maps the molecular and predictive evidence supporting adsorption mechanisms of aqueous pollutants on biomass-derived adsorbents. Five interconnected research questions (RQs) are addressed: RQ1, which mechanisms are proposed and what evidence supports them; RQ2, where independent evidence strengthens mechanistic interpretation and where model fitting is overinterpreted; RQ3, how DFT and MD corroborate, refine, or challenge experimentally inferred mechanisms; RQ4, which surface, physicochemical, and molecular descriptors show potential transferability; and RQ5, under what conditions experimental characterization, molecular simulation, and explainable ML can enable generalizable prediction and rational adsorbent design. Accordingly, the synthesis evaluates the proposition that mechanistic confidence is strengthened when experimental, surface-chemical, and molecular evidence converge, rather than when mechanisms are inferred primarily from adsorption model fitting and examines whether integrating molecular and surface descriptors with explainable ML can support the transition from mechanistic interpretation toward transferable prediction.

From this synthesis, we introduce the Molecular Mechanistic Evidence Framework (MMEF), an emergent multidimensional framework organized around five partially independent dimensions: molecular depth, evidence convergence, environmental realism, interpretive discipline, and predictive transferability. The MMEF is not a numerical quality score or a fixed hierarchy; rather, it provides a common language for evaluating the complementary evidence underlying mechanistic confidence. The central proposition of this review is therefore that the transition from proposed to predictive adsorption mechanisms requires not more evidence of the same type, but convergence among complementary forms of evidence. This perspective provides a route toward biomass-derived adsorbents whose performance can not only be measured and explained retrospectively but ultimately predicted and rationally designed.

## 2. Recurring Adsorption Mechanisms and Their Evidentiary Basis (RQ1)

### 2.1. Multimechanistic Adsorption Landscape

The aqueous pollutants examined in this review can be broadly distinguished as organic and inorganic contaminants. Organic contaminants include dyes, pharmaceuticals and personal care products (PPCPs), antibiotics, pesticides, and per- and polyfluoroalkyl substances (PFAS). Microplastics represent a distinct class of particulate polymeric contaminants that may also influence adsorption through their surfaces and associated co-contaminants. Inorganic contaminants include heavy metals and other potentially toxic elements, metalloids, oxyanions, and nutrient species such as phosphate and ammonium. This distinction provides a chemical basis for interpreting the adsorption mechanisms discussed below, while recognizing that pollutant speciation, surface chemistry, and solution conditions can produce overlapping interaction pathways.

Across the 70 retained mechanistic studies, adsorption was rarely attributed to a single interaction. Electrostatic interactions were reported in 34/70 studies (48.6%), hydrogen bonding in 31/70 (44.3%), surface complexation or coordination in 29/70 (41.4%), π interactions in 28/70 (40.0%), pore filling or confinement in 24/70 (34.3%), and van der Waals or other noncovalent interactions in 22/70 (31.4%). Hydrophobic interactions (8/70; 11.4%), redox or transformation processes (8/70; 11.4%), ion exchange or bridging (7/70; 10.0%), and precipitation (4/70; 5.7%) were less frequent but became decisive in specific systems. Because mechanisms commonly co-occurred, these frequencies overlap and should be interpreted as descriptive patterns within the retained mechanistic corpus rather than as mutually exclusive categories or prevalence estimates for the broader adsorption literature. To complement the overall distribution of adsorption mechanisms, Supplementary Figure S1 presents a heat map of reported associations between pollutant classes and adsorption interactions. The analysis includes 65 of the 70 retained mechanistic studies with normalized mechanism labels available in the evidence-charting matrix.

For organic contaminants, several studies converged on combinations of pore filling, π interactions, hydrogen bonding, electrostatics, hydrophobic interactions, and dispersion forces, but the relative importance of these interactions depended on pore architecture, heteroatom chemistry, pollutant charge state, and molecular size [1,3,20,21,22,23,24,25,26]. Accordingly, recurring interaction labels should be treated as conditional hypotheses rather than universal explanations for organic-pollutant uptake.

Antibiotic and pharmaceutical systems particularly illustrate this conditionality. Across tetracycline, ciprofloxacin, norfloxacin, tylosin, metronidazole, paracetamol, diclofenac, and related compounds, adsorption was associated with different combinations of aromatic interactions, hydrogen bonding, electrostatics, coordination, pore matching, and heteroatom-specific binding [27,28,29,30,31,32,33,34]. These studies collectively show that the same pollutant class can access different dominant interactions when the adsorbent surface, pore environment, or solution chemistry changes.

Per- and polyfluoroalkyl substances (PFAS) adsorption provides another clear example of mechanism dependence on molecular and interfacial context. Several studies implicate a combination of dispersion or hydrophobic association, electrostatic effects, pore accessibility, surface functionality, and chain-length-dependent contact with carbonaceous domains [15,35,36,37,38]. Mixed adsorption–photodegradation work on PFOS is useful as a methodological boundary case because molecular calculations directed primarily toward catalytic/electronic behavior should not automatically be counted as direct adsorption evidence [39].

For metals, metalloids, phosphate, ammonium, and other inorganic species, the mechanistic landscape extends beyond noncovalent adsorption to coordination, ligand exchange, ion exchange or bridging, precipitation, and redox transformation. This is evident across Cu(II), Pb(II), Cr(VI), Sb, As species, phosphate, ammonium, and multimetal systems [2,17,40,41,42,43,44,45,46,47,48]. In these systems, total removal should not be equated automatically with adsorption because reduction or precipitation can contribute substantially to the observed disappearance from solution.

Multisolute, competitive ion, and complex water studies further demonstrate that mechanisms inferred from single-solute experiments are not automatically transferable. Cu-mediated antibiotic co-adsorption, microplastic-associated secondary sorption domains, competitive anions, natural organic matter, and coexisting contaminants can alter site accessibility, screening, bridging, speciation, and the balance among adsorption and transformation pathways [4,49,50,51,52,53,54]. Environmental complexity is therefore itself mechanistic evidence rather than merely an application-stage test.

Table 1 provides a rapid cross-mechanism comparison of frequency, diagnostic evidence, interpretive warnings, and representative studies.

**Figure 1:**
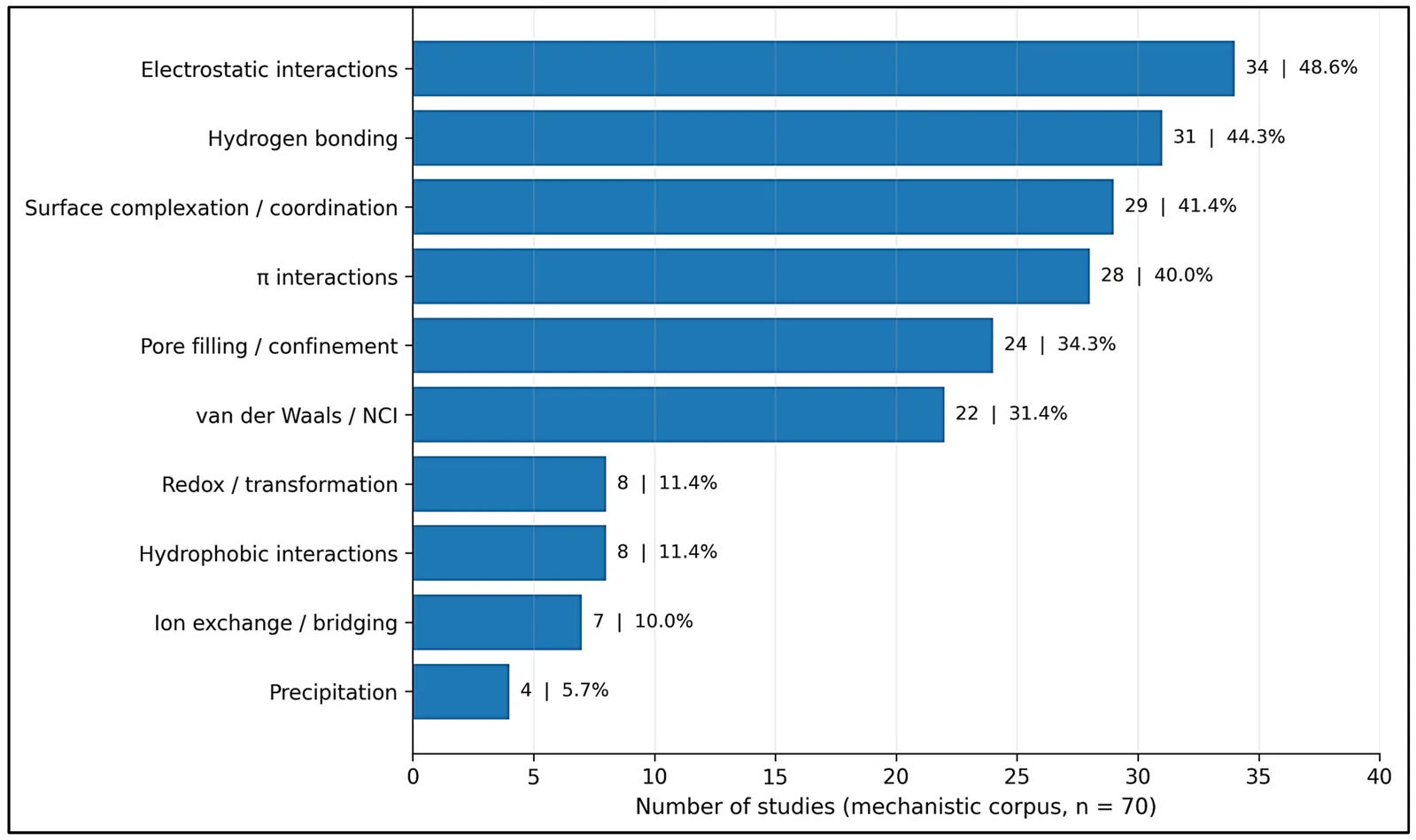
Recurring adsorption mechanisms identified in the retained mechanistic corpus (*n* = 70). Mechanisms are non-exclusive; percentages therefore do not sum to 100%.

The relative frequency of the recurring, non-exclusive adsorption mechanisms identified across the mechanistic corpus is visualized in Figure 1.

### 2.2. RQ1 Synthesis

RQ1 shows that biomass-derived adsorbents share a recurring interaction vocabulary, but not a universal mechanism. The mechanistic meaning of electrostatics, hydrogen bonding, π interactions, pore filling, coordination, ion exchange, redox, or precipitation depends on pollutant speciation, surface chemistry, pore architecture, and aqueous composition. The most informative studies therefore discriminate dominant from secondary interactions, test how the mechanism changes with chemical context, and separate adsorption from parallel removal pathways. This conclusion leads directly to RQ2: when several mechanistic narratives can be attached to the same macroscopic adsorption response, how strong is the evidence used to support them?

## 3. From Model Fitting to Mechanistic Evidence: What Kinetics and Isotherms Can—and Cannot—Establish (RQ2)

### 3.1. Model Fitting Is Descriptive Evidence, Not Molecular Observation

Kinetic and equilibrium models remain useful for comparing adsorption rates, capacities, and empirical response patterns. The evidentiary problem begins when model identity is translated directly into molecular proof—for example, when pseudo-second-order (PSO) fitting is treated as proof of chemisorption or when Langmuir/Freundlich preference is treated as definitive evidence of mono- versus multilayer adsorption or homogeneous versus heterogeneous binding. Critical analyses of adsorption modeling have emphasized that different mathematical forms can fit the same data, and that fitted model preference does not uniquely identify a microscopic pathway [5,6,7]. In this review, kinetic and isotherm fits are therefore treated as macroscopic evidence that may be consistent with a mechanism but cannot independently prove it.

### 3.2. Manual Audit of Fit-to-Mechanism Overinterpretation

The claim-level audit confirmed that this inferential shortcut remains common. Within the retained mechanistic corpus (*n* = 70), 32 studies contained at least one confirmed local instance in which kinetic or equilibrium model fitting was used to infer chemical versus physical adsorption or a comparable microscopic surface interpretation. Twenty-five studies contained confirmed kinetic-fit-to-mechanism claims and 26 contained confirmed isotherm/equilibrium-fit-to-mechanism claims; these categories overlap. Candidate claims were identified through a rule-based screen and the evidence-charting notes and were verified at full text; two candidates were false positives and were excluded, and one could not be verified because its full text was unavailable and was not counted. These counts are therefore conservative. These results identify local evidentiary overreach, not necessarily an incorrect overall mechanism.

Several of these papers nevertheless contain strong independent evidence supporting a similar mechanistic conclusion. For Cu(II), Pb(II), and Cu/fulvic-acid systems, for example, spectroscopic changes, charge measurements, DFT energetics, projected density of states (PDOS), or charge-transfer analyses provide a stronger mechanistic basis than the kinetic or isotherm label itself [17,56,63]. The appropriate criticism is therefore local: the mechanism may be plausible or even well supported, while one specific inferential step remains unjustified.

The same audit also shows why model fitting and molecular evidence should be evaluated separately. Bouzid et al. [64], Tabassam et al. [65], and Peng et al. [66] combine adsorption model interpretation with additional molecular or surface evidence. These cases support evaluating each inferential step on its own rather than treating agreement with a kinetic or equilibrium model as molecular proof.

### 3.3. Evidence Independence Is More Informative than Evidence Quantity

The audit supports an evidence-independence principle. Kinetic and isotherm models describe macroscopic response; pH_pzc_, zeta potential, and pH perturbation provide charge-state context; FTIR, Raman, and post-adsorption XPS probe changes in chemical environments; functional group blocking or controlled modification can test the involvement of candidate sites; and DFT/MD can evaluate energetic, geometric, and dynamic plausibility. Agreement among methods generated by different physical principles is more informative than several fits extracted from the same adsorption dataset.

### 3.4. Worked Examples: Discriminating Between Competing Adsorption Mechanisms

Three representative studies illustrate how converging evidence can distinguish between competing adsorption mechanisms and clarify the limitations of the mechanistic interpretation.

First, π–π interactions versus hydrogen bonding. Fang et al. [1] investigated the adsorption of bisphenol S on oriental plane tree (*Platanus orientalis*) biochars prepared at different pyrolysis temperatures. Both π–π interactions and hydrogen bonding were chemically plausible. However, DFT calculations, FTIR and Raman spectroscopy, and adsorption thermodynamics collectively supported a greater contribution from π–π interactions in the high-temperature biochar. This interpretation was consistent with the increased adsorption capacity following pyrolysis at 600 °C. This example illustrates how molecular calculations and independent characterization can discriminate between competing noncovalent interactions.

Second, local binding affinity versus material-scale elimination pathways. Liu et al. [42] investigated phosphate removal using chitosan hydrogel beads and magnesite-modified algal biochar. DFT calculations identified stronger phosphate binding at the amino and hydroxyl groups (−44.6 and −37.7 kcal mol^−1^, respectively) than at the modeled Mg coordination site (−18.6 kcal mol^−1^). However, experimental characterization supported additional contributions from surface precipitation, coordination, and electrostatic attraction. Therefore, the most favorable binding site in a simplified molecular model does not necessarily represent the dominant removal pathway in the heterogeneous material. Distinguishing local interaction energies from material-scale removal processes prevents adsorption, coordination, and precipitation from being incorrectly treated as interchangeable mechanisms.

Third, ternary surface complexation versus competitive inhibition. Guo et al. [63] examined the adsorption of Cu(II) on Mn-modified algal biochar in the presence of fulvic acid. At a relatively low concentration of fulvic acid (10 mg L^−1^), Cu(II) uptake increased, consistent with a higher surface charge and the formation of a ternary complex. At a higher concentration (20 mg L^−1^), adsorption decreased because competitive interactions and the blocking of surface sites became more significant. DFT calculations supported the anchoring of Cu(II) at Mn–O and carboxyl sites, followed by fulvic acid-mediated bridging. This example demonstrates that seemingly conflicting mechanistic explanations can describe different experimental regimes rather than mutually exclusive pathways.

Taken together, these examples establish three complementary principles: competing molecular interactions should be evaluated using independent evidence; favorable local binding energies should not be equated with the overall removal mechanism; and mechanistic contributions can vary with solution composition.

### 3.5. RQ2 Synthesis

RQ2 therefore identifies a problem of inferential discipline rather than a reason to abandon adsorption modeling. Kinetic and isotherm equations remain valuable descriptive tools, but molecular claims should be anchored in independent chemical, surface, perturbational, or molecular evidence. This principle becomes decisive for RQ3 because DFT and MD can either provide genuinely independent evidence or merely decorate a mechanism that was already assumed from macroscopic model fitting.

## 4. Molecular-Level Validation: What DFT and Molecular Dynamics Actually Add (RQ3)

### 4.1. “DFT Included” Is Not a Homogeneous Evidence Category

The molecular evidence audit revealed a broad continuum rather than a binary distinction between studies with and without computation. Of the 70 retained mechanistic studies, 3 (4.3%) contained no direct molecular adsorption simulation (M0), 6 (8.6%) relied mainly on indirect molecular descriptors or isolated-molecule evidence (M1), 1 (1.4%) used statistical-physics/non-atomistic molecular interpretation (M1b), 39 (55.7%) used explicit adsorbate–surface static DFT (M2), 12 (17.1%) incorporated solvent- or speciation-aware surface DFT (M3), 7 (10.0%) used dynamic interfacial MD (M4), and 2 (2.9%) used experimentally constrained explicit water MD (M5). These are descriptive archetypes of model–system correspondence, not quality scores.

Explicit adsorbate–surface DFT provides a more direct molecular bridge by comparing binding sites, configurations, adsorption energies, charge redistribution, electronic coupling, or noncovalent interactions. This approach is represented across organic contaminants, metals, oxyanions, and heteroatom- or metal-modified carbons [24,34,55,58,59]. The gain in mechanistic specificity, however, depends on whether the modeled surface and adsorbate state correspond credibly to the experimental system.

Additional retained studies extend this molecular evidence across different biomass-derived surfaces and pollutant systems [67,68]. Their contribution is considered here in terms of the molecular representation and its correspondence with the experimental system, rather than the mere presence of a computational calculation.

### 4.2. Solvation, Speciation, and Surface Realism Determine the Meaning of Molecular Evidence

To distinguish molecular evidence according to model–system correspondence rather than by the mere presence of computation, the molecular representations identified in the corpus are organized into the archetypes summarized in Table 2.

Direct adsorbate–surface molecular models were present in 60/70 studies (85.7%), whereas 37/70 (52.9%) were classified as solvent-aware. Solvent awareness was coded as a cross-cutting attribute independently of the mutually exclusive M0–M5 archetype and therefore should not be inferred by summing the M3–M5 categories. Studies were classified as solvent-aware when their molecular analysis explicitly incorporated or evaluated solvent-related effects relevant to adsorption, even when the study’s primary molecular evidence archetype was assigned on the basis of a different defining feature. This difference is chemically important because aqueous adsorption reflects competition among adsorbate–surface affinity, hydration, surface solvation, ionic screening, and protonation or speciation. Several studies explicitly incorporated water or solution-state effects and thereby narrowed the gap between optimized molecular structures and the experimental interface [8,29,38,51,61,75].

Speciation-aware modeling is especially important for ionizable organics, PFAS, ammonium, metals, and oxyanions, because the dominant charge state can change the preferred interaction and even the apparent adsorption pathway. Studies that combine pH-dependent experiments, competitive ions, explicit charge states, or redox partitioning therefore provide more chemically informative molecular evidence than calculations performed for a single arbitrarily chosen species [2,42,45,50,53].

### 4.3. MD Adds Dynamic and Competitive Evidence Unavailable from Static Minima

Only 9/70 studies (12.9%) used dynamic MD evidence, making interfacial dynamics one of the clearest gaps in the corpus. MD can address questions that static DFT cannot resolve directly: pore entry, residence near functional groups, solvent reorganization, ion bridging, competitive occupation, diffusion, and time-dependent organization inside heterogeneous pores. Several studies used MD to examine these processes for pharmaceuticals, pesticides, multisolute greywater contaminants, and porous carbon architectures [9,25,52,57].

Dynamic models with greater model–system correspondence move beyond generic carbon slabs toward experimentally informed representations. Wood et al. constrained biochar structures using more than 300 experimental descriptor datasets and simulated 2,4-D adsorption with explicit water and Na^+^ [10]. Sochacki et al. likewise used experimentally informed biochar models and long explicit water multisolute simulations relevant to greywater chemistry [11]. These studies illustrate that the principal advance is not simply greater computational cost but stronger correspondence between the simulated interface and the material being interpreted.

### 4.4. Molecular Sophistication and Evidentiary Completeness Are Orthogonal

Theory-only benchmarks can provide rigorous comparative energetics without constituting experimental validation, while experimentally rich studies can still rely on idealized molecular models. Sulfur-functionalized defective-carbon calculations and aqueous Fe-site PFAS calculations, for example, are valuable for isolating molecular interactions but should not be narrated as direct validation of a particular experimental biochar unless the model–material correspondence is demonstrated [59,60]. Conversely, studies involving crude-oil/metal mixtures, real-water matrices, or complex composites may possess strong environmental or experimental realism while the molecular model captures only part of the interfacial complexity [31,76,77].

### 4.5. RQ3 Synthesis

**Figure 2:**
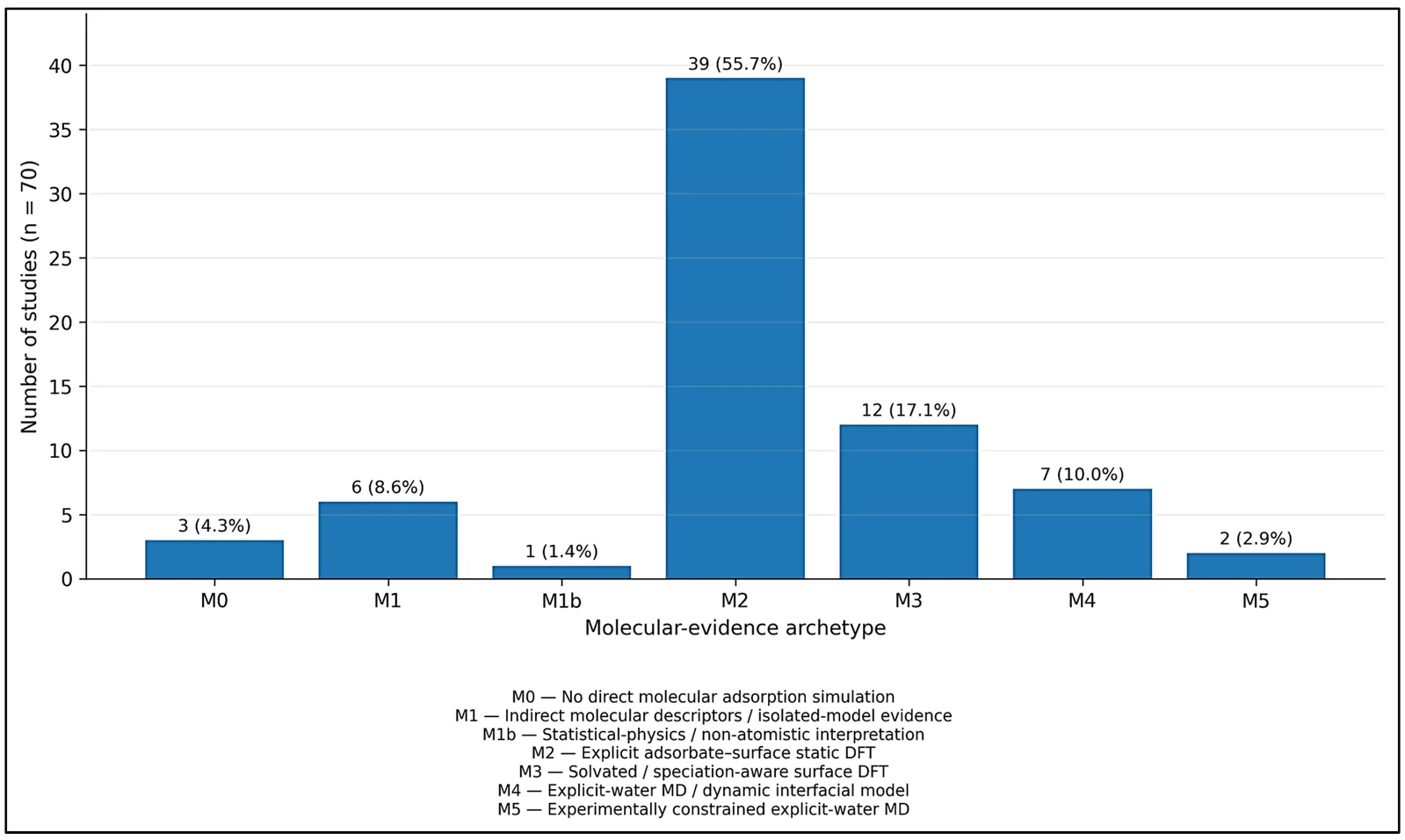
Molecular evidence depth in the retained mechanistic corpus (*n* = 70). Archetypes describe model–system correspondence and are not a unidimensional quality score.

The distribution of the retained mechanistic studies across these molecular evidence archetypes is shown in Figure 2.

RQ3 changes the operative question from “Was DFT or MD performed?” to “How faithfully does the molecular model represent the adsorbate, surface, solvent, speciation, pore environment, and competitive chemistry relevant to the experimental claim?” The resulting continuum—indirect molecular descriptors → explicit surface DFT → solvent/speciation-aware DFT → dynamic MD → experimentally constrained atomistic models—captures increasing model–system correspondence but should not be collapsed into a single quality score. Molecular depth should therefore be interpreted alongside experimental corroboration, environmental realism, and the scope of the mechanistic claims.

The complementary evidentiary roles and inferential limits of the principal experimental, molecular, and data-driven approaches discussed in RQ2 and RQ3 are compared in Table 3.

## 5. Transferable Molecular and Surface Descriptors: From Recurrent Predictors to Chemical Generalization

### 5.1. Transferability Is a Property of Relationships, Not of Isolated Feature Rankings

RQ4 asks whether descriptors identified in individual adsorption studies retain mechanistic or predictive meaning across different biomass-derived adsorbents, pollutant classes, and aqueous conditions. The integrated evidence does not support a single universal variable. Instead, recurrent predictors organize into chemically interpretable families: textural and pore descriptors; surface composition and functionality; charge and acid–base descriptors; pollutant physicochemical and structural descriptors; quantum/electronic descriptors; and operational or environmental variables. The important distinction is that recurrence is not equivalent to transferability. A descriptor may repeatedly receive high feature importance because it is common to many datasets, while its direction or mechanistic meaning may change with speciation, pore exclusion, surface charge, co-solutes, or dataset construction.

Several predictive studies converge on this multi-scale structure. Across heavy metal, nutrient, pharmaceutical, antibiotic, dye, pesticide, and mixed-contaminant models, specific surface area, pore metrics, elemental composition, pH-related variables, adsorbent dose, concentration, and surface chemistry repeatedly appear among influential predictors [81,82,83,84,85,86,87,88,89,90,91,92]. Collectively, these studies indicate that transferable description requires both adsorbent-side and environment-side information rather than reliance on any single property.

The same descriptor architecture is represented in additional predictive studies that combine textural, surface-chemical, molecular, preparation, and operational variables in different proportions [93,94,95,96,97,98]. Their recurrence supports the use of descriptor families, while the transferability of individual variables still depends on validation outside the conditions represented during model development.

### 5.2. Descriptor Families and Their Mechanistic Interpretation

Across the predictive corpus, useful representations consistently combined adsorbent-side, adsorbate-side, and environmental information. Textural descriptors capture accessibility and confinement; surface composition, functional groups, pH_pzc_ and zeta potential represent interaction potential; molecular size, hydrophobicity, pK_a_, aromaticity and fingerprints describe pollutant chemical space; quantum descriptors interrogate interaction energetics; and operational variables define the conditions under which these interactions are expressed. Their recurrence across studies does not imply that any single variable is universally transferable.

The descriptor families identified across the predictive corpus, together with their mechanistic interpretation, transferability requirements, and principal cautions, are summarized in Table 4.

Representative predictive studies support this multi-scale architecture across heavy metals, pharmaceuticals, antibiotics, dyes, nutrients, PFAS, and mixed contaminants [14,16,81,84,85,86,88,99]. Molecular and surface descriptors become most persuasive when statistical importance is independently tested by spectroscopy, perturbation experiments, or molecular calculations [8,15,17,19].

### 5.3. Descriptor Relevance Is Not Descriptor Transferability

A cross-study conclusion is that feature importance within a dataset does not, by itself, establish descriptor transferability. Assessing whether predictive relationships persist across chemical and material domains requires validation beyond the conditions represented during model development. Relevant approaches include compound-grouped or material-grouped evaluation, independent external datasets, and prospective experiments. Several retained studies report external or experimental validation [14,80,82,84,100,101,102,103,104].

By contrast, the mobile-organic-compound study demonstrates why chemical-space validation is more demanding: performance deteriorated under compound-grouped evaluation relative to random partitioning, indicating that interpolation can overestimate generalization to unseen chemicals [16]. Kubar et al. provide a complementary counterexample in which test performance was substantially weaker than training performance, emphasizing the risk of overfitting in small or heterogeneous literature-derived datasets [105].

Accordingly, a transferable adsorption descriptor is best defined here as a chemically interpretable variable whose predictive direction persists across materials, pollutants, or environmental domains and is supported by validation beyond the distribution from which it was learned.

This broader pattern is also reflected in predictive studies spanning multi-compound organic adsorption, sulfonamide and amoxicillin removal, hydrochar and modified-biochar systems, heavy metal adsorption, pharmaceuticals and personal care products (PPCPs) removal, and 2,4-D uptake, in which textural, compositional, molecular, and operating descriptors are combined in different proportions according to the chemical domain being modeled [69,106,107,108,109,110,111,112,113,114,115,116]. Their collective value lies less in identifying one universal predictor than in showing that useful models require descriptor sets matched to adsorbent chemistry, pollutant identity, and operating domain.

### 5.4. RQ4 Synthesis

RQ4 therefore does not identify a universal descriptor; it identifies a transferable descriptor architecture. Textural variables describe accessibility and confinement, surface chemistry and charge describe interaction potential, pollutant descriptors describe molecular compatibility and speciation, quantum descriptors interrogate interaction energetics, and environmental variables determine which interactions are expressed under actual aqueous conditions. The strongest predictive representations combine these domains rather than privileging a single scale.

## 6. From Explainable Prediction to Mechanistically Informed Adsorbent Design

### 6.1. Predictive Accuracy, Interpretability, Mechanistic Plausibility, and Transferability Are Distinct Outcomes

The predictive corpus shows rapid growth in tree-based ensembles, neural networks, support-vector methods, Gaussian-process models, stacking, transformers, and related algorithms. High R2 values are common, but predictive accuracy alone does not establish mechanistic understanding or generalization. Numerous studies use train/test splitting, K-fold validation, or both, while a smaller subset includes external, grouped, or prospective validation [81,85,86,87,88,89,117,118,119,120,121].

This pattern is also evident in retained studies using combinations of tree-based methods, neural networks, support-vector approaches, and model-attribution tools [122,123,124,125,126,127,128]. These studies are therefore used as evidence of current predictive practice, not as evidence that model attribution alone identifies an adsorption mechanism. A study-level audit of the 55 retained predictive studies (Table S1) revealed that internal evaluation procedures predominated. Train/test partitioning was explicitly recorded in 47 studies (85.5%), and cross-validation in 34 studies (61.8%). External validation was reported in 13 studies (23.6%), while 18 studies (32.7%) reported some form of experimental validation. These categories were non-exclusive, as individual studies could employ multiple evaluation procedures. Importantly, the reported designation of external validation did not necessarily establish independence from the model development domain, and experimental confirmation was not automatically classified as prospective validation. Three studies (5.5%) required additional extraction to clarify their validation procedures. Compound-grouped validation, together with explicit leakage control and applicability-domain assessment, was documented in one study [16]; across the remaining studies, these procedures were not consistently documented in the existing evidence charting and therefore could not be reliably quantified. These findings reinforce the distinction between predictive performance within sampled data and demonstrated generalization to previously unseen compounds, adsorbents, or experimental conditions.

Beyond the choice of machine learning algorithms and validation strategies, interpreting predictive performance in adsorption systems requires considering dataset quality and model development procedures. In literature-derived datasets, consistency in measurement units, experimental conditions, and descriptor definitions facilitates meaningful comparisons across adsorbent–pollutant systems. Repeated measurements or records from related experimental series can provide valuable information about adsorption behavior, although their dependence should be considered when constructing training and evaluation partitions. Similarly, data leakage can be minimized by restricting data-dependent preprocessing and feature selection to the training data and reserving evaluation data for independent performance assessment. Feature-selection strategies, including correlation analysis, regularization, and model-based importance, can improve descriptor parsimony and interpretability, while systematic hyperparameter optimization can enhance model performance when conducted within an appropriate validation framework. Transparent reporting of these procedures strengthens reproducibility and facilitates comparisons across studies. These considerations complement the validation strategies summarized in Table S1 and provide additional context for assessing predictive performance, reproducibility, and the scope of model generalization.

SHAP, partial dependence plots (PDP), feature importance, and sensitivity analysis provide complementary approaches for interpreting adsorption ML models. These methods identify influential predictors and characterize how model outputs vary within the represented data domain, potentially revealing chemically meaningful associations. Their mechanistic relevance is strengthened when the resulting interpretations are examined alongside independent evidence from spectroscopy, controlled perturbations, DFT/MD calculations, or targeted experiments. Explainable AI can thus contribute to the formulation and refinement of testable mechanistic hypotheses, connecting predictive interpretation with molecular and experimental investigation.

Taken together, these methodological considerations highlight four distinct questions: Can the model predict the represented observations? Can its predictions be interpreted? Are the interpreted relationships chemically plausible? Do those relationships persist outside the original data domain? Treating these as separate endpoints prevents a high-accuracy model from being mistaken for a transferable mechanistic model.

### 6.2. Mechanistically Corroborated ML Is Stronger than Post Hoc Feature Interpretation

A smaller but scientifically important group of studies links data-driven predictions with molecular calculations or independent experimental evidence. Several integrated studies converge on a common workflow: ML identifies influential variables or favorable conditions; molecular calculations interrogate candidate binding sites or electronic interactions; and experiments test the resulting hypothesis [8,15,18,19].

The Sb study is a closed-loop example because interpretable ML is followed by DFT analysis of functional group/speciation interactions and targeted experimental testing [19]. Guo et al. likewise combine molecular electronic information with interpretable ML for PFAS adsorption [15], while Mou et al. connect DFT energetics, post-adsorption characterization, and SHAP attribution for morphine adsorption [18]. These examples illustrate the transition from “explainable prediction” to “mechanistically corroborated prediction.”

### 6.3. External and Grouped Validation Define a More Demanding Standard of Generalization

Several studies include external validation or independent experimental checks, but the design and chemical distance of those tests vary. External validation has been reported for carbon-material selection, PPCP adsorption, organophosphorus pesticide adsorption, Fe-modified biochar, antibiotic adsorption, and mobile organic compounds [16,80,82,84,100,101,102], whereas other studies, including those addressing hydrochar dye adsorption and PFAS removal, reported independent experimental validation [14,101].

Within the retained corpus, Zhang et al. [129], Sudarsan et al. [130], and Gümüş et al. [131] reported external validation in addition to internal resampling, whereas Ravindiran et al. [132] included experimental validation without establishing an independent external chemical-space test. This distinction is relevant because experimental confirmation within a represented domain and generalization to a new domain answer different questions.

Yet a randomly sampled external holdout from the same literature distribution remains less demanding than withholding entire compounds, adsorbent families, or preparation domains. Saeidi et al. therefore provide an informative test because compound-grouped validation revealed weaker performance than random partitioning for mobile organic compounds [16]. This demonstrates directly that interpolation within a mixed dataset is not equivalent to prediction of unseen chemical space.

Validation strategies can therefore be organized according to the degree and type of separation between model development data and the prediction target. Random splits and conventional K-fold cross-validation primarily assess interpolation, whereas grouped chemical/material validation, genuinely independent external datasets, and prospective experiments test distinct forms of out-of-domain generalization. These strategies should not be interpreted as a rigid hierarchy, because their evidentiary strength depends on what information is withheld and how far the validation domain differs from the training domain.

### 6.4. Prospective Validation and the Distinct Role of Causal Inference

Prospective experimental validation provides a strong bridge from retrospective prediction to design when it tests genuinely model-generated recommendations under conditions not used during model development. Recent workflows have experimentally evaluated model-guided material choices, preparation conditions, or adsorption predictions [101,102,103,104]. Causal inference addresses a distinct question and should not be treated as a higher level of predictive validation. Whereas prospective validation evaluates whether predictions persist when tested on previously unseen outcomes or conditions, causal inference seeks to estimate intervention-relevant effects under explicit assumptions about the data-generating process. Causal approaches remain uncommon in the retained corpus; Zhao et al. [80], for example, applied double machine learning to estimate causal effects after predictive modeling. Such analyses may strengthen intervention-oriented interpretation, but causal estimates do not by themselves establish a molecular adsorption mechanism and still require independent chemical or molecular corroboration when mechanistic claims are made.

Table 5 distinguishes interpolation, out-of-domain generalization, prospective validation, mechanistic corroboration, and causal inference to prevent predictive performance, transferability, mechanistic evidence, and causal identification from being conflated.

### 6.5. RQ5 Synthesis: From Predictive Accuracy to Transferable Design

Predictive literature can be interpreted in terms of four distinct outcomes: predictive performance, model interpretability, mechanistic corroboration, and transferable design. Predictive performance and model interpretation are widely represented in the retained corpus, whereas studies combining prediction with independent mechanistic corroboration or demonstrating prospective transfer to previously unseen systems are less common.

The central RQ5 conclusion is therefore that ML does not transform a proposed adsorption mechanism into a predictive mechanism merely by adding SHAP to a high-performing model. The transition requires chemically meaningful descriptors, explicit validation beyond the represented training distribution, independent molecular or experimental corroboration, and ideally prospective testing. In this sense, predictive transferability constitutes a distinct MMEF dimension that captures whether mechanistically interpretable relationships persist beyond the domain in which they were developed.

## 7. Molecular Mechanistic Evidence Framework (MMEF): From Proposed to Predictive Mechanisms

### 7.1. Why a Multidimensional Framework Is Needed

The cross-study synthesis shows that the central problem in the literature is not a shortage of proposed mechanisms, but the unequal depth, independence, realism, and predictive reach of the evidence used to support them. RQ1 identified a recurring but conditional mechanistic vocabulary; RQ2 showed that macroscopic model fitting is still sometimes overextended into molecular claims; RQ3 demonstrated that molecular evidence ranges from indirect descriptors to experimentally constrained dynamic models; and RQ4–RQ5 showed that descriptor importance, interpretability, mechanistic plausibility, and transferability are distinct outcomes. These findings motivate the Molecular Mechanistic Evidence Framework (MMEF) as an emergent conceptual framework rather than a preassigned scoring system.

The MMEF is organized around five principal axes: molecular depth, evidence convergence and independence, environmental realism, interpretive discipline, and predictive transferability.

### 7.2. The Five MMEF Dimensions

To operationalize the framework without converting it into a numerical score, Table 6 defines the core question and the types of evidence associated with each MMEF dimension.

**Figure 3:**
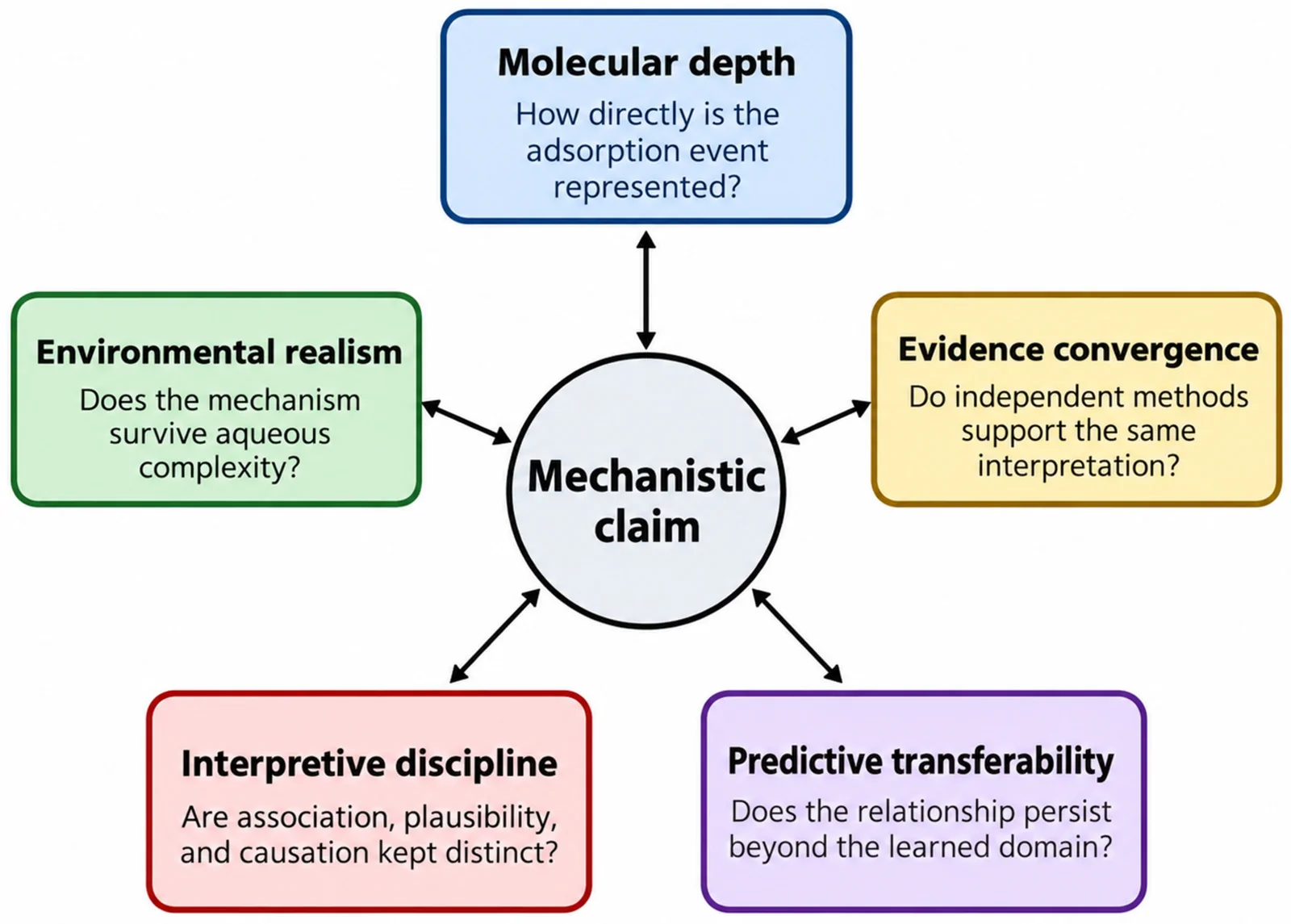
Molecular Mechanistic Evidence Framework (MMEF). Five partially independent dimensions organize the evidence supporting a mechanistic claim; the framework is not a numerical quality score.

The relationships among the five partially independent MMEF dimensions are represented schematically in Figure 3.

The axes are intentionally non-equivalent: a study may be molecularly sophisticated but environmentally idealized, experimentally rich but interpretively overextended, or highly predictive within-domain without demonstrating transferability. Anchor studies illustrate different strengths across these dimensions, including experimentally constrained MD [10,11], convergent experiment–DFT–ML workflows [15,18], and closed-loop predictive testing [19].

To demonstrate the practical application of the MMEF, Table S2 presents a qualitative assessment of representative studies exhibiting contrasting evidence profiles. These examples encompass strong experiment–theory convergence, incomplete molecular representation, and instances in which independently supported mechanisms coexist with overinterpretation of kinetic or equilibrium model fitting. By examining each MMEF dimension separately, the analysis identifies study-specific strengths, evidentiary limitations, and opportunities for further validation without assigning numerical scores or overall quality rankings.

### 7.3. From Proposed to Predictive Adsorption Mechanisms

The five axes support a conceptual progression from proposed mechanisms to independently supported, molecularly resolved, environmentally tested, and ultimately transferable predictive mechanisms. This progression is not a mandatory five-level hierarchy: studies may advance unevenly along different axes, and strong mechanistic science does not require ML. The central implication is instead that mechanistic sophistication is not synonymous with evidentiary completeness. Molecular depth, evidence convergence, environmental realism, interpretive discipline, and predictive transferability vary partially independently and should be reported as such.

## 8. Limitations, Future Perspectives, and Research Roadmap

### 8.1. From Mechanism Enumeration to Mechanism Discrimination

The main gap is not the generation of plausible mechanisms but their discrimination. Future studies should define competing hypotheses before model fitting and identify observations capable of distinguishing among electrostatics, hydrogen bonding, π interactions, confinement, coordination, ion exchange, precipitation, and redox processes. Kinetic and isotherm models should remain descriptive; molecular claims should rely on independent chemical, spectroscopic, perturbational, or computational evidence. This recommendation follows directly from the RQ2 audit, which identified confirmed local fit-to-mechanism overinterpretation in 32 of 70 retained mechanistic studies.

### 8.2. Molecular Models Should Represent the Aqueous Interface They Are Used to Explain

The next computational advance is stronger model–system correspondence rather than simply more DFT. Surface heterogeneity, relevant functional groups and defects, pollutant and surface protonation, solvation, pore confinement, and competing species should be represented when they are material to the mechanistic claim. Dynamic MD and sampling remain underused (9/70 mechanistic studies), despite their ability to resolve diffusion, pore entry, hydration, ion bridging, competition, and interfacial organization. Experimentally constrained models provide a particularly promising route [10,11].

### 8.3. Environmental Complexity Should Be Treated as Mechanistic Evidence

pH, ionic strength, dissolved organic matter, competing ions, co-solutes, and real-water matrices can change speciation, site accessibility, screening, bridging, and the balance between adsorption and transformation. Mechanisms inferred in ultrapure single-solute systems should therefore be stress-tested under chemically relevant conditions. Multicomponent studies already demonstrate that solution composition can alter the operative adsorption pathway rather than merely reduce capacity [4,51,52,75].

### 8.4. Transferability Requires Domain-Aware Validation

No universal adsorption descriptor emerged. Transferable models should instead combine chemically meaningful material, molecular, and environmental descriptor families and explicitly test whether learned relationships persist outside the represented domain. Grouped, external, and prospective validation should therefore replace random-split performance as the primary evidence of generalization (Section 6.3; Table 5).

### 8.5. Close the Prediction–Mechanism–Experiment Loop

A useful future workflow is prediction → testable molecular hypothesis → molecular calculation → targeted experiment → redesign. XAI should identify hypotheses, not certify mechanisms, and causal terminology should be reserved for designs that support causal identification. Integrated studies already demonstrate the feasibility of this loop [8,15,18], with the Sb-removal workflow providing a particularly clear example of ML-guided interpretation followed by DFT and targeted experimental testing [19].

The principal gaps identified across RQ1–RQ5 and the corresponding methodological shifts are synthesized in the research roadmap presented in Table 7.

### 8.6. Integrated Roadmap

**Figure 4:**
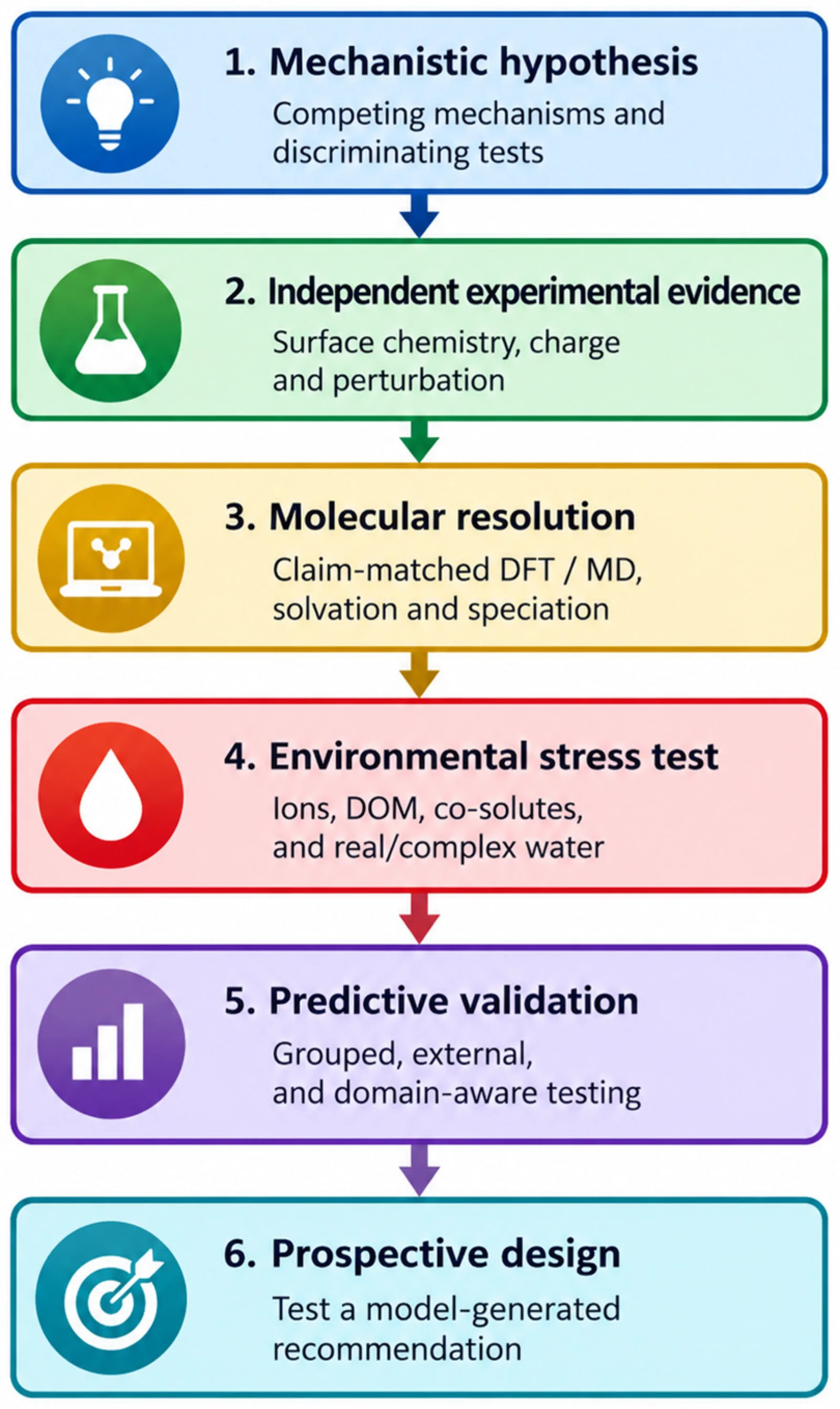
Research roadmap from mechanistic hypothesis to prospective adsorbent design. The pathway represents a research workflow rather than a ranking system.

The resulting sequence from mechanistic hypothesis to prospective adsorbent design is summarized as an integrated workflow in Figure 4.

Across RQ1–RQ5, the evidence supports an integrated research workflow connecting mechanistic hypothesis generation, independent experimental evidence, molecular resolution, testing under environmentally relevant conditions, predictive validation, and, where appropriate, prospective design. These components are complementary rather than mandatory sequential stages, and their relevance depends on the research question being addressed. Their integration can increase mechanistic discrimination, environmental relevance, and predictive transferability. The broader objective is not merely to explain retrospectively why an adsorbent performed as observed, but to use convergent evidence to inform predictions about which combinations of surface chemistry, pore architecture, pollutant–surface interactions, and operating conditions are most likely to perform effectively in previously untested systems.

### 8.7. Scope and Limitations of the Retrieval Pool

The final corpus was constructed through relevance-prioritized evidence charting rather than exhaustive full-text assessment of all 488 potentially eligible reports. Consequently, the review should not be interpreted as an exhaustive census of the adsorption literature. Studies assigned lower priority during title/abstract screening may contain relevant evidence that could alter the relative frequencies of particular mechanisms, descriptor families, or validation practices.

Although the retrospective thematic assessment of the 363 potentially eligible reports not retained in the analytical corpus did not identify additional recurrent evidence domains requiring revision of the synthesis (Table S3), this abstract-level assessment cannot exclude isolated, contradictory, or more detailed findings in the unexamined full texts. Residual selection bias therefore remains possible, particularly because prioritization favored studies with stronger apparent relevance to the mechanistic and predictive research questions. Accordingly, the principal conclusions should be interpreted as a conceptual synthesis of the selected evidence rather than as exhaustive or statistically representative estimates of the field. The selection procedure and operational basis for analytic closure are detailed in Section 9.4.

## 9. Methodology: Systematic Evidence Mapping and Critical Mechanistic Synthesis

### 9.1. Review Design and Search Strategy

This study was designed as a scoping evidence review combining systematic evidence mapping with critical mechanistic synthesis rather than as an exhaustive systematic review or a meta-analysis of adsorption capacity. The analytical unit was the adsorbent–pollutant interaction and the evidence supporting a mechanistic or predictive claim. Five questions guided the synthesis: mechanisms reported (RQ1), evidentiary independence and overinterpretation (RQ2), contribution of DFT/MD (RQ3), transferable descriptors (RQ4), and conditions under which ML/explainable artificial intelligence (XAI) supports generalizable prediction (RQ5). Because the objective was to map the scope, characteristics, and convergence of heterogeneous evidence rather than estimate a pooled effect, reporting followed the PRISMA-ScR (Preferred Reporting Items for Systematic reviews and Meta-Analyses extension for Scoping Reviews) framework [133]. PRISMA-ScR was used as a reporting framework to enhance transparency of the search, screening, evidence-charting, and synthesis procedures; its use should not be interpreted as implying exhaustive full-text resolution of every record within the potentially eligible pool. The completed PRISMA-ScR checklist and the Sequential Database Search Strategies (Scopus M1–M3 and P; Web of Science M and P; search date 31 August 2026) are provided in Table S4 and Table S5, respectively.

No formal review protocol was developed or registered prospectively. The initial review scope, research questions, bibliographic sources, search concepts, and broad eligibility boundaries were established to guide identification and screening. The relevance-prioritization scheme, targeted evidence enrichment, detailed charting rules, and operational criteria for analytic closure were subsequently refined and documented during the review. The molecular evidence archetypes and the Molecular Mechanistic Evidence Framework (MMEF) were developed through evidence analysis and synthesis rather than specified prospectively. Accordingly, these methodological decisions are reported according to their stage of development and are not presented as components of a preregistered protocol.

Scopus and Web of Science Core Collection formed the bibliographic core. The searches were conducted on 31 August 2026, without restrictions on publication year or language. Accordingly, the search window extended to the search date, and non-English records were not excluded through database language filters. Two complementary searches were constructed: Corpus M combined biomass-derived adsorbent, aqueous adsorption/pollutant, and DFT/MD/molecular simulation terms; Corpus P retained the material and aqueous-pollutant blocks but used ML/XAI and molecular/quantum descriptor terms. The validated Scopus searches yielded 536 M and 191 P records; Web of Science yielded 100 M and 81 P records. Across databases, 908 raw records were identified and 828 unique records remained after DOI/title-based deduplication, with database and M/P provenance preserved.

### 9.2. Eligibility, Screening, and Evidence Enrichment

Primary studies were eligible when adsorption was a substantive process in an aqueous system, the adsorbent was biomass-derived (including biochar, hydrochar, biomass-derived activated carbon, lignocellulosic materials, and modified/composite adsorbents with a predominance of biomass), and the study provided relevant molecular or predictive evidence for research questions 1–5. Gas-phase or soil-only studies, adsorbents not derived from biomass, and systems with a predominance of degradation and no independently analyzable adsorption were excluded. Reviews and non-primary formats were retained for methodological context or general information purposes only.

**Figure 5:**
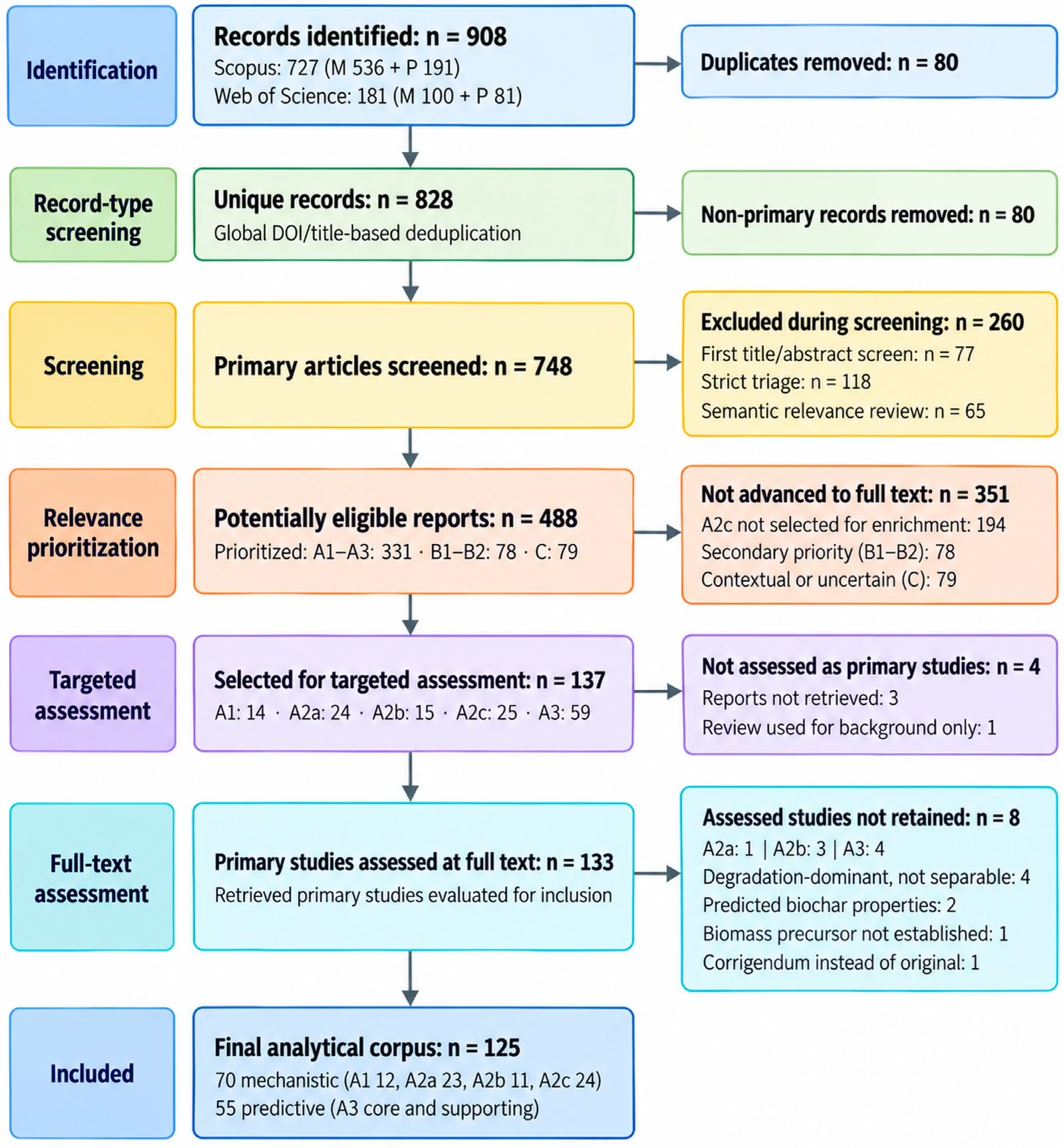
PRISMA-ScR-informed flow diagram of study identification, screening, relevance-based prioritization, and final analytical corpus construction. Records not advanced to full-text assessment were not classified as formal full-text exclusions.

After removing duplicates and non-primary records, sequential title/abstract screening, strict triage, and semantic assessment identified 488 potentially eligible reports (Table 8; Figure 5). These records were classified according to their anticipated contribution to the review questions. The classification distinguished 331 higher-priority candidates (A1–A3), 78 secondary-priority candidates (B1–B2), and 79 contextual or uncertain records (C). Secondary-priority records comprised predictive comparators (B1) and studies whose molecular contribution required further confirmation (B2), whereas category C included records requiring additional clarification of their relevance or eligibility. These prioritization categories were operationalized during evidence mapping to guide retrieval and charting rather than being prospectively specified eligibility criteria or formal full-text exclusions.

Within the high-priority group, A1 identified studies that integrated molecular and predictive evidence; A2a and A2b distinguished molecular-mechanistic candidates based on the specificity of the reported evidence; Category A2c comprised low-priority molecular candidates, and A3 identified predictive or explainable modeling studies. Full-text appraisal prioritized categories A1, A2a, A2b, and A3, supplemented by the purposive selection of 25 A2c candidates to strengthen coverage of explicit water molecular dynamics, pH-dependent speciation, competitive adsorption, complex aqueous matrices, and advanced molecular interaction analysis. This strategy generated 137 records selected for targeted appraisal.

Of the 488 potentially eligible reports, 125 primary studies were retained in the analytical corpus (70 mechanistic and 55 predictive; Table 8). The remaining 363 reports comprised three distinct groups: (i) 351 records that were not advanced to full-text assessment (194 A2c candidates not selected for targeted enrichment, 78 secondary-priority B1–B2 candidates, and 79 contextual or uncertain C records); (ii) four selected records that did not undergo full-text evaluation as primary studies, comprising three reports that could not be retrieved and one review used for background information only; and (iii) eight of the 133 primary studies assessed at full text that were not retained (A2a, *n* = 1; A2b, *n* = 3; A3, *n* = 4). Only the studies in group (iii) constitute full-text exclusions. Their reasons for exclusion, which reflect the eligibility criteria described above rather than judgments on study quality, are reported in Table 8 and Figure 5. The 351 records in group (i) were not evaluated for full-text eligibility and are therefore not reported as full-text exclusions. Analytic closure was based on the stabilization of the evidence categories identified after targeted enrichment, rather than on a predetermined number of included studies (Section 9.4). To examine potential selection effects, the titles, abstracts, and original screening classifications of all 363 non-retained reports were retrospectively assessed. This analysis did not identify any additional recurrent mechanistic families or broad methodological domains that would require revision of the synthesis (Table S3). The resulting limitations regarding selection bias and generalizability are discussed in Section 8.7.

### 9.3. Evidence Extraction and RQ-Specific Audits

Evidence extraction and classification were performed by the authors using structured evidence-charting workbooks. AI-assisted tools were used to support literature organization, retrieval, and preliminary classification but did not make final eligibility, evidence-coding, or interpretive decisions. All AI-assisted outputs used in the evidence map were reviewed and verified by the authors against the source material. Ambiguous cases were re-examined at the full-text level, and final classifications were resolved by author consensus.

Mechanistic extraction captured adsorbent/pollutant identity, proposed interactions, kinetics/isotherms, surface and charge characterization, DFT/MD representation and energetics, solvent and speciation treatment, real/complex water testing, and experiment–theory agreement. Predictive extraction additionally captured dataset provenance, descriptor families, model class, splitting and validation design, XAI, applicability-domain or leakage controls, and independent molecular or experimental corroboration. Evidence coding distinguished mechanisms proposed by the original authors from mechanisms supported by convergent experimental or molecular evidence. Mechanistic labels were recorded as non-exclusive categories, whereas molecular evidence archetypes were assigned according to the primary model–system representation. Predictive-validation strategies were classified according to the reported data-partitioning and testing procedures, without assuming external transferability from internal validation alone. Claim-level overinterpretation was assessed separately from the overall plausibility of the proposed mechanism. The operational definitions, assignment criteria, and treatment of ambiguous or incompletely reported evidence are summarized in Table S6.

Question-specific audits were used instead of a single quality score. For RQ2, a fit-to-mechanism claim was classified as confirmed only when the full text explicitly used kinetic or equilibrium model fitting to infer chemical versus physical adsorption or another microscopic adsorption mechanism. Candidate claims were identified through a rule-based screen of the 70 mechanistic studies and through the evidence-charting notes; candidates that did not meet this criterion upon full-text verification were classified as false positives and excluded from the confirmed counts, and candidates whose full text could not be verified were left unresolved and not counted; RQ3 classified molecular evidence by model–system correspondence from indirect descriptors to experimentally constrained MD. The M0–M5 archetypes were assigned as mutually exclusive primary categories of molecular model–system correspondence, whereas selected attributes, including solvent awareness, were coded independently as cross-cutting characteristics and could therefore span more than one archetype. RQ4 grouped descriptors into textural, surface-chemical, charge/acid–base, molecular, electronic/fingerprint, and environmental families; and RQ5 distinguished interpolation from grouped, external, and prospective validation. Counts and percentages were descriptive and overlapping where studies contributed more than one mechanism or validation practice. All frequencies and percentages reported in the synthesis describe the retained relevance-prioritized analytical corpus and should not be interpreted as prevalence estimates for the complete body of potentially eligible literature.

### 9.4. Emergent Framework and Methodological Status

The Molecular Mechanistic Evidence Framework (MMEF) was developed inductively during evidence synthesis and was not used to determine study eligibility, relevance priority, or inclusion. Initial evidence charting was guided by RQ1–RQ5 and broad analytical domains covering reported adsorption mechanisms, independent mechanistic support, molecular modeling, descriptor families, and predictive validation. More specific coding distinctions, including molecular evidence archetypes and claim-level interpretive rules, were refined as the evidence was examined and subsequently applied consistently across the retained corpus. Cross-domain synthesis then identified five partially independent dimensions—molecular depth, evidence convergence, environmental realism, interpretive discipline, and predictive transferability—which were integrated into the MMEF. The framework therefore represents an interpretive outcome of the review rather than a prospectively defined assessment instrument. Search provenance, screening decisions, extraction, audits, and citation coverage were documented in structured workbooks.

The study-identification, screening, prioritization, and corpus-construction process is documented in Table 8 and illustrated in Figure 5.

Analytic saturation was assessed during relevance-prioritized evidence charting as the observed stabilization of analytical categories, rather than through a prospectively specified numerical stopping threshold. Charting was closed after the higher-priority strata had been examined and targeted A2c enrichment no longer introduced substantively new evidence categories. Neither the subsequently developed MMEF dimensions nor a fixed number of consecutive studies without new categories was used as an a priori stopping rule.

Targeted A2c enrichment focused on explicit water molecular dynamics, pollutant speciation, competitive adsorption, environmentally relevant aqueous matrices, and advanced molecular analysis. The recurring adsorption-mechanism categories were already represented before this stage, whereas two additional molecular evidence archetypes, M1b and M5, emerged during enrichment. Subsequent inclusions did not introduce further mechanistic categories or molecular evidence archetypes and did not materially expand the descriptor families or predictive-validation strategies. This stabilization provided the basis for closing the analytical corpus at 125 studies (Table S7).

Analytic saturation therefore supports the conceptual coverage of the relevance-prioritized evidence map, not statistical representativeness, exhaustive coverage, or literature-wide prevalence estimates; the composition of the 363 non-retained reports and their retrospective thematic assessment are reported in Section 9.2 and Table S3.

## 10. Conclusions

This review shows that advancing the understanding of adsorption mechanisms in biomass-derived adsorbents requires moving beyond the enumeration of plausible interactions toward their discrimination through convergent evidence. Electrostatic interactions, hydrogen bonding, surface complexation or coordination, π interactions, and pore filling or confinement recur across the literature, but their contribution depends on surface chemistry, pore architecture, pollutant speciation, and aqueous conditions. Consequently, adsorption mechanisms cannot be reliably established from kinetic or isotherm fitting alone; macroscopic models should remain descriptive unless supported by independent chemical, spectroscopic, or molecular evidence.

Molecular simulation can strengthen mechanistic interpretation when the computational representation corresponds to the experimental system. However, the reviewed evidence shows a predominance of static adsorbate–surface DFT, whereas solvent/speciation-aware calculations, dynamic MD, and experimentally constrained models remain less developed. Likewise, predictive accuracy and XAI attribution should not be equated with mechanistic understanding or transferability. Chemically meaningful descriptors combined with grouped, external, or prospective validation provide a more appropriate basis for evaluating whether learned relationships extend beyond the training domain.

These findings should be interpreted within the scope of the review design. The synthesis was based on a relevance-prioritized analytical corpus of 125 primary studies rather than an exhaustive full-text assessment of all potentially eligible records. Consequently, the reported frequencies and evidence patterns characterize the retained corpus and should not be interpreted as statistically representative estimates of the broader literature. Although the retrospective assessment of the 363 non-retained reports supported the stability of the principal analytical categories, the possibility of additional findings in studies not examined in full cannot be excluded. These considerations define the scope of the evidence map without diminishing its contribution to identifying recurring mechanistic patterns and methodological priorities.

Building on the evidence map findings, we propose the Molecular Mechanistic Evidence Framework (MMEF), which integrates five complementary and partially independent dimensions: molecular depth, evidence convergence, environmental realism, interpretive discipline, and predictive transferability. Rather than providing a numerical quality score or a mandatory hierarchy, the MMEF offers a common structure for evaluating how different forms of evidence contribute to mechanistic confidence and predictive relevance.

For studies seeking progress from mechanistic interpretation toward predictive adsorbent design, a useful research workflow can connect mechanistic hypothesis generation, independent experimental testing, molecular resolution, evaluation under environmentally relevant conditions, predictive validation, and, where appropriate, prospective design. These elements need not occur as fixed sequential stages or be present in every study; rather, their integration can strengthen the correspondence between molecular interpretation, experimental evidence, and predictions beyond the conditions used for model development.

## Figures and Tables

**Table 1:** Recurring adsorption mechanisms, diagnostic evidence, and representative systems in biomass-derived adsorbents.

Mechanism	Typical Systems	Evidence that Strengthens Assignment	Key Interpretive Warnings	References
Electrostatic interactions	Ionizable organics, PFAS, metals/oxyanions	pH dependence, pH_pzc_/zeta potential, speciation and charge-state analysis; molecular electrostatics	Charge effects are conditional on pH, ionic strength and adsorbate/surface speciation.	[2,38,48]
Hydrogen bonding	Pharmaceuticals, antibiotics, dyes, phenolic organics	FTIR/XPS changes plus explicit donor–acceptor geometries or noncovalent interaction (NCI)/DFT evidence	Usually coexists with π interactions, electrostatics or dispersion; spectral shifts alone are non-unique.	[1,3,55]
Surface complexation/coordination	Metals, oxyanions, metal-binding organics	XPS/FTIR chemical-state changes, site-specific perturbation, DFT binding/charge-transfer evidence	Must be distinguished from ion exchange, precipitation and nonspecific electrostatics.	[17,41,56]
π-mediated interactions	Aromatic organics, antibiotics, dyes	Aromatic surface domains plus adsorption perturbation and DFT/NCI geometry/electronic evidence	Aromaticity or graphitization alone does not demonstrate a specific π interaction.	[21,30,57]
Pore filling/confinement	Organic micropollutants and size-selective adsorption systems	Pore-size distribution + molecular dimensions; explicit pore models or MD provide stronger support	BET/specific surface area (SSA) alone does not prove pore filling; accessibility depends on molecular size, hydration and surface chemistry.	[9,10,11]
van der Waals/NCI	Neutral/aromatic organics and hydrophobic domains	NCI/reduced density gradient (RDG) analysis, dispersion-aware DFT and molecular contact/orientation analysis	Frequently contributory rather than uniquely dominant; difficult to isolate experimentally.	[24,58,59]
Hydrophobic interactions	Hydrophobic organics and PFAS	Hydrophobicity trends, carbon-domain chemistry and solvent-aware molecular evidence	May be confounded with pore affinity, dispersion and fluorocarbon–carbon interactions.	[15,37,60]
Redox/transformation	Cr(VI), redox-active metals and selected organics	Oxidation-state-resolved spectroscopy/speciation plus mass balance and molecular support	Removal from solution must be separated from adsorption-coupled chemical transformation.	[2,47,53]
Ion exchange/bridging	Metals, phosphate, ammonium and multicomponent systems	Ion release/uptake, elemental analysis, competition tests and coordination-aware molecular evidence	Solution composition can switch the operative pathway; bridging requires evidence for the mediating ion/site.	[42,61,62]
Precipitation	Metals and phosphate-rich systems	Solid-phase identification, solution mass balance and speciation/mineral evidence	A decrease in dissolved concentration is not sufficient to label the process adsorption.	[42,47,62]

**Table 2:** Molecular evidence archetypes according to model–system correspondence.

Level	Molecular Representation	Representation of the Aqueous Environment	Mechanistic Contribution	Principal Limitation	Ref.
M0	No direct molecular adsorption simulation	—	Experimental and surface-chemical evidence without direct molecular simulation	No direct molecular resolution of the adsorbate–surface interaction	[39,69,70]
M1	Indirect molecular descriptors or isolated-molecule calculations	No explicit adsorbate–surface aqueous interface	Molecular properties, reactive regions, molecular size, electrostatic potential, or frontier-orbital characteristics	No explicit adsorbate–surface interaction	[71,72,73]
M1b	Statistical-physics or non-atomistic molecular interpretation	No explicit atomistic aqueous interface	Thermodynamic or statistical interpretation of adsorption at the molecular level	No atomistic representation of the adsorbate–surface interface	[26]
M2	Explicit adsorbate–surface static DFT	Aqueous environment absent or represented only indirectly	Binding sites, adsorption geometries and energies, charge transfer, and noncovalent interactions	Static representation; solvent, speciation, and interfacial dynamics may be omitted	[34,55,58]
M3	Solvent- and/or speciation-aware surface DFT	Solvent and/or relevant adsorbate speciation explicitly considered in the molecular model	Evaluation of adsorption configurations and energetics under solution-relevant molecular states	Static treatment limits sampling of interfacial configurations and dynamics	[8,29,74]
M4	Dynamic interfacial MD or explicit water molecular model	Explicit water and dynamic adsorbate–surface environment represented	Hydration, diffusion, confinement, competition, and time-dependent interfacial organization	Results depend on structural representation, force field, simulation time, and sampling	[9,57]
M5	Experimentally constrained explicit water MD	Explicit aqueous environment combined with experimentally informed adsorbent representation	Material-specific dynamic interpretation connecting experimental descriptors with molecular adsorption behavior	Requires experimentally informed structural models and substantial computational sampling	[10,11]

Note: DFT, density functional theory; MD, molecular dynamics; —, not applicable. The M0–M5 categories describe differences in molecular representation and model–system correspondence; they should not be interpreted as a numerical quality score or as a mandatory hierarchy of study quality.

**Table 3:** Evidentiary strength and inferential limits of approaches used to assign adsorption mechanisms.

Evidence Source	What It Can Establish	What It Cannot Establish Alone	Strongest Use	Common Inferential Risk	Ref.
Kinetics	Macroscopic rate behavior	Chemical vs. physical adsorption mechanism	Descriptive comparison	PSO fit → chemisorption	[5,6,78]
Isotherms	Empirical equilibrium response	Unique molecular pathway or surface architecture	Equilibrium description	Langmuir fit → proof of monolayer adsorption	[7,79]
Surface/charge characterization	Functional groups, chemical states and charge context	A unique interaction mechanism in isolation	Independent experimental convergence	Assigning every spectral/pH shift to one mechanism	[17,41,47]
Static adsorbate–surface DFT	Binding sites, geometries, energies and charge/electronic redistribution	Dynamic aqueous-interface behavior	Molecular discrimination among competing hypotheses	Idealized surface → real heterogeneous biochar mechanism	[24,55,58]
Solvent/speciation-aware DFT	Solution-relevant molecular plausibility and binding-state effects	Full interfacial dynamics	Testing chemically realistic binding states	Incomplete representation of surface heterogeneity	[8,29,38]
MD/explicit water simulation	Hydration, diffusion, pore entry, competition and dynamic organization	Electronic reaction chemistry unless explicitly modeled	Dynamic/interfacial evidence	Force-field, sampling and structural-model dependence	[9,10,11]
ML/XAI	Prediction and model-attribution patterns	Molecular causation	Hypothesis generation and predictive testing	SHAP/feature importance → mechanism	[15,19,80]

**Table 4:** Descriptor families, mechanistic meaning, and evidence for predictive transferability.

Descriptor Family	Representative Variables	Mechanistic Meaning	Transferability Requirement	Key Caution	Ref.
Textural/pore	SSA, pore volume, pore size, micro-/mesoporosity	Accessibility, confinement and transport	Persistence across adsorbent/material domains; preferably linked to molecular size or pore models	SSA alone does not prove pore filling	[9,10,85]
Surface chemistry/charge	O/C, N/C, XPS groups, pHpzc, zeta potential, cation-exchange capacity (CEC), metal/mineral sites	Electrostatics, H-bonding, coordination and ion exchange	Stable direction across pH/material domains and independent chemical corroboration	Thresholds and importance are material- and pH-dependent	[17,56,84]
Molecular physicochemical	MW/volume, logKOW/logKOC, pKa, aromaticity, HBD/HBA	Compatibility, hydrophobicity, speciation and steric accessibility	Validation on unseen compounds/chemically grouped splits	Requires adequate chemical-space coverage	[14,16,99]
Structural fingerprints	extended-connectivity fingerprints (ECFP)/Morgan and related substructure encodings	Representation of molecular structural diversity	Compound-grouped or independent external validation	Predictive but mechanistically opaque unless linked to interpretable chemistry	[14,15,86]
Quantum/electronic	Eads, ESP, charges, highest occupied molecular orbital/lowest unoccupied molecular orbital (HOMO/LUMO), dipole, density of states (DOS)/NCI	Site affinity, charge transfer and electronic/noncovalent interactions	Consistency across molecular/material domains plus experimental corroboration	Model-dependent; not invariant material properties	[8,15,19]
Operational/environmental	pH, concentration, dose, temperature, ionic strength, dissolved organic matter (DOM)/co-solutes	Context controlling expressed adsorption behavior	External/real-water or condition-shift validation	Predictive importance is not an intrinsic molecular mechanism	[14,80,88]

**Table 5:** Validation strategies, domain separation, and implications for predictive transferability.

Validation Strategy	What Is Withheld/Tested	Question Answered	Validation Domain Separation	Mechanistic Value/Caution	Ref.
Random train/test split	Random observations from represented data	Can the model interpolate within the sampled distribution?	Random observation-level separation	None by itself	[99,105]
Conventional K-fold cross-validation (CV)	Random folds from the same data distribution	Is interpolation stable across resampling?	Random fold-level separation	None by itself	[85,86]
Compound-grouped validation	Entire compounds/chemical identities	Can the model generalize to unseen chemical space?	Compound-level separation	Tests molecular-descriptor generalization	[16]
Independent external dataset	New dataset/source/domain	Does performance persist outside model development data?	Dataset- or domain-level separation	Strengthens predictive—not automatically mechanistic—credibility	[16,82,84,100]
Independent experimental validation	New experiments under predicted conditions	Does a model prediction survive empirical testing?	Experimental separation; domain overlap may vary	Can test predictive hypotheses; mechanistic value depends on experimental design	[101,102,103,104]
Mechanistically corroborated ML	Prediction/XAI + spectroscopy, perturbation, DFT/MD or targeted experiment	Is the learned association chemically consistent with independent evidence?	Not intrinsic to mechanistic corroboration	Avoids equating XAI attribution with causation	[8,15,18]
Closed-loop prospective design	Model-generated hypothesis → molecular test → targeted experiment	Can prediction guide a new design decision before observation?	Prospective separation of new design outcomes	Requires prospective separation from training information	[19]
Causal inference	Explicit causal estimation design	Does the analysis estimate an intervention-relevant effect under stated assumptions?	Not intrinsic to causal inference	Causal ML still requires chemical corroboration for molecular-mechanism claims	[80]

Note: Validation domain separation describes the type of information withheld or independently evaluated, not a ranking of study quality. Its implications for transferability depend on data independence and domain differences. Mechanistic corroboration and causal inference address distinct questions and do not inherently establish predictive transferability.

**Table 6:** Operational interpretation of the five MMEF dimensions.

MMEF Dimension	Core Question	Evidence Indicating Greater Support
Molecular depth	How directly does the model represent adsorption?	Explicit adsorbate–surface representation; solvent/speciation-aware modeling; dynamic and/or experimentally constrained interfacial modeling
Evidence convergence	Do independent methods support the same interpretation?	Charge/spectroscopy/perturbation + molecular energetics/dynamics
Environmental realism	Does the mechanism survive aqueous complexity?	Speciation, ions, DOM, co-solutes, real/complex water
Interpretive discipline	Are description, association and causation kept distinct?	Model/XAI outputs framed as support or hypotheses unless independently corroborated
Predictive transferability	Does the relationship persist beyond the training domain?	Grouped validation; genuinely independent external validation; prospective testing

**Table 7:** Research roadmap derived from RQ1–RQ5 and the MMEF.

Current Limitation	Recommended Methodological Shift	Evidence Gained	MMEF Axis Primarily Strengthened
Fit-derived mechanism labels	Use kinetics/isotherms descriptively; require independent mechanistic evidence	Interpretive validity	Interpretive discipline
Idealized static surface models	Experimentally informed surfaces; solvation/speciation-aware DFT	Model–system correspondence	Molecular depth
Single optimized geometries	Add MD/sampling for pore transport, competition and hydration	Dynamic interfacial evidence	Molecular depth/environmental realism
Ultrapure single-solute tests	Use ions, DOM, competitors and real/complex waters	Conditional mechanism and robustness	Environmental realism
Correlated evidence streams	Triangulate spectroscopy, perturbation experiments and molecular calculations	Independent convergence	Evidence convergence
Random-split ML validation	Grouped, external and prospective validation	Out-of-domain performance	Predictive transferability
SHAP/PDP treated as mechanism	Use XAI for hypotheses; corroborate chemistry, DFT/MD or experiments	Mechanistic plausibility	Interpretive discipline/convergence
Retrospective prediction only	Close ML → molecular hypothesis → experiment → redesign loop	Actionable design knowledge	Predictive transferability/convergence

**Table 8:** Search and screening traceability and construction of the final relevance-prioritized evidence corpus.

Stage	Records	Interpretation
Scopus M/P	536/191	Validated molecular and predictive searches
Web of Science M/P	100/81	Molecular and predictive exports
Raw database records	908	Before global deduplication
Unique records	828	After DOI/title-based global deduplication
Primary articles screened	748	After removal of 80 non-primary records
Advanced after first title/abstract screen	671	659 include + 12 uncertain
Retrieval candidates after strict triage	553	454 full-text + 99 uncertain
Potentially eligible after semantic review	488	65 clear abstract-level exclusions
Higher-priority evidence candidates (A1–A3)	331	Prioritized for retrieval based on direct molecular, integrated molecular–ML, or predictive contributions to RQ1–RQ5
Secondary-priority candidates (B1–B2)	78	22 predictive comparators (B1) and 56 studies requiring confirmation of their molecular contribution (B2); not advanced to full-text charting
Contextual/uncertain candidates (C)	79	Studies requiring further eligibility or relevance clarification; not advanced to full-text charting
Higher-priority candidates not selected for targeted enrichment	194	Remaining A2c candidates after selection of 25 from the original A2c pool of 219
Candidates selected for targeted assessment	137	A1 (14), A2a (24), A2b (15), A2c (25), and A3 (59)
Selected reports not retrieved	3	One each from A1, A2b, and A2c
Review retained for background only	1	A1 record not included in the primary-study corpus
Primary studies assessed at full text	133	Retrieved primary studies evaluated for inclusion in the analytical corpus
Assessed studies not retained	8	A2a (1), A2b (3), and A3 (4); reasons detailed in the table note.
Final retained mechanistic corpus	70	A1 (12), A2a (23), A2b (11), and A2c (24)
Final retained predictive corpus	55	A3 core and supporting predictive studies
Final analytical corpus	125	107 core and 18 supporting primary studies retained for synthesis

Note: M and P denote molecular and predictive search streams, respectively. Relevance-priority assignments guided retrieval and evidence charting, not formal full-text exclusion. Records not advanced to full-text assessment remained outside the analytical corpus. The eight studies assessed at full text but not retained comprised four degradation-dominant studies in which molecular calculations addressed photocatalytic or oxidative transformation pathways rather than independently characterized pollutant adsorption (A2a, *n* = 1; A2b, *n* = 3); two A3 studies predicting biochar properties rather than pollutant adsorption; one A3 study for which a predominantly biomass-derived adsorbent precursor could not be established; and one A3 record for which the retrieved document was a corrigendum rather than the original research article. These decisions reflect the eligibility criteria of the review rather than judgments on study quality. The retrospective thematic assessment of the 363 potentially eligible reports not retained (351 not advanced to full-text assessment, 3 not retrieved, 1 background review, and 8 excluded after full-text assessment) is summarized in Supplementary Table S3.

## Data Availability

No new data were created or analyzed in this study. Data sharing is not applicable to this article.

## Supplementary Materials

The following supporting information can be downloaded at https://www.mdpi.com/article/10.3390/molecules31214018/s1: Table S1: Validation strategies reported across the 55 predictive studies; Table S2: Illustrative application of the Molecular Mechanistic Evidence Framework to representative studies; Table S3: Thematic coverage of the 363 potentially eligible reports not retained in the analytical corpus; Table S4: PRISMA-ScR Checklist; Table S5: Sequential Database Search Strategies; Table S6: Operational definitions and evidence-coding rules; Table S7: Integrated Evidence Charting: 125 Studies. Figure S1: Heat map of reported adsorption interactions across pollutant classes in the mechanistic evidence corpus.

## Abbreviations

The following abbreviations are used in this manuscript:

XPS	X-ray photoelectron spectroscopy
pHpzc	pH at the point of zero charge
PSO	Pseudo-second-order (kinetic model)
RQ	Research question
MMEF	Molecular Mechanistic Evidence Framework
NCI	Noncovalent interactions
RDG	Reduced density gradient
SSA	Specific surface area
CEC	Cation exchange capacity
ECFP	Extended-connectivity fingerprint
DOM	Dissolved organic matter
PDOS	Projected density of states
BET	Brunauer–Emmett–Teller
AI	Artificial intelligence
PPCPs	Pharmaceuticals and personal care products
PFOS	Perfluorooctane sulfonate
2,4-D	2,4-Dichlorophenoxyacetic acid
HOMO	Highest occupied molecular orbital
LUMO	Lowest unoccupied molecular orbital
DOS	Density of states
ESP	Electrostatic potential
Eads	Adsorption energy
CV	Cross-validation
HBD	Hydrogen-bond donor
HBA	Hydrogen-bond acceptor
MW	Molecular weight
log KOW	Logarithm of the octanol–water partition coefficient
log KOC	Logarithm of the organic carbon–water partition coefficient
pKa	Negative logarithm of the acid dissociation constant
PRISMA-ScR	Preferred Reporting Items for Systematic Reviews and Meta-Analyses extension for Scoping Reviews
