# Supplementary material for: Decoding Adsorption Mechanisms in Biomass-Derived Adsorbents: From Experimental Evidence and Molecular Simulation to Explainable and Transferable Prediction
Source: Molecules. 2026 Nov 7;31(21):4018. doi: 10.3390/molecules31214018 (PMC13765007; doi:10.3390/molecules31214018)
Supplement: Supplementary file 1 [file molecules-31-04018-s001.zip › molecules-4647089-supplementary.pdf]

Supplementary material

Decoding Adsorption Mechanisms in Biomass-Derived Adsorbents: From Experimental Evidence and Molecular Simulation to Explainable and Transferable Prediction

Ximena Jaramillo-Fierro[1,*], Julián Hidalgo[2], and Eduardo Valarezo[1]

1 Departamento de Química y Producción, Facultad de Ciencias Exactas y Naturales, Universidad Técnica Particular de Loja, San Cayetano Alto, Loja 110107, Ecuador
2 Carrera de Ingeniería Química, Facultad de Ciencias Exactas y Naturales, Universidad Técnica Particular de Loja, San Cayetano Alto, Loja 110107, Ecuador
* Correspondence: xvjaramillo@utpl.edu.ec ; Tel.: +593-7-3701444

Table S1. Validation strategies reported across the 55 predictive studies

Validation strategy	Studies (n)	Percentage (%)
Train/test split	47	85.5
Cross-validation	34	61.8
Grouped (compound-level) validation	1	1.8
Reported external validation	13	23.6
Reported experimental validation	18	32.7
Validation details requiring further extraction	3	5.5
Total predictive studies	55	100

Note: Validation categories are non-exclusive; therefore, percentages do not sum to 100%. Counts reflect procedures documented in the evidence-charting records. Reported external validation does not necessarily establish out-of-domain generalization, and experimental validation does not necessarily constitute prospective testing. Compound-grouped validation was explicitly documented in one study [16], which also reported leakage control and applicability-domain assessment; across the remaining studies, grouped validation, data-leakage controls, and applicability-domain assessments were not consistently documented and could not be reliably quantified

Table S2. Illustrative application of the Molecular Mechanistic Evidence Framework to representative studies.

Representative study	Molecular depth	Evidence convergence	Environmental realism	Interpretive discipline	Predictive transferability	MMEF interpretation
Liu et al. (2026), tetracycline adsorption on modified cotton-stalk biochar; Table S7, row 5	M3: Solvent-aware DFT, adsorption-related molecular analyses and electronic characterization.	Strong correspondence between spectroscopy and molecular calculations; several interactions are differentiated.	Aqueous solvation is represented computationally, but the full complexity of the composite interface is not necessarily reproduced.	The study recognizes limitations of kinetic-model interpretation. SHAP results are treated as associations rather than independent causal proof.	Internal testing and fivefold cross-validation are reported; independent external transferability was not demonstrated in the study.	Strong mechanistic convergence with limited demonstrated predictive transferability.
Hu et al. (2026), Pb(II) adsorption on functionalized lotus-stem biochar; Table S7, row 6	M2: Explicit adsorbate–surface DFT, including electronic and charge analyses.	Post-adsorption XPS and DFT support Pb binding at electron-rich sites.	The molecular representation does not fully establish performance across environmentally complex aqueous matrices.	Mixed evidence: independent spectroscopy and DFT support coordination, but pseudo-second-order fitting is also used to infer chemisorption.	ML primarily supports material optimization; cross-system transferability is not demonstrated.	Mechanistically plausible conclusion containing a specific unsupported inferential step.
Bouzid et al. (2024), Eriochrome Black T adsorption on modified	M1: Indirect molecular interpretation using a molecular approximation	Zeta potential and pH response support electrostatic contributions;	The DFT representation omits the complete composite interface and	Conflicting inferential strength: experimental evidence supports	A study-specific ANN is reported; independent external validation is	Partial mechanistic support with limited molecular representation and overinterpretation of model fitting.

activated carbon; Table S7, row 8	of the composite surface.	broader mechanistic assignments remain less secure.	does not explicitly represent solvent.	electrostatics, whereas mechanistic interpretations derived from isotherm fitting exceed what those models establish.	not documented.	
Guo et al. (2026), Cu(II) adsorption in the presence of fulvic acid; Table S7, row 11	M2: DFT examines binary and ternary binding configurations.	Adsorption trends, surface characterization and calculated binding sequences converge on a fulvic-acid-mediated mechanism.	Fulvic acid introduces relevant aqueous complexity, although the modeled surface remains simplified.	Mixed evidence: independent data support the proposed binding pathway, but PSO/Langmuir fits are also interpreted as evidence of chemisorption.	The XGBoost model is evaluated within the study domain; independent external validation is not reported (Table S7, row 11).	Strong convergent mechanistic evidence, but with a localized interpretive limitation and restricted predictive generalization.
Zhao et al. (2022), adsorption of organic compounds on biochars; Table S7, row 7	M0: No direct molecular adsorption simulation.	Predictive relationships are supported by the reported dataset analysis but do not independently resolve microscopic adsorption mechanisms.	The literature-derived dataset covers multiple compounds and biochars, but this does not establish behavior in complex natural waters.	Sensitivity analysis is informative but does not establish causal molecular mechanisms.	Fivefold cross-validation is reported; independent validation across previously unseen chemical domains is not demonstrated.	Useful predictive evidence without direct molecular resolution or demonstrated out-of-domain transferability.

Note: The MMEF was applied qualitatively to representative studies selected to illustrate contrasting evidence profiles, including convergent, incomplete, and potentially conflicting interpretations. No numerical scores, weights, or overall quality rankings were assigned. The examples illustrate the framework's interpretive use and do not constitute an independent validation of the MMEF.

Table S3. Thematic coverage of the 363 potentially eligible reports not retained in the analytical corpus

Research question	Analytical domain	Evidence identified in the titles and abstracts of the 363 records	Correspondence with the 125-study synthesis
RQ1	Adsorption mechanisms	Electrostatic interactions, hydrogen bonding, surface complexation, π interactions, pore filling, ion exchange, and redox processes	Mechanistic families already addressed in the synthesis (Table 1).
RQ2	Mechanistic evidence and interpretation	Spectroscopic characterization, surface analysis, kinetic and equilibrium modeling	Evidence types and limitations addressed in the mechanistic-evidence synthesis.
RQ3	Molecular modeling	DFT, molecular dynamics, solvation, speciation, and competitive adsorption	Molecular approaches represented within the evidence archetypes (Table 2).
RQ4	Transferable descriptors	Surface properties, structural characteristics, and physicochemical variables	Descriptor families addressed in the synthesis (Table 4).
RQ5	Predictive modeling and validation	Machine learning, interpretability, and validation-related approaches	Predictive and validation domains discussed in the synthesis (Table 5).

Methodological note: The 363 reports comprised 351 records not advanced to full-text assessment, three non-retrieved reports, one background review, and eight studies excluded after full-text assessment. The 363 records were assessed using their titles, abstracts, and original semantic-screening classifications. The assessment examined whether these records indicated recurrent adsorption mechanisms, molecular-modeling approaches, experimental-evidence categories, descriptor families, or predictive-validation strategies not represented in the 125-study synthesis. The analysis was intended to assess thematic coverage and potential conceptual gaps, not to quantify the prevalence of individual methods or establish full-text eligibility. No additional recurrent mechanistic families or broad methodological domains requiring modification of the analytical framework were identified through this abstract-level assessment. Nevertheless, additional or contradictory findings may be present in the full texts of records not examined at full text.

Table S4. PRISMA-ScR Checklist

Section	Item	PRISMA-ScR reporting item (abridged)	Status	Location / final reporting
TITLE	1	Identify the report as a scoping review.	Reported with qualification	Title page; Abstract; Section 9.1. The manuscript identifies the work as a scoping evidence review and describes the PRISMA-ScR-informed approach.
ABSTRACT	2	Provide a structured summary including background, objectives, eligibility criteria, sources, charting methods, results, and conclusions.	Reported with qualification	Abstract; Sections 9.1–9.3. The Abstract summarizes the objective, approach, 125-study corpus, principal findings, and MMEF; detailed eligibility and charting procedures appear in the Methods.
INTRODUCTION	3	Describe the rationale in the context of existing knowledge and explain why a scoping approach is appropriate.	Reported	Section 1 (Introduction); Section 9.1. The rationale and the suitability of a scoping evidence-mapping approach are explained.
INTRODUCTION	4	Provide explicit questions/objectives and key concepts/context.	Reported	Section 1 (Introduction); Section 9.1. The five research questions (RQ1–RQ5) and review scope are specified.
METHODS	5	State whether a protocol exists and provide registration/access information if available.	Reported	Section 9.1. The manuscript states that no formal review protocol was developed or registered before the review.
METHODS	6	Specify eligibility criteria and rationale.	Reported	Section 9.2. Eligibility criteria, relevant material and pollutant systems, and exclusion boundaries are specified.
METHODS	7	Describe information sources and date of most recent search.	Reported	Section 9.1. Scopus and Web of Science Core Collection are identified; the most recent search was conducted on 31 August 2026.
METHODS	8	Present the full electronic search strategy for at least one database, including limits.	Reported with qualification	Section 9.1; Supplementary Table S5. Search routes, terms, database-specific expressions, and reported limits are documented; reconstructed expressions are identified as such.
METHODS	9	State the process for selecting sources of evidence.	Reported with qualification	Sections 9.2 and 9.4; Table 8; Figure 5; Section 8.7. Screening, relevance prioritization, evidence charting, and analytic closure are described; records not advanced to full-text assessment are not presented as full-text exclusions.
METHODS	10	Describe data-charting methods and whether charting was independent/duplicate.	Reported	Section 9.3. Structured evidence charting, AI-assisted support, author verification, and consensus resolution are described; independent duplicate charting is not claimed.
METHODS	11	List and define variables/data items sought.	Reported	Section 9.3; Supplementary Tables S6 and S7. Charted variables, coding definitions, and study-level evidence are provided.
METHODS	12	If critical appraisal was done, explain rationale, methods, and use.	Reported / adapted	Section 9.3; Supplementary Table S6; Sections 3–6. RQ-specific evidence audits and their interpretive role are described instead of a uniform critical-appraisal score.
METHODS	13	Describe methods for handling and summarizing charted data.	Reported	Sections 9.3–9.4; Sections 2–7. Descriptive synthesis, evidence audits, molecular archetypes, descriptor and validation categories, and MMEF development are explained.
RESULTS	14	Give numbers screened, assessed, and included, with reasons for exclusions, ideally using a flow diagram.	Reported	Section 9.2; Table 8; Figure 5. Identification, deduplication, screening, prioritization, and inclusion counts are reported, together with the number of records excluded at each screening stage and the reasons for the eight full-text exclusions; the 351 records not advanced to full-text assessment are distinguished from formal full-text exclusions.
RESULTS	15	Present characteristics of sources and citations.	Reported	Sections 2–6; Supplementary Table S7. Characteristics and study-level evidence for the 70 mechanistic and 55 predictive studies are presented.
RESULTS	16	If critical appraisal was done, present appraisal results.	Reported	Sections 3–6; Section 7; Supplementary Tables S1, S2, and S6. Claim-level audits, molecular evidence, predictive validation, and framework interpretation are reported.

RESULTS	17	For each source, present relevant charted data that relate to the review questions.	Reported with supplementary support	Supplementary Table S7; Sections 2–6. Charted information for the 125 retained studies is provided alongside cited examples and cross-study synthesis.
RESULTS	18	Summarize and/or present charting results as they relate to review questions/objectives.	Reported	Sections 2–7; Tables 1–7; Figures 1–4. Findings are organized by RQ1–RQ5 and synthesized in the MMEF.
DISCUSSION	19	Summarize main results, including concepts/themes/types of evidence and relevance to review questions.	Reported	Sections 7–8. The principal evidence patterns, MMEF dimensions, knowledge gaps, and research priorities are interpreted.
DISCUSSION	20	Discuss limitations of the scoping review process.	Reported	Section 8.7; Sections 9.2–9.4. The scope and limitations of relevance-prioritized charting and analytic closure are discussed.
DISCUSSION	21	Provide an overall interpretation and implications/next steps.	Reported	Sections 7–8; Section 10 (Conclusions). Implications, research priorities, and next steps are presented; MMEF is not treated as a numerical quality score.
FUNDING	22	Describe funding for the included sources and for the scoping review; describe role of funders.	Review funding reported; source funding not charted	Funding statement. Funding for the review is identified (Universidad Técnica Particular de Loja, project VIN-56); funding of the included studies was not charted or synthesized.

Reporting framework: PRISMA Extension for Scoping Reviews (PRISMA-ScR; Tricco et al., 2018; DOI: 10.7326/M18-0850). Date of the most recent database search: 31 August 2026.

Table S5. Sequential Database Search Strategies

Database	Step / corpus	Purpose	Field	Search string	Records	Sequential role
Scopus	M1	Molecular validation—initial	TITLE-ABS-KEY	TITLE-ABS-KEY(("biochar" OR "hydrochar" OR "biomass-derived adsorbent*" OR "biomass based adsorbent*" OR "biomass-based adsorbent*" OR "bio-based adsorbent*" OR "lignocellulosic adsorbent*" OR "biomass-derived activated carbon*" OR "biomass based activated carbon*" OR "biomass-based activated carbon*") AND adsorp* AND ("density functional theory" OR "DFT calculation*" OR "molecular dynamics" OR "molecular simulation*"))	1,103	Initial molecular search before aqueous restriction.
Scopus	M2	Molecular validation—aqueous restriction	TITLE-ABS-KEY	TITLE-ABS-KEY(("biochar" OR "hydrochar" OR "biomass-derived adsorbent*" OR "biomass based adsorbent*" OR "biomass-based adsorbent*" OR "bio-based adsorbent*" OR "lignocellulosic adsorbent*" OR "biomass-derived activated carbon*" OR "biomass based activated carbon*" OR "biomass-based activated carbon*") AND adsorp* AND ("aqueous" OR "water" OR "wastewater") AND ("density functional theory" OR "DFT calculation*" OR "molecular dynamics" OR "molecular simulation*"))	725	Added aqueous/water/wastewater block.
Scopus	M3	Final molecular corpus (M)	TITLE-ABS-KEY	TITLE-ABS-KEY(("biochar" OR "hydrochar" OR "biomass-derived adsorbent*" OR "biomass based adsorbent*" OR "biomass-based adsorbent*" OR "bio-based adsorbent*" OR "lignocellulosic adsorbent*" OR "biomass-derived activated carbon*" OR "biomass based activated carbon*" OR "biomass-based activated carbon*") AND adsorp* AND ("aqueous" OR "water" OR "wastewater") AND ("pollutant*" OR "contaminant*" OR "dye*" OR "heavy metal*" OR "pharmaceutical*" OR "antibiotic*" OR "pesticide*" OR "PFAS") AND ("density functional theory" OR "DFT calculation*" OR "molecular dynamics" OR "molecular simulation*"))	536	Added pollutant/contaminant block; retained as final Scopus M corpus.

Scopus	P	Final predictive corpus (P)	TITLE-ABS-KEY	TITLE-ABS-KEY(("biochar" OR "hydrochar" OR "biomass-derived adsorbent*" OR "biomass based adsorbent*" OR "biomass-based adsorbent*" OR "bio-based adsorbent*" OR "lignocellulosic adsorbent*" OR "biomass-derived activated carbon*" OR "biomass based activated carbon*" OR "biomass-based activated carbon*") AND adsorp* AND ("aqueous" OR "water" OR "wastewater") AND ("pollutant*" OR "contaminant*" OR "dye*" OR "heavy metal*" OR "pharmaceutical*" OR "antibiotic*" OR "pesticide*" OR "PFAS") AND ("machine learning" OR "explainable artificial intelligence" OR "explainable AI" OR SHAP OR "molecular descriptor*" OR "quantum descriptor*"))	191	Same material, adsorption, aqueous and pollutant blocks; molecular-simulation block replaced by ML/XAI/descriptor block.
Web of Science Core Collection	M	Final molecular corpus (M)	TS (Topic)	TS=(("biochar" OR "hydrochar" OR "biomass-derived adsorbent*" OR "biomass based adsorbent*" OR "biomass-based adsorbent*" OR "bio-based adsorbent*" OR "lignocellulosic adsorbent*" OR "biomass-derived activated carbon*" OR "biomass based activated carbon*" OR "biomass-based activated carbon*") AND adsorp* AND ("aqueous" OR "water" OR "wastewater") AND ("pollutant*" OR "contaminant*" OR "dye*" OR "heavy metal*" OR "pharmaceutical*" OR "antibiotic*" OR "pesticide*" OR "PFAS") AND ("density functional theory" OR "DFT calculation*" OR "molecular dynamics" OR "molecular simulation*"))	100	Equivalent molecular search adapted to WoS Topic syntax.
Web of Science Core Collection	P	Final predictive corpus (P)	TS (Topic)	TS=(("biochar" OR "hydrochar" OR "biomass-derived adsorbent*" OR "biomass based adsorbent*" OR "biomass-based adsorbent*" OR "bio-based adsorbent*" OR "lignocellulosic adsorbent*" OR "biomass-derived activated carbon*" OR "biomass based activated carbon*" OR "biomass-based activated carbon*") AND adsorp* AND ("aqueous" OR "water" OR "wastewater") AND ("pollutant*" OR "contaminant*" OR "dye*" OR "heavy metal*" OR "pharmaceutical*" OR "antibiotic*" OR "pesticide*" OR "PFAS") AND ("machine learning" OR "explainable artificial intelligence" OR "explainable AI" OR SHAP OR "molecular descriptor*" OR "quantum descriptor*"))	81	Equivalent predictive search adapted to WoS Topic syntax.

Reproducibility note. The record counts and conceptual search blocks are preserved in the contemporaneous review documentation. The Scopus searches were developed sequentially (M1 → M2 → M3), followed by the predictive corpus P. Equivalent M and P strategies were then applied in Web of Science. Database-specific field syntax is shown explicitly. The final database totals were 727 Scopus records (536 M + 191 P) and 181 Web of Science records (100 M + 81 P), giving 908 raw records before global deduplication. **Global consolidation.** Within Scopus, M and P yielded 710 unique records after deduplication. Within Web of Science, M and P yielded 168 unique records. Global DOI/title-based deduplication produced 828 unique records from 908 raw records, removing 80 duplicate occurrences. **Search date:** 31 August 2026.

Table S6. Operational definitions and evidence-coding rules.

Analytical domain	Coding category	Operational definition and assignment rule	Interpretation and limitations
RQ1. Adsorption mechanisms	Reported mechanism	An interaction was recorded when explicitly proposed or discussed as an adsorption mechanism in the original study. Multiple mechanisms could be recorded for the same study.	A reported mechanism was not automatically considered experimentally demonstrated.
	Convergently supported mechanism	The proposed mechanism was evaluated against independent experimental, spectroscopic, chemical, or molecular evidence documented in the source study.	Convergent evidence strengthens mechanistic plausibility but does not necessarily establish a unique or dominant mechanism.
	Electrostatic interactions	Charge-dependent interactions involving the adsorbent surface and the adsorbate, considering available surface-charge, pH, and speciation evidence.	pH dependence alone does not uniquely establish electrostatic attraction.
	Hydrogen bonding	Interactions involving hydrogen-bond donors and acceptors associated with the adsorbent and adsorbate.	Functional-group identification alone does not demonstrate hydrogen bonding.
	π interactions	Interactions involving aromatic or conjugated domains of the adsorbent and adsorbate.	Aromatic structures alone do not establish their relative contribution to adsorption.
	Surface complexation/coordination	Specific interactions between adsorbates and surface functional groups or mineral-associated binding sites.	Assignment requires evidence beyond kinetic or equilibrium-model fitting.
	Pore filling/confinement	Adsorption associated with accessible pores, molecular confinement, or size-dependent uptake.	Porosity alone does not establish pore filling as the dominant mechanism.
	van der Waals/noncovalent interactions	Dispersive or other noncovalent contributions identified through the reported experimental or molecular interpretation.	These interactions may coexist with other adsorption mechanisms.
	Hydrophobic interactions	Preferential association of relatively nonpolar adsorbates with hydrophobic adsorbent domains.	Hydrophobicity alone does not demonstrate mechanistic dominance.
	Ion exchange	Exchange of ionic species between solution and adsorbent-associated sites.	Ion exchange should be distinguished from nonspecific electrostatic attraction.
	Precipitation	Formation of a new solid phase associated with pollutant removal.	Precipitation must be distinguished from adsorption onto pre-existing sites.
	Redox processes	Electron-transfer processes involving changes in oxidation state during pollutant removal.	Pollutant removal alone does not demonstrate redox transformation.
RQ3. Molecular-evidence categories	M0 — No direct molecular adsorption simulation	No direct molecular simulation of the adsorbate–adsorbent interaction was identified.	Experimental or predictive evidence may still be available.
	M1 — Indirect molecular descriptors or isolated-molecule calculations	Molecular descriptors or isolated-molecule calculations were reported without explicitly resolving the adsorbate–surface interaction.	Provides indirect molecular interpretation rather than direct interfacial evidence.
	M1b — Statistical-physics or non-atomistic molecular interpretation	Statistical-physics or related non-atomistic approaches were used to interpret adsorption at the molecular level.	Does not explicitly resolve the atomistic adsorbate–surface interface.
	M2 — Explicit adsorbate–surface static DFT	Static DFT calculations explicitly represented an adsorbate interacting with an adsorbent surface or representative surface fragment.	Simplified surfaces and incomplete solvent representation may limit experimental correspondence.

	M3 — Solvated/speciation-aware surface DFT	Explicit adsorbate–surface DFT incorporated solvent treatment and/or chemically relevant speciation.	Static calculations do not necessarily reproduce dynamic aqueous interfacial behavior.
	M4 — Explicit-water MD/dynamic interfacial model	Molecular dynamics or comparable dynamic interfacial modeling explicitly represented water.	Dynamic simulation alone does not establish experimental agreement.
	M5 — Experimentally constrained explicit-water MD	Explicit-water interfacial dynamics were interpreted alongside experimental constraints or corroborating evidence.	Mechanistic interpretation depends on model–system correspondence.
	Cross-cutting molecular attributes	Solvent awareness, speciation treatment, surface realism, and other reported modeling attributes were recorded independently of the primary archetype.	These attributes may occur across different molecular-evidence categories.
RQ5. Predictive-validation strategies	Internal train/test validation	Model performance was evaluated using an internal partition of the available dataset.	Internal predictive accuracy does not establish transferability to independent systems.
	Cross-validation	Model performance was evaluated using *k*-fold or another reported cross-validation procedure.	Random partitioning may preserve dependencies between training and validation observations.
	Grouped validation	Training and testing observations were separated according to a reported grouping variable, such as adsorbent, pollutant, or study source.	Evaluates generalization across the grouping dimension tested.
	External validation	Model performance was evaluated using independently sourced observations not used for model fitting or selection.	Transferability depends on the independence and characteristics of the external dataset.
	Prospective validation	Predictions were evaluated against subsequently generated experimental observations.	Provides evidence of predictive utility within the tested experimental domain.
	Leakage controls	Explicitly reported procedures addressing information leakage during model development or evaluation.	Lack of reporting was not interpreted as proof that leakage occurred.
	Applicability-domain assessment	Explicit evaluation of whether predictions fall within the domain represented by the training data.	High internal accuracy does not establish reliable extrapolation.
RQ2. Claim-level overinterpretation audit	Confirmed overinterpretation	A claim was confirmed when the full text explicitly used kinetic or equilibrium-model fitting to infer chemical versus physical adsorption or another microscopic mechanism that could not be established from the fitted model alone.	Identifies an unsupported inferential step, not necessarily an incorrect overall mechanism.
	False-positive preliminary flag	An initially flagged statement did not meet the confirmation criterion after full-text verification.	Excluded from confirmed overinterpretation counts.
	Independent supporting evidence	Additional experimental, spectroscopic, or molecular evidence supported a mechanism discussed in a study containing a flagged claim.	Independent support did not automatically remove a confirmed local overinterpretation flag.
	Ambiguous or incompletely reported evidence	The available information was insufficient to establish a mechanism, modeling attribute, or validation procedure.	Recorded conservatively rather than inferred from incomplete reporting.

Note: Adsorption mechanisms were coded as non-exclusive categories, whereas molecular-evidence archetypes (M0, M1, M1b, M2, M3, M4, and M5) were mutually exclusive primary classifications, consistent with Table 2 of the main manuscript. Cross-cutting molecular attributes and predictive-validation strategies were recorded separately where reported. Mechanistic claims were distinguished from independently supported interpretations, and kinetic or equilibrium-model fitting alone was not considered sufficient evidence of a microscopic adsorption mechanism. Preliminary overinterpretation flags required full-text verification. Ambiguous or incompletely reported information was treated conservatively. Study-level evidence and coding annotations are provided in Supplementary Table S7.

Table S7. Integrated evidence charts: 125 studies

Nro.	Original class	Citation / DOI	Pollutant	Biomass-derived adsorbent	Primary-corpus status	Full-text evidence class	Experimental adsorption	Surface / spectroscopic evidence	DFT / molecular evidence	DFT model quality	ML / XAI evidence	Mechanism claimed by authors	Mechanism supported by convergent evidence	Experiment–theory convergence (mechanistic) / transferability risk (predictive)	RQ2 manual-audit status	Key limitation	Relevant RQs	Provisional MMEF dimensions
1	A 1	Guo et al. (2026), Chemical Engineering Journal 543, 178300. DOI: 10.1016/j.cej.2026.178300	PFAS (10 PFCA/PFSA species, C4–C12)	Biochar represented computationally by a pristine graphene-like sp2 carbon domain; ML dataset from aqueous biochar adsorption literature	Include — core	Integrated strong	Yes	No / not explicitly charted	Gaussian 16; 4×4 H-passivated graphene model; B3LYP-D3(BJ)/6-311G(d), def2-TZVP single points; PCM water; adsorption energy, FMO, ESP, TDOS/PDOS/OPDOS, ELF, NCI/RDG and charge analysis 1961 samples; six algorithms; Bayesian-optimized CatBoost; 5-fold CV; SHAP + PDP + ALE; DFT-derived molecular descriptors; noise-robustness analysis	M3 — Solvated/speciation-aware surface DFT | Caveat: Simplified/idealized surface	Present within integrated molecular/predictive evidence	Electrostatic; Pore filling/confinement; van der Waals/NCI	Strong evidence for physisorption on pristine graphitic domains, with long-chain affinity driven mainly by cumulative vdW/contact area plus localized electrostatic effects. The same balance is not proven for functionalized/mineral-rich biochars	Moderate / partial convergence	No RQ2 flag identified	Low — authors explicitly distinguish physisorption and acknowledge model simplification Idealized surface limits direct transfer to chemically heterogeneous real biochars; no fully independent external validation of the final ML model	RQ3; RQ4; RQ5	Molecular depth; Evidence convergence; Predictive transferability
2	A 1	Zhang et al. (2025), Chemical Engineering Journal 521, 166840. DOI: 10.1016/j.cej.2025.166840	Cu(II)	Chitosan-derived biochar; urea- and thiourea-doped chitosan microsphere biochars	Include — core	Integrated moderate	Yes	Yes	Local representative molecular structures; ESP and HOMO/LUMO analyses. Main article does not clearly provide functional/basis/solvation details or direct Cu adsorption/binding energies ANN, RF, XGBoost; eight empirical variables; SHAP; XGBoost test R2=0.904; S content and initial Cu concentration dominant	M1 — Indirect molecular descriptors / isolated-model evidence | Caveat: No major realism caveat captured in prior matrix	Present within integrated molecular/predictive evidence	Electrostatic; Surface complexation/coordination; van der Waals/NCI	O/N/S coordination is supported by spectroscopy and performance trends. Specific Cu–S dominance is plausible but less secure because S 2p is weak and DFT does not directly quantify Cu binding in the main text	Moderate / partial convergence	Confirmed overinterpretation	Yes — PSO is used as evidence for chemisorption Weak S 2p signal; incomplete DFT reproducibility in main article; ML is study-specific rather than broadly transferable	RQ1; RQ2; RQ3; RQ4; RQ5	Molecular depth; Evidence convergence; Interpretive discipline; Predictive transferability

3	A 1	Mou et al. (2026), Environmental Research 293, 123669. DOI: 10.1016/j.envres.2025.123669	Morphine	Deep-eutectic-solvent functionalized magnetic shrimp-shell biochar	Include — core	Integrated strong	Yes	Yes	DFT compares hydroxyl-functionalized simplified biochar with pristine biochar; ESP and adsorption energy; MSBC-PG −8.47 kcal/mol vs pristine BC −2.37 kcal/mol SVM, ANN and GBDT; GBDT R2=0.99; SHAP identifies initial concentration, time and oxygen content	M2 — Explicit adsorbate–surface static DFT ∣ Caveat: Simplified/idealized surface	Present within integrated molecular/predictive evidence	Hydrogen bonding; π interactions; Pore filling/confinement	Hydrogen bonding and oxygen-group contribution are strongly convergent across post-characterization, SHAP association and DFT energetics/ESP; pore filling supported by porosity	High convergence	No RQ2 flag identified	Moderate — model-fit language is used, but stronger independent evidence is also present Simplified DFT surface; SHAP association is not causal evidence	RQ1; RQ2; RQ3; RQ4; RQ5	Molecular depth; Evidence convergence; Predictive transferability
4	A 1	Zhang et al. (2026), Frontiers of Environmental Science & Engineering 20(12), 178. DOI: 10.1007/s11783-026-2278-6	Sb(III) / Sb(V)	Biochar literature corpus plus targeted Fe-modified biochars for validation	Include — core	Integrated strong	Yes	No / not explicitly charted	DFT compares Sb(III)/Sb(V) binding to –COO− and –OH groups; favorable adsorption energies including approximately −1.47 and −1.97 eV; speciation-dependent interaction analysis Six algorithms; GBDT test R2=0.934; embedded importance + permutation importance + SHAP + PDP; explicit prediction→hypothesis →DFT→experiment loop	M2 — Explicit adsorbate–surface static DFT ∣ Caveat: Static surface model; solvent treatment not established	Present within integrated molecular/predictive evidence	Not normalized / predictive study	Strong because ML-generated hypotheses are interrogated with DFT and targeted experiments; one of the clearest closed-loop studies	Moderate / partial convergence	No RQ2 flag identified	Low-to-moderate — operational and intrinsic factors are explicitly distinguished Experimental validation still limited in breadth; broader Sb(V)/biochar validation needed for full transferability	RQ3; RQ4; RQ5	Molecular depth; Evidence convergence; Predictive transferability
5	A 1	Liu et al. (2026), Biochar 8:124. DOI: 10.1007/s42773-026-00640-w	Tetracycline	Cotton-stalk biochar + eggshell-derived Ca + β-cyclodextrin	Include — core	Integrated strong	Yes	Yes	Gaussian 09; ωB97X-D/def2-TZVP; SMD water; frequency verification; IGMH and Hirshfeld-surface analyses Multiple models including GBDT; Bayesian optimization + 5-fold CV; SHAP; test R2≈0.9914; code/data availability	M3 — Solvated/speciation-aware surface DFT ∣ Caveat: No major realism caveat captured in prior matrix	Present within integrated molecular/predictive evidence	Hydrogen bonding; Surface complexation/coordination; Ion exchange/bridging; van der Waals/NCI	Strong convergent support from spectroscopy and DFT; individual interaction types are chemically differentiated	High convergence	No RQ2 flag identified	Low — authors explicitly caution that PSO conditionality can produce erroneous comparisons ML is study-specific; composite complexity limits simple attribution to a single mechanism	RQ1; RQ2; RQ3; RQ4; RQ5	Molecular depth; Evidence convergence; Predictive transferability

6	A 1	Hu et al. (2026), Desalination 637, 120474. DOI: 10.1016/j.desal.2026.120474	Pb(II)	Lotus-stem biochar functionalized with Fe3O4 and cucurbit[7]uril	Include — core	Integrated moderate	Yes	Yes	DFT section includes ESP, adsorption-energy comparisons, differential charge density and PDOS; supports Pb binding at electron-rich N/O atoms of CB[7] portals and contribution from the carbon layer 287 synthesis datasets; six models; BP-ANN selected; SHAP/PDP and Pareto optimization. ML chiefly optimizes synthesis/SSA/adsorption capacity rather than providing a transferable adsorption-mechanism model	M2 — Explicit adsorbate–surface static DFT | Caveat: No major realism caveat captured in prior matrix	Present within integrated molecular/predictive evidence	Electrostatic; Surface complexation/coordination	Portal complexation is supported by DFT electronic analyses and post-adsorption XPS. The broad label 'chemisorption' should rely on those data, not PSO fitting	Moderate–high convergence	Confirmed overinterpretation	Yes — PSO $R^2>0.99$ is explicitly interpreted as chemical adsorption; Freundlich fit is also given mechanistic meaning ML task is synthesis optimization, not cross-system mechanism prediction; DFT protocol incompletely reported in main text	RQ1; RQ2; RQ3; RQ5	Molecular depth; Evidence convergence; Environmental realism; Interpretive discipline; Predictive transferability
7	A 1	Zhao et al. (2022), Environmental Research 208, 112694. DOI: 10.1016/j.envres.2022.112694	73 organic compounds	50 literature-derived biochars; polymer resins also modeled	Include — supporting	Predictive supporting	Yes	Yes	No DFT/MD Kriging + pp-LFER; 1750 adsorption data points; 5-fold CV; local sensitivity analysis and Monte Carlo uncertainty analysis; biochar $R^2=0.940$, RMSE=0.037	M0 — No direct molecular adsorption simulation | Caveat: No major realism caveat captured in prior matrix	No / not central to this mechanistic record	Not normalized / predictive study	Useful predictive evidence for RQ4/RQ5 and an important transferability comparator, but it does not independently validate molecular mechanisms	Moderate / partial convergence	No RQ2 flag identified	No major kinetic/isotherm overinterpretation; however, sensitivity should not be read causally Kriging has strong interpolation but poor extension ability; mixed biochar/resin scope; no molecular simulation and no external chemical-space validation	RQ4; RQ5	Molecular depth; Evidence convergence; Predictive transferability
8	A 1	Bouzid et al. (2024), Separation and Purification Technology 351, 128107. DOI: 10.1016/j.seppur.2024.128107	Eriochrome Black T	Nicotiana glauca-derived H3PO4-activated carbon coated with poly(aniline-co-formaldehyde)	Include — supporting	Integrated moderate	Yes	Yes	Gaussian 09/GaussView 6; B3LYP/6-31G(d,p); PAF molecule used as approximation of AC@PAF surface; DFT used mainly for active-site/electrostatic interpretation ANN trained on 117 laboratory points; 17 outliers removed, leaving 100; $R^2\approx0.99$; primarily study-specific process optimization	M1 — Indirect molecular descriptors / isolated-model evidence | Caveat: No major realism caveat captured in prior matrix	Present within integrated molecular/predictive evidence	Electrostatic	Electrostatic attraction is reasonably supported by zeta potential and pH response. The broader physisorption/multilayer interpretation is not independently established by the isotherm fit	Moderate / partial convergence	Confirmed overinterpretation	Yes — paper equates Langmuir with chemisorption and Freundlich with physisorption/multilayer mechanism DFT represents only PAF rather than the composite carbon surface; no solvent;	RQ1; RQ2; RQ3; RQ5	Molecular depth; Evidence convergence; Interpretive discipline; Predictive transferability

																small ANN dataset with outlier deletion and no external validation		
9	A 1	Liu et al. (2026), Bioresource Technology 454, 134715. DOI: 10.1016/j.biortech.2026.134715	Phosphate	Seaweed biochar + magnesite-derived Mg phases + chitosan hydrogel beads	Include — core	Integrated strong	Yes	Yes	Gaussian 09W; M06-2X/6-31+G(d); PCM water; adsorption energies; IGMH + AIM/Multiwfn/VMD. Eads ranks –NH2 (~−44.6 kcal/mol) > –OH (~−37.7) > Mg-coordinated (~−18.6) Six tree models; only 48 observations / 5 features; 3:1 split, Bayesian optimization + 5-fold CV, SHAP; authors explicitly state no independent external dataset	M3 — Solvated/speciation-aware surface DFT ∣ Caveat: No major realism caveat captured in prior matrix	Present within integrated molecular/predictive evidence	Electrostatic; Surface complexation/coordination; Precipitation; van der Waals/NCI	Strong multi-scale convergence. DFT identifies strong local chitosan-group binding while material-scale experiments support complementary Mg-related precipitation/coordination	High convergence	No RQ2 flag identified	Low-to-moderate — mechanistic interpretation is not dependent solely on model fitting Very small ML dataset and no external validation; predictive conclusions should remain within the investigated experimental domain	RQ1; RQ2; RQ3; RQ4; RQ5	Molecular depth; Evidence convergence; Predictive transferability
10	A 1	Seymen et al. (2025), Biomass and Bioenergy 202, 108216. DOI: 10.1016/j.biombioe.2025.108216	Diclofenac	Walnut-shell and pistachio-shell activated carbons	Include — core	Integrated moderate	Yes	Yes	DFT calculates adsorbent–DCF adsorption energies, optimized structures, HOMO/LUMO and Mulliken charges; interpreted alongside H-bonding/electrostatic/π–π interactions ANN predicts DCF removal with reported R2=0.983 and RMSE=0.7119; no XAI reported	M2 — Explicit adsorbate–surface static DFT ∣ Caveat: Static surface model; solvent treatment not established	Present within integrated molecular/predictive evidence	Electrostatic; Hydrogen bonding; π interactions; Pore filling/confinement; van der Waals/NCI	Surface chemistry and DFT support multiple noncovalent interactions and differing affinities, but PFO versus PSO fit cannot establish different molecular mechanisms	Moderate–high convergence	Confirmed overinterpretation	Yes — the identity of the best-fitting kinetic model (PFO vs PSO) is used to infer physical versus chemical adsorption.. Study-specific ANN with no mechanistic XAI; adsorption-model interpretation is stronger than the independent evidence warrants	RQ1; RQ2; RQ3; RQ5	Molecular depth; Evidence convergence; Predictive transferability; Interpretive discipline

11	A1	Guo et al. (2026), Chemical Engineering Journal 543, 178382. DOI: 10.1016/j.cej.2026.178382	Cu(II) in the presence of fulvic acid	Mn-modified Chlorella-derived algal biochar	Include — core	Integrated strong	Yes	Yes	DFT resolves pairwise and ternary binding; supports Cu anchoring at Mn–O/carboxyl sites followed by FA bridging; binding-energy hierarchy used to identify preferred assembly pathway XGBoost test R2=0.941; ML built from the study's adsorption data; FA concentration and pH most influential	M2 — Explicit adsorbate–surface static DFT ∣ Caveat: Static surface model; solvent treatment not established	Present within integrated molecular/ predictive evidence	Surface complexation/coordination; Ion exchange/bridging	Strong because adsorption trends, post-adsorption surface chemistry, DFT binding sequence and ML factor ranking all converge on the concentration-dependent FA mechanism	High convergence	Confirmed overinterpretation	Yes — PSO/Langmuir are described as indicating chemisorption; these fits are not independent proof ML is within-study and not externally validated; local DFT models simplify heterogeneous algal biochar and DOM chemistry	RQ1; RQ2; RQ3; RQ4; RQ5	Molecular depth; Evidence convergence; Environmental realism; Interpretive discipline; Predictive transferability
12	A1	Zheng et al. (2022), Journal of Environmental Chemical Engineering 10, 108381. DOI: 10.1016/j.jece.2022.108381	Ciprofloxacin	Municipal-sludge biochar modified with CoFe layered double hydroxide	Include — core	Integrated moderate	Yes	Yes	DFT compares parallel vs vertical CIP configurations; Eads ~−0.0597 eV (SBC parallel) and ~−0.1209 eV (CoFe-LDH@SBC parallel); differential charge density used to assess interaction ANN test/train/validation R2≈0.9446/0.9882/0.9653; MSE≈0.004; no model-agnostic XAI	M2 — Explicit adsorbate–surface static DFT ∣ Caveat: Static surface model; solvent treatment not established	Present within integrated molecular/ predictive evidence	Electrostatic; Hydrogen bonding; π interactions; Surface complexation/coordination	Parallel orientation and enhanced interaction after LDH modification are supported by DFT and experiment. However, 'chemisorption' inferred from Elovich/kinetic fitting is stronger than the molecular-energy evidence alone	Moderate–high convergence	Confirmed overinterpretation	Yes — Elovich/kinetic fit is used to infer chemisorption and monolayer behavior; ANN is claimed usable under any conditions beyond its demonstrated domain Weak DFT adsorption energies relative to broad chemisorption claim; ANN generalization is overextended beyond the experimental domain	RQ1; RQ2; RQ3; RQ5	Molecular depth; Evidence convergence; Interpretive discipline; Predictive transferability
13	A2a	Chen et al. (2023), Environmental Research 219, 115128 ∣ DOI: https://doi.org/10.1016/j.envres.2022.115128	TCEP	Shrimp-shell biochar	Include — core	Strong	Yes	No / not explicitly charted	DFT binding-energy analysis on simplified biochar motifs; H-bond, vdW, Cl–H and π–H interactions; oxygenated groups ranked as favorable	M2 — Explicit adsorbate–surface static DFT ∣ Caveat: Simplified/ idealized surface	No / not central to this mechanistic record	Hydrogen bonding; van der Waals/NCI	Well supported by pore evidence + adsorption behavior + direct DFT binding calculations	Moderate / partial convergence	No RQ2 flag identified	Low	RQ1; RQ3; RQ4	Molecular depth; Evidence convergence; Predictive transferability
14	A2a	Chen et al. (2023), Science of the Total Environment 862, 160860 ∣ DOI: https://doi.org/10.1016/j.scitotenv.2022.160860	Tetracycline	La-modified orange-peel biochar	Include — core	Strong	Yes	Yes	DFT used with XPS to identify La/oxygen-containing binding motifs and explain	M2 — Explicit adsorbate–surface static DFT	No / not central to this mechanistic record	Not normalized / predictive study	Mechanism is supported by XPS + material trends + theory; kinetic	High convergence	Confirmed overinterpretation	Yes — the better Freundlich fit is interpreted as multilayer	RQ1; RQ2; RQ3; RQ4	Molecular depth; Evidence convergence;

									pyrolysis-temperature effects	| Caveat: Static surface model; solvent treatment not established			fit should not be treated as proof			adsorption and the Temkin fit as chemisorption; the PSO discussion is descriptive		Predictive transferability; Interpretative discipline
15	A 2a	Cao et al. (2026), Ecotoxicology and Environmental Safety 311, 119881 | DOI: https://doi.org/10.1016/j.ecoenv.2026.119881	Crude oil + Cd(II)	Bagasse biochar supported with iron species and carbon nanotubes	Include — core	Moderate	Yes	No / not explicitly charted	DFT on Fe_3O_4–biochar motif; binding energy and HOMO–LUMO gap used to infer enhanced electronic activity	M2 — Explicit adsorbate–surface static DFT | Caveat: Static surface model; solvent treatment not established	No / not central to this mechanistic record	Electrostatic; π interactions; Pore filling/confinement; Surface complexation/coordination	General interaction picture is supported; the exact contribution of CNT vs Fe sites is less resolved	Moderate / partial convergence	Confirmed overinterpretation	Yes — the Freundlich fit is equated with heterogeneous multilayer adsorption.	RQ1; RQ2; RQ3	Molecular depth; Evidence convergence; Interpretative discipline
16	A 2a	Tabassam et al. (2022), Materials Chemistry and Physics 288, 126327 | DOI: https://doi.org/10.1016/j.matchemphys.2022.126327	Methylene blue	Cinnamic-acid-modified rice-husk biochar	Include — supporting	Moderate / supporting	Yes	Yes	DFT mainly uses molecular electrostatic potential/reactive-site analysis rather than a deeply realistic adsorbent–adsorbate surface model	M1 — Indirect molecular descriptors / isolated-model evidence | Caveat: No major realism caveat captured in prior matrix	No / not central to this mechanistic record	Electrostatic; Hydrogen bonding; π interactions; Ion exchange/bridging	Surface chemistry supports electrostatics/functional-group involvement; molecular validation is comparatively shallow	Moderate / partial convergence	Confirmed overinterpretation	Yes — PSO→chemical adsorption and Langmuir→monolayer mechanism	RQ1; RQ2; RQ3	Molecular depth; Evidence convergence; Interpretive discipline
17	A 2a	Zhao et al. (2021), Environmental Pollution 291, 118244 | DOI: https://doi.org/10.1016/j.envpol.2021.118244	Cr(VI)	Urea-derived N-functionalized poplar-leaf biochar	Include — core	Strong	Yes	No / not explicitly charted	Protonation energies, protonation-site prediction and adsorption energies; H-bond interactions; strongest reported adsorption energy about −5.01 kcal/mol	M2 — Explicit adsorbate–surface static DFT | Caveat: Static surface model; solvent treatment not established	No / not central to this mechanistic record	Electrostatic; Hydrogen bonding; Redox/transformation	Excellent causal chain from synthetic modification → protonation behavior → pH response → DFT	Moderate / partial convergence	No RQ2 flag identified	Low	RQ1; RQ3; RQ4	Molecular depth; Evidence convergence; Predictive transferability
18	A 2a	Fang et al. (2022), Environmental Pollution 310, 119819 | DOI: https://doi.org/10.1016/j.envpol.2022.119819	Bisphenol S	Oriental plane tree biochar	Include — core	Strong	Yes	Yes	DFT compares π–π and H-bonding interactions and identifies stronger π–π contribution at high-temperature biochar	M2 — Explicit adsorbate–surface static DFT | Caveat: Static surface model;	No / not central to this mechanistic record	Hydrogen bonding; π interactions	One of the clearest examples of convergent mechanism discrimination	High convergence	Confirmed overinterpretation	Yes — PSO/Langmuir fits are interpreted as chemisorption/ monolayer adsorption; however, the final π–	RQ1; RQ2; RQ3; RQ4	Molecular depth; Evidence convergence; Interpretive discipline; Predictive

										solvent treatment not established						π/hydrogen-bond assignment is independently supported by DFT, FTIR/Raman, and thermodynamic evidence.		transferability
19	A 2a	Ji et al. (2025), Colloids and Surfaces A 715, 136615 ∣ DOI: https://doi.org/10.1016/j.colsurfa.2025.136615	Methylene blue	KOH-activated wheat-straw porous biochar	Include — core	Strong	Yes	Yes	DFT identifies oxygen-functionalized sites, π–π interactions, electron transfer and confinement effects	M2 — Explicit adsorbate–surface static DFT ∣ Caveat: Static surface model; solvent treatment not established	No / not central to this mechanistic record	π interactions; Pore filling/confinement	Strong overall, especially where pore topology and DFT electronic interactions converge	High convergence	Confirmed overinterpretation	Yes — PSO and Langmuir are described as evidence of chemisorption/ monolayer behavior	RQ1; RQ2; RQ3; RQ4	Molecular depth; Evidence convergence; Interpretive discipline; Predictive transferability
20	A 2a	Du et al. (2026), Colloids and Surfaces A 735, 139478 ∣ DOI: https://doi.org/10.1016/j.colsurfa.2026.139478	Cu(II) + ciprofloxacin	K2FeO4-activated cotton-husk magnetic biochar	Include — core	Strong	Yes	Yes	DFT constructs Cu–CIP coordination configurations and adsorbent binding models; HOMO–LUMO, optimized geometry and adsorption energies support Cu-mediated bridging	M2 — Explicit adsorbate–surface static DFT ∣ Caveat: Static surface model; solvent treatment not established	No / not central to this mechanistic record	Surface complexation/coordination; Ion exchange/bridging	Strong experiment–XPS/FTIR–DFT agreement	High convergence	No RQ2 flag identified	Low-to-moderate	RQ1; RQ3; RQ4	Molecular depth; Evidence convergence; Predictive transferability
21	A 2a	Yan et al. (2026), differential NOX/TY adsorption study ∣ DOI: 10.1016/j.psep.2026.109148	Norfloxacin + tylosin	Potassium-formate-activated peanut-meal biochar	Include — core	Strong	Yes	Yes	DFT compares interaction energies/speciation; neutral NOX reported with strongest adsorption energy (~−228.28 kJ/mol)	M3 — Solvated/speciation-aware surface DFT ∣ Caveat: No major realism caveat captured in prior matrix	No / not central to this mechanistic record	Hydrogen bonding; π interactions; Pore filling/confinement	Strong because pore-size matching and molecular dimensions are tested alongside DFT interaction energies	High convergence	No RQ2 flag identified	Moderate — kinetic/isotherm labels are present but stronger evidence exists	RQ1; RQ3; RQ4	Molecular depth; Evidence convergence; Predictive transferability
22	A 2a	Liu et al. (2025), Separation and Purification Technology, 135077 ∣ DOI: https://doi.org/10.1016/j.seppur.2025.135077	Bisphenol A	N-doped corn-kernel/corn-cob waste biochar	Include — core	Strong	Yes	No / not explicitly charted	DFT and comparative experiments identify oxidized-N and graphitic-N sites; electron-transfer enhancement of π–π EDA interactions	M2 — Explicit adsorbate–surface static DFT ∣ Caveat: Static surface	No / not central to this mechanistic record	Electrostatic; Hydrogen bonding; π interactions	Strong convergent site-specific evidence	Moderate / partial convergence	Confirmed overinterpretation	Yes — Langmuir + PSO are described as monolayer chemisorption	RQ1; RQ2; RQ3; RQ4	Molecular depth; Evidence convergence; Interpretive discipline;

										model; solvent treatment not established								Predictive transferability
23	A 2a	Maung et al. (2026), acid-mine-drainage NTA-biochar study	DOI: 10.1016/j.seppur.2026.138070	Mn(II), Co(II), Cu(II)	NTA-modified banana-peel biochar	Include — core	Strong	Yes	Yes	DMol3 DFT; adsorption energies, DOS, TDA, NCI, simulated UV/IR/Raman; Bio-COOH-NTA-Cu most stable (~−113.8 kcal/mol)	M2 — Explicit adsorbate–surface static DFT	Caveat: Static surface model; solvent treatment not established	No / not central to this mechanistic record	van der Waals/NCI	Very strong because real-matrix selectivity, XPS and multiple quantum descriptors converge	High convergence	No RQ2 flag identified	Low	RQ1; RQ3; RQ4	Molecular depth; Evidence convergence; Predictive transferability
24	A 2a	Li et al. (2026), Separation and Purification Technology 410, 139337	DOI: 10.1016/j.seppur.2026.139337	Cr(VI)	Sulfur-modified bamboo biochar	Include — core	Strong	Yes	Yes	DFT supports $HCrO_4^-$ enrichment on S/O sites, charge redistribution and subsequent Cr(III)–O/S fixation	M2 — Explicit adsorbate–surface static DFT	Caveat: Static surface model; solvent treatment not established	No / not central to this mechanistic record	Surface complexation/coordination; Redox/transformation	Strong; authors explicitly avoid inferring the entire pathway from XPS alone	High convergence	Confirmed overinterpretation	Yes — PSO adherence is interpreted as rate-limiting chemisorption and the Langmuir fit as monolayer homogeneous coverage; the Cr(VI) pathway is independently supported by XPS and DFT.	RQ1; RQ2; RQ3	Molecular depth; Evidence convergence; Predictive transferability
25	A 2a	Xu et al. (2025), Chemical Engineering Journal 523, 168752	DOI: https://doi.org/10.1016/j.cej.2025.168752	Norfloxacin	Corn-straw magnetic biochar + humic acid + MOF-303	Include — core	Strong	Yes	No / not explicitly charted	DFT tests ultramicropore confinement and Al^{3+} coordination; energetically favorable coordination and stronger confinement interactions	M2 — Explicit adsorbate–surface static DFT	Caveat: No major realism caveat captured in prior matrix	No / not central to this mechanistic record	π interactions; Pore filling/confinement; Surface complexation/coordination	Strong, especially for coordination + size-matching hypothesis	Moderate / partial convergence	No RQ2 flag identified	Low	RQ1; RQ3; RQ4	Molecular depth; Evidence convergence; Environmental realism; Predictive transferability
26	A 2a	Xin et al. (2026), Fe/Mg-N-doped biochar study	DOI: 10.1016/j.cej.2026.177573	PFOA/PFOS/PFBA/PFBS + Cr(VI)	Fe/Mg co-modified N-doped biomass biochar	Include — core	Strong	Yes	Yes	DFT: PFAS adsorption energy changes from +0.43 eV on pristine biochar to ~−1.24 eV on Fe/Mg-NBC; co-adsorption configurations resolve complementary metal roles	M2 — Explicit adsorbate–surface static DFT	Caveat: No major realism caveat captured in prior matrix	No / not central to this mechanistic record	Electrostatic; Pore filling/confinement; Hydrophobic; Surface complexation/coordination; Redox/transformation	Strong if PFAS adsorption and Cr transformation are discussed separately	High convergence	No RQ2 flag identified	Moderate	RQ1; RQ3; RQ4	Molecular depth; Evidence convergence; Predictive transferability

27	A 2a	Ren et al. (2025), DFT-validated metronidazole adsorption study | DOI: 10.1016/j.dwt.2025.101414	Metronidazole	CO_2-activated almond-shell biochar with polymer/humate modifications	Include — core	Moderate	Yes	Yes	DFT supports π–π and H-bond contributions and differences among modified carbons	M2 — Explicit adsorbate–surface static DFT | Caveat: Static surface model; solvent treatment not established	No / not central to this mechanistic record	Hydrogen bonding; π interactions	Supported overall, but the label 'chemical adsorption' should not come from PSO alone	Moderate–high convergence	Confirmed overinterpretation	Yes — PSO explicitly used to assert chemical adsorption	RQ1; RQ2; RQ3	Molecular depth; Evidence convergence; Interpretive discipline
28	A 2a	Yang et al. (2025), Journal of Environmental Chemical Engineering, 115279 | DOI: https://doi.org/10.1016/j.jece.2024.115279	Pb(II)	Zn/Fe-oxide-modified walnut-shell biochar	Include — core	Strong	Yes	No / not explicitly charted	Periodic/slab DFT with PAW, 450 eV cutoff and Grimme D3; ZnO/FeOx surfaces, Pb adsorption energy, IRI/Multiwfn weak-interaction analysis	M2 — Explicit adsorbate–surface static DFT | Caveat: Static surface model; solvent treatment not established	No / not central to this mechanistic record	π interactions; Surface complexation/coordination; Precipitation; van der Waals/NCI	Strong multi-technique support	Moderate / partial convergence	Confirmed overinterpretation	Yes — PSO superiority is taken to confirm chemical adsorption, and Freundlich/Langmuir fits are interpreted as multilayer/monolayer mechanisms.	RQ1; RQ2; RQ3	Molecular depth; Evidence convergence; Predictive transferability
29	A 2a	Maung et al. (2026), Journal of Environmental Chemical Engineering 14, 120814 | DOI: 10.1016/j.jece.2025.120814	Cu(II), Co(II), Mn(II)	NTA-modified banana-peel biochar	Include — core	Moderate	Yes	No / not explicitly charted	DFT evaluates electrophilicity and charge transfer for Biochar-COOH-NTA-metal complexes	M2 — Explicit adsorbate–surface static DFT | Caveat: Static surface model; solvent treatment not established	No / not central to this mechanistic record	Surface complexation/coordination	Reasonably supported; should be compared carefully with the later AMD study from the same group	Moderate / partial convergence	Confirmed overinterpretation	Yes — PSO fitting is interpreted as chemical control, and Langmuir/D-R fits are used to assign monolayer and chemisorption/physisorption behavior.	RQ1; RQ2; RQ3	Molecular depth; Evidence convergence: Predictive transferability
30	A 2a	He et al. (2026), pinecone biochar metal-oxide comparison | DOI: 10.1016/j.jece.2026.121391	Ciprofloxacin	KOH-activated pinecone biochar doped with Fe, Mg or Mn oxides	Include — core	Strong	Yes	Yes	DFT reports Fe–O_CIP coordination distance ~1.87 Å, charge redistribution and adsorption energy ~−4.52 eV for Fe-PC	M2 — Explicit adsorbate–surface static DFT | Caveat: Static surface model; solvent treatment not established	No / not central to this mechanistic record	Electrostatic; π interactions; Pore filling/confinement; Surface complexation/coordination	Strong because metal-series comparison, spectroscopy and DFT all identify Fe sites	High convergence	Confirmed overinterpretation	Yes — PSO-fitted capacities are interpreted as chemisorption and the Langmuir fit as monolayer adsorption on uniform sites.	RQ1; RQ2; RQ3; RQ4	Molecular depth; Evidence convergence; Predictive transferability
31	A 2a	Florez et al. — molecular benchmark study | DOI: 10.1016/j.mtcomm.2024.108647	Model adsorbates / functionalized carbon surfaces	Functionalized carbon/biochar-like	Include — supporting	Strong molecular / limited experimental	Yes	Yes	DFT explores functional groups, surface charge, implicit solvation, adsorption energies, ΔG and charge transfer	M3 — Solvated/speciation-aware surface	No / not central to this mechanistic record	Not normalized / predictive study	Valuable as a molecular-method benchmark rather than a	High convergence	No RQ2 flag identified	Low	RQ3; RQ4	Molecular depth; Evidence convergence;

				surface models						DFT ǀ Caveat: No major realism caveat captured in prior matrix			stand-alone experiment–theory convergence exemplar					Predictive transferability
32	A 2a	Al-Haiqi et al. (2026), ACS Omega 11, 39943–39959 ǀ DOI: 10.1021/acsomega.6c01218	Paracetamol	Fe/Mg-incorporated N-doped coconut-shell biochar	Include — core	Strong	Yes	Yes	DFT shows stronger adsorption energy after Fe/Mg co-doping; supports metal complexation, cation–π, H-bonding and π–π stacking	M2 — Explicit adsorbate–surface static DFT ǀ Caveat: No major realism caveat captured in prior matrix	No / not central to this mechanistic record	Hydrogen bonding; π interactions; Surface complexation/coordination	Strong convergence between surface characterization and DFT-enhanced interaction energy	High convergence	No RQ2 flag identified	Moderate — PSO/Langmuir fits are prominent	RQ1; RQ2; RQ3	Molecular depth; Evidence convergence
33	A 2a	Joshi & Ragupathy (2025), RSC Advances 15, 34609–34634 ǀ DOI: 10.1039/d5ra03863d	Pb(II), Cd(II), Cr(VI)	Waste-derived carbon adsorbents including Prosopis biochar and banana-stem char	Include — core	Strong	Yes	Yes	DFT + QTAIM; metal-specific functional-group binding energies and orbital-overlap/charge-transfer interpretation	M2 — Explicit adsorbate–surface static DFT ǀ Caveat: Static surface model; solvent treatment not established	No / not central to this mechanistic record	Electrostatic	Strong comparative mechanistic discrimination across metals	High convergence	No RQ2 flag identified	Low-to-moderate	RQ1; RQ3; RQ4	Molecular depth; Evidence convergence; Predictive transferability
34	A 2a	Zhang et al. (2025), Biochar 7:38. DOI: 10.1007/s42773-024-00420-4	Tetracycline	Wheat-straw biochar/Fe3O4/SnO2 nanocomposites	Include — supporting/ contextual	Mixed adsorption – photocatalysis	Yes	Yes	DFT primarily verifies electronic/photocatalytic charge-transfer mechanism rather than direct tetracycline–biochar adsorption energetics	M0 — No direct molecular adsorption simulation ǀ Caveat: No major realism caveat captured in prior matrix	No / not central to this mechanistic record	Redox/transformation	Use only to illustrate boundary between adsorption and transformation; do not treat as a core molecular adsorption validation	Moderate / partial convergence	No RQ2 flag identified	Moderate	RQ1/ RQ2 contextual	Molecular depth; Evidence convergence
35	A 2a	Imtiyaz & Rasool (2026), International Journal of Environmental Research 20:2. DOI: 10.1007/s41742-025-00965-7	Malachite green	Apple-pomace biochar produced via ZSM-5 catalytic fast pyrolysis	Include — supporting	Weak molecular validation	Yes	No / not explicitly charted	DFT is performed on isolated malachite-green molecular properties; no explicit biochar surface, adsorption complex, solvent-corrected binding energy or adsorbate docking	M1 — Indirect molecular descriptors / isolated-model evidence ǀ Caveat: Indirect/no explicit adsorbent surface	No / not central to this mechanistic record	Electrostatic	Experimental adsorption is in scope, but DFT does not directly validate MG–biochar binding; ideal RQ2/RQ3 counterexample	Limited / contextual	Confirmed overinterpretation	Yes — the conclusions infer chemisorption from PSO kinetics, and isolated-molecule DFT is treated as mechanistic confirmation.	RQ2; RQ3	Molecular depth; Evidence convergence; Predictive transferability

36	A2b	Huang et al. (2025), Journal of Cleaner Production 537, 147172. DOI 10.1016/j.jclepro.2025.147172	As(III) + tetracycline	Ferrihydrite confined in B-doped biochar	Include — supporting/contextual	Mixed adsorption–oxidation	Yes	Yes	DFT supports inner-sphere As binding/co-precipitation and separately models O2 adsorption, work functions and defect-driven ROS generation.	M2 — Explicit adsorbate–surface static DFT | Caveat: Static surface model; solvent treatment not established	No / not central to this mechanistic record	Surface complexation/coordination; Precipitation; Redox/transformation	As adsorption mechanism is relevant; TC pathway is not adsorption evidence.	Moderate–high convergence	No RQ2 flag identified	Low	RQ1; RQ3	Molecular depth; Evidence convergence
37	A2b	Zhu et al. (2026), Bioresource Technology 454, 134729. DOI 10.1016/j.biortech.2026.134729	Bisphenol A	O-coordinated Fe single-atom biochar	Include — core	Strong	Yes	No / not explicitly charted	Five FeOn single-atom sites on graphene support are modeled; DFT predicts O-coordination dependence, π-complexation and d–π* back-donation, then guides synthesis.	M2 — Explicit adsorbate–surface static DFT | Caveat: Static surface model; solvent treatment not established	No / not central to this mechanistic record	π interactions; Pore filling/confinement; Surface complexation/coordination	Very strong evidence because the specific coordination motif predicted by DFT is synthesized and experimentally verified.	Moderate / partial convergence	No RQ2 flag identified	Low	RQ1; RQ3; RQ4	Molecular depth; Evidence convergence; Predictive transferability
38	A2b	Zhang et al. (2026), Journal of Molecular Graphics and Modelling 144, 109276. DOI 10.1016/j.jmgm.2026.109276	Sulfamethoxazole	Biochar pore-structure models + experimental biochar	Include — core	Strong MD	Yes	No / not explicitly charted	Explicit-water MD across mesopore, micropore, hierarchical-pore and amorphous-carbon models; SPC/E water; diffusion coefficients, NCI and energy decomposition; vdW ~59%.	M4 — Explicit-water MD / dynamic interfacial model | Caveat: No major realism caveat captured in prior matrix	No / not central to this mechanistic record	Hydrogen bonding; π interactions; Pore filling/confinement; van der Waals/NCI	Excellent experiment–MD convergence and a high-value RQ3/RQ4 study.	Moderate / partial convergence	No RQ2 flag identified	Moderate — PSO/Langmuir are reported, but they are not the main mechanistic proof.	RQ1; RQ3; RQ4	Molecular depth; Evidence convergence; Predictive transferability
39	A2b	Ji et al. (2024), Separation and Purification Technology 330, 125235. DOI: 10.1016/j.seppur.2023.125235	Imidacloprid	Fe/Mg co-modified water-hyacinth biochar	Include — core	Strong-to-moderate	Yes	Yes	Gaussian 16/B3LYP with aqueous solvation model; adsorption energies used to rank metal-oxide complexation and pollutant affinity. Authors note first hydration shells are not explicitly included.	M3 — Solvated/speciation-aware surface DFT | Caveat: No major realism caveat captured in prior matrix	No / not central to this mechanistic record	Electrostatic; Hydrogen bonding; π interactions; Pore filling/confinement; Surface complexation/coordination	Surface complexation is supported by spectroscopy, DFT energetics and fixed-bed performance.	High convergence	No RQ2 flag identified	Moderate — PSO/Sips are used descriptively; avoid reading them as bond-type proof.	RQ1; RQ3; RQ4	Molecular depth; Evidence convergence; Predictive transferability

40	A2b	Cheng et al. (2026), Separation and Purification Technology 380, 135206. DOI 10.1016/j.seppur.2025.135206	Phosphate + Cr(VI)	Sludge-biochar@ZnAl-LDO composite	Include — core	Strong	Yes	No / not explicitly charted	DFT optimizes adsorption geometries and electronic structure; FMO/PDOS/charge flow; adsorption energies to −7.902 eV for H2PO4− and −4.462 eV for HCrO4−.	M2 — Explicit adsorbate–surface static DFT | Caveat: Static surface model; solvent treatment not established	No / not central to this mechanistic record	Electrostatic; Hydrogen bonding; Surface complexation/coordination; Redox/transformation	Strong if phosphate adsorption and Cr adsorption–reduction are explicitly separated.	Moderate / partial convergence	Confirmed overinterpretation	Yes — Elovich/Langmuir are described as chemisorption/monolayer mechanism.	RQ1; RQ2; RQ3	Molecular depth; Evidence convergence; Interpretive discipline
41	A2b	Wu et al. (2026), Separation and Purification Technology 398, 138054. DOI 10.1016/j.seppur.2026.138054	PFOA	Aminosilanized bamboo-derived activated biochar	Include — core	Strong	Yes	No / not explicitly charted	Periodic VASP/PBE, 450 eV cutoff, 20 Å vacuum, DFT-D3 dispersion; adsorption energies distinguish end-group orientation (−1.16 vs −0.42 eV).	M2 — Explicit adsorbate–surface static DFT | Caveat: No major realism caveat captured in prior matrix	No / not central to this mechanistic record	Electrostatic	Strong experiment–DFT–real-water convergence.	Moderate / partial convergence	No RQ2 flag identified	Low	RQ1; RQ3; RQ4	Molecular depth; Evidence convergence; Environmental realism; Predictive transferability
42	A2b	Di et al. (2026), Separation and Purification Technology 402, 138706	Bisphenol S	Al-doped biochar	Include — core	Strong	Yes	No / not explicitly charted	DFT quantifies charge transfer (0.0108 to −0.0278 |e|) and adsorption energy (−0.7007 to −1.2476 eV); analyzes BPS ring dihedral angle and Al–O role.	M2 — Explicit adsorbate–surface static DFT | Caveat: No major realism caveat captured in prior matrix	No / not central to this mechanistic record	π interactions	Very strong mechanism discrimination linking doping, electronic structure and macroscopic adsorption.	Moderate / partial convergence	No RQ2 flag identified	Low	RQ1; RQ3; RQ4	Molecular depth; Evidence convergence; Environmental realism; Predictive transferability
43	A2b	Ma et al. (2024), Chemical Engineering Journal 492, 152189. DOI 10.1016/j.cej.2024.152189	Tetracycline hydrochloride	N,S co-modified chitosan-derived biochar	Include — core	Strong	Yes	No / not explicitly charted	DFT compares heteroatom/oxygen sites on graphene and identifies pyrrolic-N and thiophene-S as high-affinity chemisorption sites.	M2 — Explicit adsorbate–surface static DFT | Caveat: No major realism caveat captured in prior matrix	No / not central to this mechanistic record	Electrostatic; Hydrogen bonding; π interactions; Pore filling/confinement	Strong because pore-size dependence and site chemistry are independently supported.	Moderate / partial convergence	Confirmed overinterpretation	Yes — the PSO fit is interpreted as monolayer chemical bonding, Elovich and Temkin fits as evidence of chemisorption, the Langmuir fit as monolayer adsorption on homogeneous sites, and Freundlich 1/n and D-R energies as physical adsorption; the	RQ1; RQ2; RQ3; RQ4	Molecular depth; Evidence convergence; Predictive transferability

																dominant hydrogen-bond and π–π assignment is independently supported by FTIR, XPS, and DFT.		
44	A2b	Peng et al. (2025), Journal of Environmental Chemical Engineering 13, 117186. DOI 10.1016/j.jece.2025.117186	Tetracycline	Mn/N co-doped biochar prepared with metal-based ionic liquid	Include — core	Moderate-to-strong	Yes	No / not explicitly charted	DFT + Multiwfn/VMD; ESP, energy-gap and adsorption-energy changes used to explain Mn/N electronic effects and surface complexation.	M2 — Explicit adsorbate–surface static DFT ∣ Caveat: No major realism caveat captured in prior matrix	No / not central to this mechanistic record	Hydrogen bonding; π interactions; Pore filling/confinement; Surface complexation/coordination	Mechanism is supported, though some electronic descriptors are less direct than explicit bond-topology analyses.	Moderate / partial convergence	Confirmed overinterpretation	Yes — PSO/Langmuir are described as monolayer behavior.	RQ1; RQ2; RQ3	Molecular depth; Evidence convergence; Environmental realism; Interpretive discipline
45	A2b	Bai et al. (2021), Nanomaterials 11, 3255. DOI 10.3390/nano11123255	Pb(II) + Cu(II)	Humulus scandens-derived molten-salt biochar	Include — core	Moderate	Yes	No / not explicitly charted	DFT indicates oxygen groups increase metal binding energies; ion exchange is also invoked.	M2 — Explicit adsorbate–surface static DFT ∣ Caveat: No major realism caveat captured in prior matrix	No / not central to this mechanistic record	Ion exchange/bridging	Supported overall, but the molecular mechanism should rely on DFT + chemistry rather than Langmuir.	Moderate / partial convergence	Confirmed overinterpretation	Yes — the Langmuir fit is presented as evidence that chemical adsorption governs removal.	RQ1; RQ2; RQ3	Molecular depth; Evidence convergence; Environmental realism; Predictive transferability
46	A2b	Gong et al. (2026), Biochar 8:120. DOI 10.1007/s42773-026-00642-8	PFOS	β-Ga2O3-functionalized biochar	Include — supporting/contextual	Adsorption + photodestruction	Yes	No / not explicitly charted	VASP/PBE first-principles DFT mainly establishes electron transfer from biochar to Ga2O3 and photocatalytic charge separation rather than direct PFOS binding.	M0 — No direct molecular adsorption simulation ∣ Caveat: No major realism caveat captured in prior matrix	No / not central to this mechanistic record	Electrostatic; Hydrogen bonding; Pore filling/confinement; Hydrophobic; van der Waals/NCI	Retain as a boundary case for 'concentrate-and-destroy', not as core molecular adsorption validation.	Limited / contextual	No RQ2 flag identified	Low	RQ1 contextual; RQ3 methodological	Molecular depth; Evidence convergence
47	A2c	Unravelling 2,4-D – biochar interactions by molecular dynamics: adsorption modes and surface functionalities ∣ DOI: 10.1016/j.biombioe.2026.109080	2,4-D	Experimentally constrained softwood biochar models (400/600/800 °C)	Include — core	Exceptional MD	Yes	No / not explicitly charted	GROMACS 2022; OPLS-AA; SPC water; 24 systems; 50+ ns NPT runs; realistic surfaces matched to H/C, O/C, aromaticity, density and functionality	M5 — Experimentally constrained explicit-water MD ∣ Caveat: No major realism caveat captured in prior matrix	No / not central to this mechanistic record	Hydrogen bonding; π interactions; Surface complexation/coordination; Ion exchange/bridging	Low-temperature biochar gains cooperative polar interactions; higher-temperature biochar relies more on π interactions and cation bridging	Moderate / partial convergence	No RQ2 flag identified	Simulation concentrations are deliberately much higher than environmental concentrations; authors explain this finite-size sampling compromise.	RQ1, RQ2, RQ3, RQ4	Molecular depth; Evidence convergence; Predictive transferability

48	A 2c	Ammonium recovery from wastewater by biochar with different N/O-doped groups and hierarchical pores: Synergistic enhancement mechanisms for strong negative electrostatic potential and micropore filling ǀ DOI: 10.1016/j.jenvman.2025.125776	$NH4^+$	N/O-doped, hierarchically porous rapeseed-straw biochar	Include — core	Very strong DFT+MD	Yes	Yes	Gaussian16 M06-2X/6-311G(d) geometry, M06-2X/6-311G(2d,p) single points, SMD water; ESP, IGM, electron-density difference + GROMACS MD	M4 — Explicit-water MD / dynamic interfacial model ǀ Caveat: No major realism caveat captured in prior matrix	No / not central to this mechanistic record	Electrostatic; Pore filling/confinement	O-doped groups provide stable negative electrostatic sites; N-site effect is configuration-dependent; hierarchical pores improve transport and micropore filling	High convergence	No RQ2 flag identified	MD production length is relatively short compared with the strongest long-timescale studies; nevertheless, experiment–DFT–MD convergence is unusually complete.	RQ1, RQ2, RQ3, RQ4	Molecular depth; Evidence convergence; Environmental realism; Predictive transferability
49	A 2c	Synergistic adsorption mechanisms of self-N-doped biochar for levofloxacin elimination: integrated experimental and DFT studies ǀ DOI: 10.1016/j.envres.2025.122634	Levofloxacin	Self-N-doped porous biochar from waste peanut meal	Include — core	Strong	Yes	Yes	DFT on aromatic carbon clusters with graphitic/pyridinic/pyrrolic N; adsorption-energy and optimized-geometry analysis	M3 — Solvated/speciation-aware surface DFT ǀ Caveat: No major realism caveat captured in prior matrix	No / not central to this mechanistic record	Electrostatic; Hydrogen bonding; π interactions; Pore filling/confinement	Micropore filling and electrostatics are primary, with H-bonding and π–π EDA as supporting interactions	High convergence	Confirmed overinterpretation	Authors state Langmuir = monolayer chemisorption and PSO confirms chemical interaction; these fits do not independently prove mechanism.	RQ1, RQ2, RQ3, RQ4	Molecular depth; Evidence convergence; Environmental realism; Interpretive discipline; Predictive transferability
50	A 2c	Understanding the interaction mechanisms between nitroimidazole antibiotics and buckwheat hull biochar during adsorption process: Experiments and molecular dynamics simulations ǀ DOI: 10.1016/j.molliq.2024.126629	Metronidazole + tinidazole + ornidazole	Buckwheat-hull biochar	Include — core	Strong MD	Yes	Yes	Classical MD on a four-layer/slit-pore biochar model built from experimental elemental/functional-group data; OPLS-AA	M4 — Explicit-water MD / dynamic interfacial model ǀ Caveat: No major realism caveat captured in prior matrix	No / not central to this mechanistic record	Hydrogen bonding; π interactions; Pore filling/confinement	π–π interactions, H-bonding and pore filling; fast surface adsorption followed by slower intrapore diffusion	High convergence	False positive	Preliminary flag; false positive after full-text verification. Langmuir is described as monolayer adsorption and PSO is prominent; these fits remain descriptive.	RQ1, RQ2, RQ3, RQ4	Molecular depth; Evidence convergence; Interpretive discipline; Predictive transferability
51	A 2c	Adsorption of common greywater pollutants and nutrients by various biochars as potential amendments for nature-based systems: Laboratory tests and molecular dynamics ǀ DOI: 10.1016/j.envpol.2023.123203	Diclofenac, methylparaben, benzotriazole, decanesulfonate + nitrate, ammonium, phosphate, boron	Spruce-wood and Typha biochars at 350/600 °C	Include — core	Strong MD	Yes	No / not explicitly charted	GROMACS 100-ns MD; GAFF; DFT/B3LYP/6-31G* RESP charges; experimentally informed biochar models	M5 — Experimentally constrained explicit-water MD ǀ Caveat: No major realism caveat captured in prior matrix	No / not central to this mechanistic record	Electrostatic; Hydrogen bonding; π interactions; Hydrophobic	Organic pollutants mainly π–π with secondary H-bonding; nitrate/boron behavior linked to hydrophobic interactions; pyrolysis temperature shifts mechanism	Moderate / partial convergence	Confirmed overinterpretation	Paper occasionally maps Freundlich/Langmuir to heterogeneous/monolayer interpretations; model fit alone is not proof.	RQ1, RQ3	Molecular depth; Evidence convergence; Interpretive discipline

52	A 2c	Computational and experimental assessment of pH and specific ions on the solute solvent interactions of clay-biochar composites towards tetracycline adsorption: Implications on wastewater treatment | DOI: 10.1016/j.jenvman.2021.111989	Tetracycline	Municipal-waste biochar–clay composites	Include — core	Strong MD / solution chemistry	Yes	Yes	MD on B7/B17 biochar models in aqueous salt solutions; focuses on ions and solute–solvent interactions	M4 — Explicit-water MD / dynamic interfacial model | Caveat: No major realism caveat captured in prior matrix	No / not central to this mechanistic record	Not normalized / predictive study	Hydroxyl/carboxylate groups control solute–solvent interactions; tetracycline amide/hydroxyl groups interact with biochar oxygen groups	High convergence	No RQ2 flag identified	Composite contains clay, so biomass-specific contribution must be separated from mineral ion-exchange effects.	RQ1, RQ2, RQ3, RQ4	Molecular depth; Evidence convergence; Environmental realism; Predictive transferability
53	A 2c	Microscopic mechanism about the selective adsorption of Cr(VI) from salt solution on O-rich and N-rich biochars | DOI: 10.1016/j.jhazmat.2020.124162	Cr(VI) under competing anions	O-rich and N-rich poplar-leaf biochars	Include — core	Strong	Yes	Yes	Gaussian09 M06/6-31G(d,p) with PCM aqueous solution; interaction enthalpies for protonated biochar–$HCrO_4^-$ complexes	M2 — Explicit adsorbate–surface static DFT | Caveat: No major realism caveat captured in prior matrix	No / not central to this mechanistic record	Hydrogen bonding; Surface complexation/coordination	O-rich biochars retain Cr(VI) through carboxyl/hydroxyl complexation and strong H-bonds; N-rich sites are more vulnerable to anion competition	High convergence	Confirmed overinterpretation	PSO is explicitly interpreted as chemical adsorption; this is not independent proof.	RQ1, RQ3	Molecular depth; Evidence convergence; Interpretive discipline
54	A 2c	Sorption mechanisms of pesticides removal from effluent matrix using biochar: Conclusions from molecular modelling studies validated by single-, binary and ternary solute experiments | DOI: 10.1016/j.jenvman.2021.113104	Atrazine + imidacloprid + azoxystrobin	H_3PO_4-treated rice-straw biochar	Include — core	Strong	Yes	No / not explicitly charted	DFT-optimized biochar structure + Grand Canonical Monte Carlo + MD in Materials Studio; COMPASS II force field; adsorption energies	M4 — Explicit-water MD / dynamic interfacial model | Caveat: No major realism caveat captured in prior matrix	No / not central to this mechanistic record	Electrostatic; Pore filling/confinement	Pore filling, partitioning, aromatic nonbonding interactions and phosphate-ester electrostatics vary by pesticide and mixture composition	Moderate / partial convergence	Yes — the poor Langmuir fit is used to reject a monolayer homogeneous-surface mechanism; independent multisolute simulations corroborate parts of the interpretation but do not remove the local flag.	Some mechanistic labels are derived from isotherm models, but here they are independently challenged/validated by multisolute simulation and effluent experiments.	RQ1, RQ2, RQ3, RQ4	Molecular depth; Evidence convergence; Environmental realism; Predictive transferability
55	A 2c	Mechanistic insights into the simultaneous removal of As(V) and Cr(VI) oxyanions by a novel hierarchical corolla-like MnO_2-decorated porous magnetic biochar composite: A combined experimental and density functional theory study | DOI: 10.1016/j.apsusc.2021.151991	As(V) + Cr(VI)	Porous magnetic biochar/MnO_2 composite	Include — core	Strong multicomponent / redox-partitioned	Yes	Yes	DFT-supported interfacial interpretation combined with XPS and sequential adsorption design.	M2 — Explicit adsorbate–surface static DFT | Caveat: Static surface model; solvent treatment not established	No / not central to this mechanistic record	Surface complexation/coordination; Redox/transformation; van der Waals/NCI	As(V) adsorption is retained in mixed systems, while Cr(VI) competes more strongly and is partly reduced to Cr(III); adsorption and redox coexist.	High convergence	No RQ2 flag identified	Corrected here to Choi et al., Applied Surface Science 578 (2022) 151991. Reduction must be separated from adsorption.	RQ1, RQ2, RQ3, RQ4	Molecular depth; Evidence convergence; Predictive transferability

56	A2c	A Novel Graphitic Biochar Derived from Banana Peels for Efficient PFAS Removal: Mechanistic Insight from Integrated Experiments and DFT Calculations ǀ DOI: 10.3390/toxics14030204	10 legacy/alternative PFAS	ZnCl2-activated graphitic banana-peel biochar	Include — core	Strong	Yes	Yes	Gaussian16 B3LYP/6-31G(d,p), SMD water and DFT-D3; heptagonal graphene + XPS/FTIR-informed O/Zn functional groups; PFBA and PFOS representatives	M3 — Solvated/speciation-aware surface DFT ǀ Caveat: No major realism caveat captured in prior matrix	No / not central to this mechanistic record	Electrostatic; Hydrogen bonding; Hydrophobic; Surface complexation/coordination	Electrostatics, hydrophobic interaction, π–CF, surface complexation and H-bonding; Zn-associated sites contribute	High convergence	Confirmed overinterpretation	Abstract states PSO suggests chemisorption; PSO alone does not establish chemisorption. DFT models only two representative PFAS out of ten.	RQ1, RQ3	Molecular depth; Evidence convergence; Environmental realism; Interpretive discipline
57	A2c	A novel gallic acid modification and high-temperature alkali activation mesoporous biochar enables ultrafast and high-capacity removal of short- and long-chain PFAS from water ǀ DOI: 10.1016/j.cej.2026.174984	PFBA/PFBS/PFPeA/PFOA/PFOS and related PFAS	GA-modified, KOH-activated corncob mesoporous biochar	Include — core	Strong	Yes	Yes	DFT integrated with structure–activity analysis for short- and long-chain PFAS	M2 — Explicit adsorbate–surface static DFT ǀ Caveat: No major realism caveat captured in prior matrix	No / not central to this mechanistic record	Electrostatic; Pore filling/confinement; Hydrophobic; Surface complexation/coordination	Mesopore filling, electrostatic attraction, hydrophobic interaction and surface complexation jointly govern PFAS adsorption	High convergence	No RQ2 flag identified	PFAS is already well represented in the corpus; this paper adds value mainly through short-chain performance, mesoporosity and real-water validation.	RQ1, RQ2, RQ3, RQ4	Molecular depth; Evidence convergence; Environmental realism; Predictive transferability
58	A2c	Clorazepate and Diclofenac adsorption mechanisms on Al-coated açaí biochar: Insights from statistical physics and DFT calculations ǀ DOI: 10.1016/j.molstruc.2026.147134	Clorazepate + diclofenac	Al-coated açaí-seed biochar	Include — core	Moderate-to-strong	Yes	Yes	Statistical-physics adsorption models + conceptual DFT/Fukui/MEP descriptors	M1 — Indirect molecular descriptors / isolated-model evidence ǀ Caveat: Indirect/no explicit adsorbent surface	No / not central to this mechanistic record	van der Waals/NCI	Al coating changes surface heterogeneity, layer formation and preferred molecular interaction sites	Moderate / partial convergence	No RQ2 flag identified	Conceptual DFT reactivity descriptors are less direct than explicit adsorbate–surface binding calculations; statistical-physics energies should not be conflated with quantum adsorption energies.	RQ1, RQ3	Molecular depth; Evidence convergence
59	A2c	Density functional theory study of sulfur-functionalized defective biochar for heavy metal ions (Zn2+, Hg2+, Cd2+) adsorption ǀ DOI: 10.1016/j.fuel.2026.138381	Zn^{2+}, Hg^{2+}, Cd^{2+}	Sulfur-functionalized defective biochar models	Include — supporting / benchmark	Strong molecular / no new experiment	Yes	No / not explicitly charted	Gaussian16: B3LYP/SDD geometry; PBE0/SDD single points; DFT-D3(BJ); defect + sulfur-functional-group matrix	M2 — Explicit adsorbate–surface static DFT ǀ Caveat: Static surface model; solvent treatment not established	No / not central to this mechanistic record	van der Waals/NCI	Defects plus –S/–SO groups strengthen metal binding through charge redistribution and orbital interactions	Moderate / partial convergence	No RQ2 flag identified	Purely theoretical; cannot be counted as independent experiment–theory convergence. BSSE was not applied.	RQ1, RQ3, RQ4	Molecular depth; Evidence convergence; Predictive transferability

60	A2c	Efficient removal of metribuzin from water using modified beet molasses biochar: Adsorptive performance and mechanism | DOI: 10.1016/j.jwpe.2026.109855	Metribuzin (+ atrazine comparator)	KOH-modified beet-molasses biochar	Include — core	Strong	Yes	Yes	Gaussian16 M06-2X/def2-SVP with D3 and IEF-PCM water; higher-level single-point treatment; functional-group interaction analysis	M3 — Solvated/speciation-aware surface DFT | Caveat: No major realism caveat captured in prior matrix	No / not central to this mechanistic record	Electrostatic; Hydrogen bonding; π interactions; Pore filling/confinement	Pore filling is dominant, supplemented by electrostatics, H-bonding and π–π stacking; oxygen groups promote binding	High convergence	Confirmed overinterpretation	Yes — the PSO fit is interpreted as dominant chemisorption and the Freundlich fit as heterogeneous multilayer adsorption.	RQ1, RQ2, RQ3, RQ4	Molecular depth; Evidence convergence; Environmental realism; Predictive transferability
61	A2c	Multiscale synergistic effect of Fe/Mn oxides and biochar on bifunctional adsorption of tetracycline and Cu2+ in water at a wide pH range | DOI: 10.1016/j.seppur.2025.135783	Tetracycline + Cu^{2+}	Fe/Mn-oxide-modified mushroom-stick biochar	Include — core	Strong	Yes	Yes	DFT compares TC configurations/ESP across pH and adsorption energies/charge transfer for TC and Cu^{2+} on BC vs FMBC	M3 — Solvated/speciation-aware surface DFT | Caveat: No major realism caveat captured in prior matrix	No / not central to this mechanistic record	Electrostatic; Pore filling/confinement; Surface complexation/coordination	Hierarchical pore filling plus Fe/Mn-mediated complexation/electrostatics; mechanisms differ across pH and between TC and Cu^{2+}	High convergence	No RQ2 flag identified	Some narrative attributes pH effects to simple surface-charge rules even though TC speciation and metal coordination are more complex; interpret with the DFT configurations.	RQ1, RQ2, RQ3, RQ4	Molecular depth; Evidence convergence; Predictive transferability
62	A2c	The effect of microplastics on the adsorption of ofloxacin and Cu (II) by sawdust biochar | DOI: 10.1016/j.seppur.2025.136375	Ofloxacin + Cu(II) in presence of PS/PET microplastics	Sawdust biochar with fresh/aged PS or PET microplastics	Include — core	Strong complex-matrix / periodic DFT	Yes	Yes	Spin-polarized DFT; DMol3/VASP, PBE, PAW, DFT-D3; DOS calculations; explicit surface-adsorption configurations.	M2 — Explicit adsorbate–surface static DFT | Caveat: No major realism caveat captured in prior matrix	No / not central to this mechanistic record	Electrostatic; Hydrogen bonding; π interactions; Pore filling/confinement; Hydrophobic; Surface complexation/coordination; van der Waals/NCI	MPs act as both adsorbates and adsorbents; site competition and complexation alter OFL/Cu uptake; electrostatics, hydrophobicity, H-bonding, π–π, pore filling and Cu–OFL complexation contribute.	High convergence	Confirmed overinterpretation	Elovich fit is interpreted as mainly chemical adsorption and Langmuir/Freundlich fits as mono-/multilayer mechanism; these are not independent mechanistic proof.	RQ1, RQ2, RQ3, RQ4	Molecular depth; Evidence convergence; Environmental realism; Interpretive discipline; Predictive transferability

63	A2c	Adsorption Mechanism of Modified Biochar on Typical Alkyl and Chlorinated Organophosphorus Flame Retardants.; Organophosphorus Flame Retardants ǀ DOI: 10.19741/j.issn.1673-4831.2024.0680	TBOEP; TPrP; TDCPP (alkyl/chlorinated organophosphate flame retardants)	KOH-modified peanut-shell biochar; PBC600 selected among 500–800 °C chars	Include — core	Moderate — experimental/surface + molecular-descriptor DFT	Yes	Yes	Gaussian 09; B3LYP/6-31G(d) optimization of the three isolated OPFR molecules; ESP at 0.002 e·Bohr−3; Multiwfn molecular diameter and ESP descriptors.	M1 — Indirect molecular descriptors / isolated-model evidence ǀ Caveat: No major realism caveat captured in prior matrix	No / not central to this mechanistic record	Electrostatic; Hydrogen bonding; Pore filling/confinement; Hydrophobic; van der Waals/NCI	Pore filling, hydrophobic interactions and H-bonding support TBOEP/TPrP uptake; TDCPP additionally shows a weak electrostatic contribution. Experimental FTIR/EDS and water-matrix tests provide independent support, while DFT is indirect.	Moderate / partial convergence	Confirmed overinterpretation	Important RQ2 flag: authors infer TDCPP chemisorption from PSO and TBOEP physisorption from PFO. Langmuir is the best numerical fit but the authors then invoke non-uniform multilayer adsorption because predicted and experimental capacities disagree. DFT models only isolated pollutant molecules, so it does not directly validate pollutant–biochar binding.	RQ1, RQ2, RQ3, RQ4	Molecular depth; Evidence convergence; Environmental realism; Interpretive discipline; Predictive transferability
64	A2c	Iron and nitrogen co-doping biochar for simultaneous and efficient adsorption of oxytetracycline and norfloxacin from wastewater ǀ DOI: 10.1016/j.indcrop.2025.120646	Oxytetracycline + norfloxacin	Fe/N-enriched Enteromorpha clathrata biochar	Include — core	Strong site-specific DFT	Yes	Yes	Gaussian16 M06-2X/6-31G(d) with dispersion; graphene defect models including pyrrolic/pyridinic N and FeN4; Multiwfn/VMD weak-interaction/electronic analysis.	M2 — Explicit adsorbate–surface static DFT ǀ Caveat: No major realism caveat captured in prior matrix	No / not central to this mechanistic record	Electrostatic; Hydrogen bonding; π interactions; van der Waals/NCI	FeN4 is proposed as a high-affinity active site, with Fe–N/O chemical bonding plus H-bonding, π–π and electrostatic interactions.	High convergence	Confirmed overinterpretation	Very large binding energies (~−395 to −398 kcal/mol) warrant caution about model/reference conventions. Authors also equate Langmuir+PSO with monolayer chemisorption.	RQ1, RQ2, RQ3, RQ4	Molecular depth; Evidence convergence; Environmental realism; Interpretive discipline; Predictive transferability
65	A2c	Molecular insights into the simultaneous removal mechanisms of As(V) and Cd(II) in iron tailings slag-biochar composites ǀ DOI: 10.1016/j.jes.2024.04.030	As(V) + Cd(II)	Ginkgo-leaf biochar + iron-tailings slag composite	Include — core	Strong multitechnique / DFT	Yes	Yes	ORCA 5.0.4 DFT of iron-oxide/aluminum-oxide complexes with As(V), As(III) and Cd(II).	M2 — Explicit adsorbate–surface static DFT ǀ Caveat: Static surface model; solvent treatment	No / not central to this mechanistic record	Electrostatic; Surface complexation/coordination; Precipitation; Redox/transformation; van der Waals/NCI	Electrostatics, complexation and precipitation coexist; As(V) and Cd(II) show concentration-dependent synergistic and competitive effects;	High convergence	Unresolved	Removal is not pure adsorption: precipitation and redox/transformation contribute. Evidence-charting note: PFO/PSO and isotherm fits	RQ1, RQ3	Molecular depth; Evidence convergence

										not established			substantial As(V)→As(III) transformation occurs.			appear to be interpreted mechanistically; the full text could not be verified, and the case is excluded from the confirmed count.		
66	A 2c	Adsorptive removal of crystal violet using chemically activated biowaste: Molecular insights from DFT and molecular dynamics simulations and experimental assessment ∣ DOI: 10.1016/j.nxmate.2026.103177	Crystal violet	KOH-activated date-pit carbon	Include — supporting	Integrated DFT+MD supporting	Yes	Yes	DFT + molecular dynamics simulations used to evaluate CV–activated-carbon interactions and adsorption energies.	M4 — Explicit-water MD / dynamic interfacial model ∣ Caveat: No major realism caveat captured in prior matrix	No / not central to this mechanistic record	van der Waals/NCI	Heteroatoms and π-conjugated interactions support spontaneous adsorption on the activated-carbon surface.	Moderate–high convergence	No RQ2 flag identified	Dye adsorption is already heavily represented; mechanistic novelty is methodological rather than chemical. Model-fitting/RSM should not be used as molecular proof.	RQ1, RQ2, RQ3, RQ4	Molecular depth; Evidence convergence; Predictive transferability
67	A 2c	Ibuprofen adsorption using an alginate-functionalized manure/sunflower seed biochar composite: Characterization, optimization, and molecular simulation ∣ DOI: 10.1016/j.biombioe.2026.109617	Ibuprofen	Alginate-functionalized donkey-manure/sunflower-seed carbon composite	Include — supporting	Statistical-physics / composite supporting	Yes	Yes	Advanced statistical-physics adsorption modeling; molecular/energetic interpretation reported, but the reviewed methods emphasize statistical physics rather than explicit atomistic DFT/MD.	M1b — Statistical-physics / non-atomistic molecular interpretation ∣ Caveat: No major realism caveat captured in prior matrix	No / not central to this mechanistic record	Hydrogen bonding; π interactions; van der Waals/NCI	Predominantly physisorption with H-bonding, π–π and surface interactions; alginate oxygen groups contribute.	Moderate / partial convergence	False positive	Preliminary flag; false positive after full-text verification. The title says molecular simulation, but the main mechanistic methods reviewed are statistical-physics models. PSO/Langmuir are prominent; composite surface is chemically heterogeneous.	RQ1, RQ2, RQ3, RQ4	Molecular depth; Evidence convergence; Interpretive discipline; Predictive transferability
68	A 2c	Modification of biochar by iron containing adsorption centers as a method to enhance the remediation of perfluorooctanoic (PFOA) and (PFOS) acids from water and soil: a density functional theory study ∣ DOI: 10.1007/s00894-025-06491-9	PFOA + PFOS	Hematite adsorption-center model relevant to magnetic biochar	Include — supporting / molecular benchmark	High-rigor theory-only benchmark	Yes	No / not explicitly charted	Gaussian16 M06-2X; 6-31G(d,p) geometry, 6-311++G(d,p) single points; CPCM water; frequencies/Gibbs energies; counterpoise BSSE; Fe24O36 hematite model.	M3 — Solvated/speciation-aware surface DFT ∣ Caveat: Theory-only	No / not central to this mechanistic record	van der Waals/NCI	Iron-oxide adsorption centers provide strong PFAS interactions; charge/speciation and water are treated more carefully than in many cluster studies.	Moderate / partial convergence	No RQ2 flag identified	Theory-only; Fe24O36 is far smaller than experimental ~100-nm iron-oxide domains and authors acknowledge active-site/spin-state approximations.	RQ1, RQ3	Molecular depth; Evidence convergence

69	A2c	Rapid adsorptive removal of emerging and legacy per- and polyfluoroalkyl substances (PFASs) from water using zinc chloride-modified litchi seed-derived biochar ǀ DOI: 10.1016/j.biortech.2024.131157	10 legacy and emerging PFAS	ZnCl2-modified litchi-seed biochar	Include — core	Strong experiment–DFT / real-water	Yes	Yes	Gaussian16 B3LYP/6-31G(d,p), DFT-D3 and water solvent model; binding-energy calculations.	M3 — Solvated/speciation-aware surface DFT ǀ Caveat: No major realism caveat captured in prior matrix	No / not central to this mechanistic record	Electrostatic; Hydrogen bonding; Hydrophobic; Surface complexation/coordination; van der Waals/NCI	Surface complexation, electrostatics, H-bonding and hydrophobic interactions explain enhanced PFAS uptake after ZnCl2 modification.	High convergence	Confirmed overinterpretation	PSO is explicitly used to infer dominant chemisorption, and isotherm fits are translated into heterogeneity/multilayer claims. DFT representativeness across all ten PFAS should be checked.	RQ1, RQ3	Molecular depth; Evidence convergence; Environmental realism; Interpretive discipline
70	A2c	Soil amended with Algal Biochar Reduces Mobility of deicing salt contaminants in the environment: An atomistic insight ǀ DOI: 10.1016/j.chemosphere.2023.138172	Na+ / Cl− deicing-salt ions	Nitrogen-functionalized algal biochar used as soil/filter amendment	Include — contextual / boundary	Strong DFT+MD, scope-boundary	Yes	No / not explicitly charted	DFT + MD; direct Cl− adsorption and Na+-bridging; IGMH analysis; MD evaluates delayed chloride diffusion.	M4 — Explicit-water MD / dynamic interfacial model ǀ Caveat: No major realism caveat captured in prior matrix	No / not central to this mechanistic record	Electrostatic; Hydrogen bonding; Ion exchange/bridging; van der Waals/NCI	Nitrogenous groups promote Cl− retention via direct electrostatic/H-bond interactions and Na+ bridging; MD shows slower chloride diffusion.	Moderate / partial convergence	No RQ2 flag identified	Scope boundary: soil amendment/filter-media immobilization, not primary aqueous adsorption treatment. Retain for RQ3 methodological context only.	RQ1, RQ3	Molecular depth; Evidence convergence
71	A3	Gao Y.; Ran K.; Yang Y.; Wang P.; Yao J.; Li H.; Lin Z. (2026). Bridging biochar synthesis and dye wastewater treatment via an interpretable end-to-end machine learning framework. Resources Chemicals and Materials. DOI: 10.1016/j.recm.2026.100176	Not explicitly normalized in predictive audit	Multiple biomass-derived adsorbents / dataset-dependent	Include — core	Predictive / ML evidence	Yes / predictive study includes experimental component	Yes	Algorithms: RF; GBDT/GBR; DT; GPR/Kriging; Stacking XAI: SHAP; PDP; feature importance Descriptors: textural; surface/chemical; molecular; operational; preparation	Not applicable / no DFT model quality audit for predictive-only record	Validation: 10-fold CV; experimental validation; train/test split; XAI: SHAP; PDP; feature importance; descriptors: textural; surface/chemical; molecular; operational; preparation	Ion exchange/bridging	Predictive / descriptor-level evidence	Moderate: practical matrix tested, but not necessarily external ML validation	XAI/feature importance is not causal proof of an adsorption mechanism.	XAI/feature importance is not causal proof of an adsorption mechanism.	RQ4, RQ5	Predictive transferability; Interpretive discipline; Evidence convergence

72	A3	Al-Zyoud M.; Mostafa S.A.; Khersan I.; Gowrishankar J.; Sahu P.K.; Singla S.; Sabirov S.; Khudayberganov I.; Sherzod S. (2026). Effects of physical properties on the heavy metal adsorption of biochar via a robust approach. Current Research in Biotechnology. DOI: 10.1016/j.crbiot.2026.100367	Multiple / dataset-dependent	Multiple biomass-derived adsorbents / dataset-dependent	Include — core	Predictive / ML evidence	Yes / predictive study includes experimental component	Yes	Algorithms: RF; GBDT/GBR; CatBoost; ANN/BPNN; SVM/SVR; KNN; DT; CNN XAI: SHAP; sensitivity Descriptors: textural; surface/chemical; molecular; operational; preparation	Not applicable / no DFT model quality audit for predictive-only record	Validation: 5-fold CV; experimental validation; train/test split; XAI: SHAP; sensitivity; descriptors: textural; surface/chemical; molecular; operational; preparation	Not normalized / predictive study	Predictive / descriptor-level evidence	Moderate-to-high: internal validation only	Potential dataset/publication-family overlap with row 86; avoid treating evidence as fully independent until datasets are compared.; XAI/feature importance is not causal proof of an adsorption mechanism.; High reported R^2 should not be equated with transferability to unseen adsorbents or pollutants.	Potential dataset/publication-family overlap with row 86; avoid treating evidence as fully independent until datasets are compared.; XAI/feature importance is not causal proof of an adsorption mechanism.; High reported R^2 should not be equated with transferability to unseen adsorbents or pollutants.	RQ4, RQ5	Predictive transferability; Interpretive discipline; Evidence convergence
73	A3	Chen, K; Xiao, Q; Ji, JM; Chen, SW; Yang, ZJ; Tang, SG; Zhang, GX; Wang, LJ (2026). Efficient sulfadiazine removal from wastewater using lotus-based biochars: Characterization, adsorption, and machine learning prediction. Industrial Crops and Products. DOI: 10.1016/j.indcrop.2026.123791	Not explicitly normalized in predictive audit	Multiple biomass-derived adsorbents / dataset-dependent	Include — core	Predictive / ML evidence	Not necessarily; literature/dataset-based predictive evidence	Yes	Algorithms: RF; XGBoost; GBDT/GBR; ANN/BPNN; CNN; Stacking XAI: SHAP; feature importance Descriptors: textural; surface/chemical; molecular; operational; preparation	Not applicable / no DFT model quality audit for predictive-only record	Validation: train/test split; XAI: SHAP; feature importance; descriptors: textural; surface/chemical; molecular; operational; preparation	Not normalized / predictive study	Predictive / descriptor-level evidence	Moderate: practical matrix tested, but not necessarily external ML validation	XAI/feature importance is not causal proof of an adsorption mechanism.	XAI/feature importance is not causal proof of an adsorption mechanism.	RQ1, RQ2, RQ4, RQ5	Predictive transferability; Interpretive discipline
74	A3	Zhao, FZ; Jiang, HF; Xu, TR; Shi, YR; Jiang, XT; Chen, HM (2026). Efficiently quantifying thermodynamic and kinetic effects of biochar pyrolysis parameters on antibiotic adsorption from water using interpretable causal machine learning. Bioresource Technology. DOI: 10.1016/j.biortech.2026.134793	Not explicitly normalized in predictive audit	Multiple biomass-derived adsorbents / dataset-dependent	Include — core	Predictive / ML evidence	Not necessarily; literature/dataset-based predictive evidence	Yes	Algorithms: RF; XGBoost; GBDT/GBR; SVM/SVR; KNN; DT XAI: SHAP; PDP; feature importance; sensitivity; causal inference Descriptors: textural; surface/chemical; molecular; operational; preparation	Not applicable / no DFT model quality audit for predictive-only record	Validation: external validation; train/test split; XAI: SHAP; PDP; feature importance; sensitivity; causal inference; descriptors: textural;	Not normalized / predictive study	Predictive / descriptor-level evidence	Moderate: some external/independent validation	XAI/feature importance is not causal proof of an adsorption mechanism.	XAI/feature importance is not causal proof of an adsorption mechanism.	RQ5	Predictive transferability; Interpretive discipline; Evidence convergence

											surface/chemical; molecular; operational; preparation							
75	A3	Ravindiran G.; Karthick K.; Ramujee K.; Christo M.S.; Allayarovich M.B.; Nurmatovich H.A.; Ali S.A.; Hayder G. (2026). Ensemble Machine Learning Prediction of Multi-Pollutant Removal Efficiency in Biochar-Based Adsorption Systems. International Journal of Environmental Research. DOI: 10.1007/s41742-025-01011-2	Multiple / dataset-dependent	Multiple biomass-derived adsorbents / dataset-dependent	Include — core	Predictive / ML evidence	Yes / predictive study includes experimental component	Yes	Algorithms: RF; XGBoost; GBDT/GBR; CatBoost; ANN/BPNN; SVM/SVR; KNN; DT XAI: SHAP; feature importance Descriptors: textural; surface/chemical; molecular; operational; preparation	Not applicable / no DFT model quality audit for predictive-only record	Validation: experimental validation; train/test split; XAI: SHAP; feature importance; descriptors: textural; surface/chemical; molecular; operational; preparation	Not normalized / predictive study	Predictive / descriptor-level evidence	Moderate: practical matrix tested, but not necessarily external ML validation	XAI/feature importance is not causal proof of an adsorption mechanism.	XAI/feature importance is not causal proof of an adsorption mechanism.	RQ5	Predictive transferability; Interpretive discipline; Evidence convergence
76	A3	Zhang, SS; Shen, L; Qiang, J; Liu, HH; Xu, JQ; He, JQ; Shi, JY; Gao, YT (2026). Fe-N co-modified lotus stalk biochar for TCH adsorption from aqueous solution: Performance, mechanism, machine learning modeling, and SHAP interpretation. Journal of Environmental Chemical Engineering. DOI: 10.1016/j.jece.2026.122945	Not explicitly normalized in predictive audit	Multiple biomass-derived adsorbents / dataset-dependent	Include — core	Predictive / ML evidence	Not necessarily; literature /dataset-based predictive evidence	Yes	Algorithms: RF; XGBoost; GBDT/GBR; ANN/BPNN; SVM/SVR; DT; GPR/Kriging; Stacking XAI: SHAP; PDP; feature importance Descriptors: textural; surface/chemical; molecular; operational; preparation	Not applicable / no DFT model quality audit for predictive-only record	Validation: 5-fold CV; external validation; train/test split; XAI: SHAP; PDP; feature importance; descriptors: textural; surface/chemical; molecular; operational; preparation	Not normalized / predictive study	Predictive / descriptor-level evidence	Moderate: some external/independent validation	XAI/feature importance is not causal proof of an adsorption mechanism.	XAI/feature importance is not causal proof of an adsorption mechanism.	RQ1, RQ2, RQ4, RQ5	Predictive transferability; Interpretive discipline; Evidence convergence
77	A3	Hafshejani L.D.; Saeidinia M. (2026). Integrating chemical modification pathways and machine learning for optimization of nitrate removal by rapeseed (Brassica napus L.) biochar.	Not explicitly normalized in predictive audit	Multiple biomass-derived adsorbents / dataset-dependent	Include — core	Predictive / ML evidence	Yes / predictive study includes experimental component	Yes	Algorithms: RF; SVM/SVR; DT XAI: SHAP; feature importance; sensitivity Descriptors: textural; surface/chemical; operational; preparation	Not applicable / no DFT model quality audit for predictive-	Validation: 5-fold CV; K-fold CV; external validation; experimental validation;	Not normalized / predictive study	Predictive / descriptor-level evidence	Moderate: some external/independent validation	XAI/feature importance is not causal proof of an adsorption mechanism.	XAI/feature importance is not causal proof of an adsorption mechanism.	RQ4, RQ5	Predictive transferability; Interpretive discipline; Evidence

		Scientific Reports. DOI: 10.1038/s41598-025-29743-3								only record	train/test split; XAI: SHAP; feature importance; sensitivity; descriptors: textural; surface/chemical; operational; preparation							convergence
78	A3	Sudarsan S.; Basavaraja G.; Vinayagam R.; Selvaraj R. (2026). Interpretable machine learning and greenness assessment for sustainable tetracycline adsorption and phytotoxicity mitigation using almond peel – derived activated carbon. Chemosphere. DOI: 10.1016/j.chemosphere.2026.144985	Not explicitly normalized in predictive audit	Not explicitly normalized in predictive audit	Include — core	Predictive / ML evidence	Yes / predictive study includes experimental component	Yes	Algorithms: RF; XGBoost; GBDT/GBR; CatBoost; ANN/BPNN; SVM/SVR; DT; AdaBoost XAI: SHAP; PDP; feature importance Descriptors: textural; surface/chemical; molecular; operational	Not applicable / no DFT model quality audit for predictive-only record	Validation: 5-fold CV; external validation; experimental validation; train/test split; XAI: SHAP; PDP; feature importance; descriptors: textural; surface/chemical; molecular; operational	Not normalized / predictive study	Predictive / descriptor-level evidence	Moderate: some external/independent validation	XAI/feature importance is not causal proof of an adsorption mechanism.	XAI/feature importance is not causal proof of an adsorption mechanism.	RQ4, RQ5	Predictive transferability; Interpretive discipline; Evidence convergence
79	A3	Huang, X; Bai, XP; Yang, YF; Li, WB; Xu, DC (2026). Machine Learning-Based Prediction and Optimization of Heavy Metal Adsorption Performance of Biochar. Forests. DOI: 10.3390/f17030326	Multiple / dataset-dependent	Multiple biomass-derived adsorbents / dataset-dependent	Include — core	Predictive / ML evidence	Not necessarily; literature/dataset-based predictive evidence	Yes	Algorithms: XGBoost; GBDT/GBR; SVM/SVR; DT; CNN; AdaBoost XAI: SHAP; feature importance Descriptors: textural; surface/chemical; molecular; operational; preparation	Not applicable / no DFT model quality audit for predictive-only record	Validation: train/test split; XAI: SHAP; feature importance; descriptors: textural; surface/chemical; molecular; operational; preparation	Not normalized / predictive study	Predictive / descriptor-level evidence	High/unclear: transferability not demonstrated	XAI/feature importance is not causal proof of an adsorption mechanism.	XAI/feature importance is not causal proof of an adsorption mechanism.	RQ5	Predictive transferability; Interpretive discipline

80	A3	Gümüş D.; Gümüş F.; Kadioğlu E.N.; Eroğlu H.A. (2026). Machine Learning-Guided Prediction and Interpretation of Rhodamine B Removal by Biochar Derived from Marine Biomass. Water, Air, and Soil Pollution. DOI: 10.1007/s11270-025-09054-z	Not explicitly normalized in predictive audit	Multiple biomass-derived adsorbents / dataset-dependent	Include — core	Predictive / ML evidence	Yes / predictive study includes experimental component	Yes	Algorithms: RF; XGBoost; GBDT/GBR; CatBoost; ANN/BPNN; SVM/SVR XAI: SHAP; PDP; feature importance Descriptors: textural; surface/chemical; molecular; operational; preparation	Not applicable / no DFT model quality audit for predictive-only record	Validation: 5-fold CV; external validation; experimental validation; train/test split; XAI: SHAP; PDP; feature importance; descriptors: textural; surface/chemical; molecular; operational; preparation	Not normalized / predictive study	Predictive / descriptor-level evidence	Moderate: some external/independent validation	XAI/feature importance is not causal proof of an adsorption mechanism.; Check the full input list for possible target leakage before using mechanistic feature rankings.	XAI/feature importance is not causal proof of an adsorption mechanism.; Check the full input list for possible target leakage before using mechanistic feature rankings.	RQ4, RQ5	Predictive transferability; Interpretive discipline; Evidence convergence
81	A3	Shafiq M.; Asif M.; Amin M.T.; Bilal M.; Alazba A.A. (2026). Machine learning-driven prediction and mechanistic interpretation of heavy metal adsorption by biomass-derived adsorbents. Chemical Papers. DOI: 10.1007/s11696-026-04879-2	Multiple / dataset-dependent	Not explicitly normalized in predictive audit	Include — core	Predictive / ML evidence	Not necessarily; literature/dataset-based predictive evidence	Yes	Algorithms: ANN/BPNN XAI: SHAP Descriptors: textural; surface/chemical	Not applicable / no DFT model quality audit for predictive-only record	Validation: Validation details require targeted extraction; XAI: SHAP; descriptors: textural; surface/chemical	Not normalized / predictive study	Predictive / descriptor-level evidence	High/unclear: transferability not demonstrated	XAI/feature importance is not causal proof of an adsorption mechanism.	XAI/feature importance is not causal proof of an adsorption mechanism.	RQ1, RQ5	Predictive transferability; Interpretive discipline
82	A3	Adeleye A.T.; Bahar M.M.; Megharaj M.; Rahman M.M. (2026). Mechanistic and machine-learning insights into microplastic adsorption on modified magnetic biochar for circular-economy applications. Journal of Industrial and Engineering Chemistry. DOI: 10.1016/j.jiec.2026.02.036	Not explicitly normalized in predictive audit	Multiple biomass-derived adsorbents / dataset-dependent	Include — core	Predictive / ML evidence	Yes / predictive study includes experimental component	Yes	Algorithms: RF; GBDT/GBR; SVM/SVR; DT; Stacking XAI: SHAP; PDP; feature importance; sensitivity Descriptors: textural; surface/chemical; molecular; operational; preparation	Not applicable / no DFT model quality audit for predictive-only record	Validation: 5-fold CV; experimental validation; XAI: SHAP; PDP; feature importance; sensitivity; descriptors: textural; surface/chemical; molecular; operational; preparation	Not normalized / predictive study	Predictive / descriptor-level evidence	Moderate: practical matrix tested, but not necessarily external ML validation	XAI/feature importance is not causal proof of an adsorption mechanism.	XAI/feature importance is not causal proof of an adsorption mechanism.	RQ1, RQ2, RQ4, RQ5	Predictive transferability; Interpretive discipline; Evidence convergence

83	A3	Saeidi N.; Vicente D.J.; Chaudhuri S.; Georgi A. (2026). Modeling adsorption of mobile organic compounds on activated carbon and biochar using machine learning. Journal of Environmental Chemical Engineering. DOI: 10.1016/j.jece.2026.123911	Not explicitly normalized in predictive audit	Multiple biomass-derived adsorbents / dataset-dependent	Include — core	Predictive / ML evidence	Not necessarily; literature /dataset-based predictive evidence	Yes	Algorithms: ML model(s) reported XAI: SHAP; PDP Descriptors: textural; surface/chemical; molecular; operational	Not applicable / no DFT model quality audit for predictive-only record	Validation: train/test split (80/20); 10-fold CV; compound-grouped 10-fold CV (GroupKFold); external validation (independent held-out set, 23 log Kd values for 14 compounds); experimental validation; leakage control (scaling fitted within folds); applicability domain (Mahalanobis distance); XAI: SHAP; PDP; descriptors: textural; surface/chemical; molecular; operational	Not normalized / predictive study	Predictive / descriptor-level evidence	Low-to-moderate: chemical-space generalization explicitly tested (compound-grouped CV and independent external set)	XAI/feature importance is not causal proof of an adsorption mechanism.	Performance decreased from $R^2 \approx 0.81$ (random split) to median $R^2 \approx 0.51$ (compound-grouped CV) and $R^2 \approx 0.64$ on the independent set, indicating that random-split accuracy overstates transferability to unseen compounds. XAI/feature importance is not causal proof of an adsorption mechanism.	RQ4, RQ5	Predictive transferability; Interpretive discipline
84	A3	Haq H.U.; Yasir M.; Khan M.N.A.; Gul J.; Zubair M.; Ali H.; Sedlarik V.; Ahmad N.M. (2026). Optimized adsorption removal and capacity prediction of anionic pollutants using a hybrid strategy of machine learning algorithms. Separation and Purification Technology. DOI: 10.1016/j.seppur.2026.139012	Not explicitly normalized in predictive audit	Not explicitly normalized in predictive audit	Include — core	Predictive / ML evidence	Yes / predictive study includes experimental component	Yes	Algorithms: RF; ANN/BPNN; SVM/SVR; DT; GPR/Kriging XAI: PDP; feature importance; sensitivity Descriptors: textural; surface/chemical; molecular; operational; preparation	Not applicable / no DFT model quality audit for predictive-only record	Validation: 5-fold CV; experimental validation; train/test split; XAI: PDP; feature importance; sensitivity; descriptors: textural; surface/che	Not normalized / predictive study	Predictive / descriptor-level evidence	Moderate: practical matrix tested, but not necessarily external ML validation	XAI/feature importance is not causal proof of an adsorption mechanism.	XAI/feature importance is not causal proof of an adsorption mechanism.	RQ4, RQ5	Predictive transferability; Interpretive discipline; Evidence convergence

											mical; molecular; operational ; preparatio n							
8 5	A 3	Abu-Shareha A.A.; Alfilh R.; Yaseen A.M.; Sudhamsu G.; Sahu P.K.; Jenifer D R.; Sharma S.; Jain V.; Hekmatyar Z. (2026). Robust Data driven modeling of Cd(II) adsorption on biochar. Journal of Hazardous Materials Advances. DOI: 10.1016/j.hazadv.2026.10100 4	Not explicitly normalized in predictive audit	Multiple biomass-derived adsorbents / dataset-dependent	Include — core	Predictive / ML evidence	Yes / predictiv e study includes experime ntal compone nt	Yes	Algorithms: RF; GBDT/GBR; ANN/BPNN; SVM/SVR; KNN; DT; GPR/Kriging; CNN XAI: SHAP; sensitivity Descriptors: textural; surface/chemical; molecular; operational; preparation	Not applicable / no DFT model quality audit for predictive-only record	Validation: 5-fold CV; K-fold CV; external validation; experiment al validation; train/test split; XAI: SHAP; sensitivity; descriptors : textural; surface/che mical; molecular; operational ; preparatio n	Not normalized / predictive study	Predictive / descriptor-level evidence	Moderate: some external/in dependent validation	XAI/feature importance is not causal proof of an adsorption mechanism.	XAI/feature importance is not causal proof of an adsorption mechanism.	RQ5	Predictive transferab ility; Interpreti ve discipline; Evidence converge nce
8 6	A 3	Al-Zyoud M.; Mostafa S.A.; Khersan I.; Gowrishankar J.; Sahu P.K.; Singla S.; Sabirov S.; Khudayberganov I.; Sherzod S. (2026). Stochastic optimization of Gradient Boosting Decision Trees for interpretable prediction of heavy metal adsorption onto biochar. Current Research in Biotechnology. DOI: 10.1016/j.crbiot.2026.100393	Multiple / dataset-dependent	Multiple biomass-derived adsorbents / dataset-dependent	Include — core	Predictive / ML evidence	Not necessaril y; literature /dataset-based predictiv e evidence	Yes	Algorithms: RF; XGBoost; GBDT/GBR; CatBoost; LightGBM; ANN/BPNN; SVM/SVR; DT XAI: SHAP; feature importance Descriptors: textural; surface/chemical; molecular; operational; preparation	Not applicable / no DFT model quality audit for predictive-only record	Validation: 5-fold CV; K-fold CV; train/test split; XAI: SHAP; feature importance ; descriptors : textural; surface/che mical; molecular; operational ; preparatio n	Not normalized / predictive study	Predictive / descriptor-level evidence	Moderate: practical matrix tested, but not necessarily external ML validation	Potential dataset/public ation-family overlap with row 72; avoid treating evidence as fully independent until datasets are compared.; XAI/feature importance is not causal proof of an adsorption mechanism.	Potential dataset/publicat ion-family overlap with row 72; avoid treating evidence as fully independent until datasets are compared.; XAI/feature importance is not causal proof of an adsorption mechanism.	RQ4, RQ5	Predictive transferab ility; Interpreti ve discipline

87	A3	Zhao, CQ; Gong, SN; Tan, W; Liu, YX; Pan, HH; Huang, SY; Shi, Q; Li, ZY (2026). Targeted preparation of graphene-like biochar for efficient tetracycline adsorption: Performance, mechanism, and machine-learning applications. INDUSTRIAL CROPS AND PRODUCTS. DOI: 10.1016/j.indcrop.2026.123780	Not explicitly normalized in predictive audit	Multiple biomass-derived adsorbents / dataset-dependent	Include — core	Predictive / ML evidence	Not necessarily; literature /dataset-based predictive evidence	Yes	Algorithms: RF; XGBoost; GBDT/GBR; SVM/SVR; DT; Stacking XAI: PDP; feature importance Descriptors: textural; surface/chemical; molecular; operational; preparation	Not applicable / no DFT model quality audit for predictive-only record	Validation: train/test split; XAI: PDP; feature importance; descriptors: textural; surface/chemical; molecular; operational; preparation	Not normalized / predictive study	Predictive / descriptor-level evidence	Moderate: practical matrix tested, but not necessarily external ML validation	XAI/feature importance is not causal proof of an adsorption mechanism.	XAI/feature importance is not causal proof of an adsorption mechanism.	RQ4, RQ5	Predictive transferability; Interpretive discipline
88	A3	Murugeshwari S.; Kumar R.M.S.; Rathi B.S.; Kishore V.; Sridhar S.; Divya J. (2026). Thermochemical valorization of mixed biomass into activated hydrochar for rapid Congo red removal: Integrating adsorption studies with machine learning. Next Materials. DOI: 10.1016/j.nxmate.2026.102874	Not explicitly normalized in predictive audit	Not explicitly normalized in predictive audit	Include — core	Predictive / ML evidence	Not necessarily; literature /dataset-based predictive evidence	Yes	Algorithms: RF; GBDT/GBR; DT XAI: feature importance Descriptors: textural; surface/chemical; operational; preparation	Not applicable / no DFT model quality audit for predictive-only record	Validation: LOOCV; XAI: feature importance; descriptors: textural; surface/chemical; operational; preparation	Not normalized / predictive study	Predictive / descriptor-level evidence	Moderate: practical matrix tested, but not necessarily external ML validation	XAI/feature importance is not causal proof of an adsorption mechanism.	XAI/feature importance is not causal proof of an adsorption mechanism.	RQ1, RQ2, RQ4, RQ5	Predictive transferability; Interpretive discipline
89	A3	Jha D.K. (2026). Unveiling sulfonamide adsorption on biochar using explainable machine learning. Bioresource Technology. DOI: 10.1016/j.biortech.2025.133867	Not explicitly normalized in predictive audit	Multiple biomass-derived adsorbents / dataset-dependent	Include — core	Predictive / ML evidence	Yes / predictive study includes experimental component	Yes	Algorithms: RF; XGBoost; GBDT/GBR; ANN/BPNN; SVM/SVR; DT; GPR/Kriging; Stacking XAI: SHAP; PDP; feature importance Descriptors: textural; surface/chemical; molecular; operational; preparation	Not applicable / no DFT model quality audit for predictive-only record	Validation: 5-fold CV; experimental validation; train/test split; XAI: SHAP; PDP; feature importance; descriptors: textural; surface/chemical; molecular; operational; preparation	Not normalized / predictive study	Predictive / descriptor-level evidence	Moderate: practical matrix tested, but not necessarily external ML validation	XAI/feature importance is not causal proof of an adsorption mechanism.; Very high in-domain accuracy; literature-derived heterogeneous data still require external validation for transferability.	XAI/feature importance is not causal proof of an adsorption mechanism.; Very high in-domain accuracy; literature-derived heterogeneous data still require external validation for transferability.	RQ4, RQ5	Predictive transferability; Interpretive discipline; Evidence convergence

90	A3	Dhillayan D.; Bhukal S.; Choudhary R.; Singh A.; Ghotekar S.; Garg V.K.; Kataria N. (2026). Upcycling of Eichhornia crassipes to modified biochar for emerging pollutant (Bisphenol-A) removal: optimization with machine learning and response surface methodology. Journal of Industrial and Engineering Chemistry. DOI: 10.1016/j.jiec.2026.05.020	Not explicitly normalized in predictive audit	Multiple biomass-derived adsorbents / dataset-dependent	Include — core	Predictive / ML evidence	Not necessarily; literature /dataset-based predictive evidence	Yes	Algorithms: RF; GBDT/GBR; CatBoost; DT; Stacking XAI: SHAP; feature importance Descriptors: textural; surface/chemical; molecular; operational; preparation	Not applicable / no DFT model quality audit for predictive-only record	Validation: train/test split; XAI: SHAP; feature importance; descriptors: textural; surface/chemical; molecular; operational; preparation	Not normalized / predictive study	Predictive / descriptor-level evidence	Moderate: practical matrix tested, but not necessarily external ML validation	XAI/feature importance is not causal proof of an adsorption mechanism.	XAI/feature importance is not causal proof of an adsorption mechanism.	RQ5	Predictive transferability; Interpretive discipline
91	A3	Temiz Seymen S.; Atalay Eroglu H.; Kadioglu E.N.; Akbal F. (2025). Artificial intelligence-assisted prediction of amoxicillin removal from wastewater using biomass-derived activated carbons. Journal of Environmental Chemical Engineering. DOI: 10.1016/j.jece.2025.118251	Not explicitly normalized in predictive audit	Not explicitly normalized in predictive audit	Include — core	Predictive / ML evidence	Not necessarily; literature /dataset-based predictive evidence	Yes	Algorithms: RF; XGBoost; GBDT/GBR; ANN/BPNN; DT; Stacking XAI: feature importance Descriptors: textural; surface/chemical; molecular; operational; preparation	Not applicable / no DFT model quality audit for predictive-only record	Validation: 5-fold CV; train/test split; XAI: feature importance; descriptors: textural; surface/chemical; molecular; operational; preparation	Not normalized / predictive study	Predictive / descriptor-level evidence	Moderate: practical matrix tested, but not necessarily external ML validation	XAI/feature importance is not causal proof of an adsorption mechanism.	XAI/feature importance is not causal proof of an adsorption mechanism.	RQ1, RQ2, RQ4, RQ5	Predictive transferability; Interpretive discipline
92	A3	Kubar G.M.; Kubar A.A.; Kubar K.A.; Shahzad K. (2025). Biochar-Based Water Remediation: A Machine Learning Approach to Antibiotic Adsorption Prediction. Water, Air, and Soil Pollution. DOI: 10.1007/s11270-025-08651-2	Not explicitly normalized in predictive audit	Multiple biomass-derived adsorbents / dataset-dependent	Include — core	Predictive / ML evidence	Not necessarily; literature /dataset-based predictive evidence	Yes	Algorithms: RF; SVM/SVR; Stacking XAI: PDP; feature importance Descriptors: textural; surface/chemical; molecular; operational; preparation	Not applicable / no DFT model quality audit for predictive-only record	Validation: train/test split; XAI: PDP; feature importance; descriptors: textural; surface/chemical; molecular; operational; preparation	Not normalized / predictive study	Predictive / descriptor-level evidence	Moderate: practical matrix tested, but not necessarily external ML validation	XAI/feature importance is not causal proof of an adsorption mechanism.; Reported test performance is weak relative to training in this study family; valuable counterexample for overfitting/generalization.	XAI/feature importance is not causal proof of an adsorption mechanism.; Reported test performance is weak relative to training in this study family; valuable counterexample for overfitting/generalization.	RQ1, RQ2, RQ4, RQ5	Predictive transferability; Interpretive discipline

93	A3	Xiang X.; Jia D.; Yang Z.; Jiang F.; Yang T.; Cao J. (2025). Cd adsorption prediction of Fe mono/composite modified biochar based on machine learning: Application for controllable preparation. Environmental Research. DOI: 10.1016/j.envres.2024.120466	Not explicitly normalized in predictive audit	Multiple biomass-derived adsorbents / dataset-dependent	Include — core	Predictive / ML evidence	Yes / predictive study includes experimental component	Yes	Algorithms: RF; ANN/BPNN; GPR/Kriging XAI: SHAP; feature importance Descriptors: textural; surface/chemical; operational; preparation	Not applicable / no DFT model quality audit for predictive-only record	Validation: external validation; experimental validation; train/test split; XAI: SHAP; feature importance; descriptors: textural; surface/chemical; operational; preparation	Not normalized / predictive study	Predictive / descriptor-level evidence	Moderate: some external/independent validation	XAI/feature importance is not causal proof of an adsorption mechanism.	XAI/feature importance is not causal proof of an adsorption mechanism.	RQ1, RQ2, RQ4, RQ5	Predictive transferability; Interpretive discipline; Evidence convergence
94	A3	Zeng Z.; Wu Y.; Li W.; Han L.; Zhao H.; Teng R.; Yuan H.; Wang Z.; Li S. (2025). Comparative analysis of multiple machine learning models: identifying impact factors in biochar heavy metal adsorption mechanisms. Environmental Geochemistry and Health. DOI: 10.1007/s10653-025-02734-z	Multiple / dataset-dependent	Multiple biomass-derived adsorbents / dataset-dependent	Include — core	Predictive / ML evidence	Not necessarily; literature /dataset-based predictive evidence	Yes	Algorithms: RF; XGBoost; GBDT/GBR; CatBoost; LightGBM; ANN/BPNN; SVM/SVR; DT XAI: SHAP; feature importance Descriptors: textural; surface/chemical; operational; preparation	Not applicable / no DFT model quality audit for predictive-only record	Validation: train/test split; XAI: SHAP; feature importance; descriptors: textural; surface/chemical; operational; preparation	Not normalized / predictive study	Predictive / descriptor-level evidence	Moderate: practical matrix tested, but not necessarily external ML validation	XAI/feature importance is not causal proof of an adsorption mechanism.	XAI/feature importance is not causal proof of an adsorption mechanism.	RQ5	Predictive transferability; Interpretive discipline
95	A3	Muteeb G.; Alshoaibi A.; Ansari K. (2025). Experimental optimization and machine learning modeling for sustainable Congo red dye removal from wastewater using activated goat bone biochar. Journal of Science: Advanced Materials and Devices. DOI: 10.1016/j.jsamd.2025.100947	Not explicitly normalized in predictive audit	Multiple biomass-derived adsorbents / dataset-dependent	Include — supporting	Predictive / ML evidence	Yes / predictive study includes experimental component	Yes	Algorithms: RF; GBDT/GBR; ANN/BPNN; SVM/SVR; DT; Stacking XAI: None/limited Descriptors: textural; surface/chemical; operational; preparation	Not applicable / no DFT model quality audit for predictive-only record	Validation: experimental validation; train/test split; XAI: None/limited; descriptors: textural; surface/chemical; operational; preparation	Not normalized / predictive study	Predictive / descriptor-level evidence	High/unclear: transferability not demonstrated	No special warning beyond standard XAI/transferability caution.	No special warning beyond standard XAI/transferability caution.	RQ1, RQ2, RQ4, RQ5	Predictive transferability; Interpretive discipline; Evidence convergence

96	A3	Choi G.-R.; Yang H.; Lee J.H.; Runfa T.; Cho I.S.; Park S.-J.; Lee C.-G.; Kang J.-K. (2025). Explainable AI-based evaluation of factors affecting heavy metal removal by microalgae-based adsorbents. Journal of Applied Phycology. DOI: 10.1007/s10811-025-03626-6	Multiple / dataset-dependent	Not explicitly normalized in predictive audit	Include — core	Predictive / ML evidence	Not necessarily; literature /dataset-based predictive evidence	Yes	Algorithms: XGBoost; GBDT/GBR; ANN/BPNN; DT XAI: SHAP Descriptors: textural; surface/chemical; molecular; operational; preparation	Not applicable / no DFT model quality audit for predictive-only record	Validation: 5-fold CV; XAI: SHAP; descriptors: textural; surface/chemical; molecular; operational; preparation	Not normalized / predictive study	Predictive / descriptor-level evidence	Moderate: practical matrix tested, but not necessarily external ML validation	XAI/feature importance is not causal proof of an adsorption mechanism.	XAI/feature importance is not causal proof of an adsorption mechanism.	RQ1, RQ5	Predictive transferability; Interpretive discipline
97	A3	Tian K.; Li C.; Liu H.; Wang L. (2025). Functionalization of biochar using SDS/SAP nanomicelles enhanced its immobilization capacity for dyes and heavy metals in water. Scientific Reports. DOI: 10.1038/s41598-025-91229-z	Multiple / dataset-dependent	Multiple biomass-derived adsorbents / dataset-dependent	Include — core	Predictive / ML evidence	Yes / predictive study includes experimental component	Yes	Algorithms: RF; XGBoost; GBDT/GBR; CatBoost; LightGBM; SVM/SVR; DT; GPR/Kriging XAI: SHAP; PDP; feature importance; sensitivity Descriptors: textural; surface/chemical; molecular; operational; preparation	Not applicable / no DFT model quality audit for predictive-only record	Validation: 10-fold CV; external validation; experimental validation; train/test split; XAI: SHAP; PDP; feature importance; sensitivity; descriptors: textural; surface/chemical; molecular; operational; preparation	Not normalized / predictive study	Predictive / descriptor-level evidence	Moderate: some external/independent validation	XAI/feature importance is not causal proof of an adsorption mechanism.	XAI/feature importance is not causal proof of an adsorption mechanism.	RQ1, RQ2, RQ4, RQ5	Predictive transferability; Interpretive discipline; Evidence convergence
98	A3	Yaseen Z.M.; Alhalimi F.L. (2025). Heavy metal adsorption efficiency prediction using biochar properties: a comparative analysis for ensemble machine learning models. Scientific Reports. DOI: 10.1038/s41598-025-96271-5	Multiple / dataset-dependent	Multiple biomass-derived adsorbents / dataset-dependent	Include — core	Predictive / ML evidence	Not necessarily; literature /dataset-based predictive evidence	Yes	Algorithms: RF; XGBoost; GBDT/GBR; ANN/BPNN; SVM/SVR; KNN; DT; Stacking XAI: None/limited Descriptors: textural; surface/chemical; molecular; operational; preparation	Not applicable / no DFT model quality audit for predictive-only record	Validation: 5-fold CV; K-fold CV; train/test split; XAI: None/limited; descriptors: textural; surface/chemical; molecular; operational; preparation	Not normalized / predictive study	Predictive / descriptor-level evidence	Moderate-to-high: internal validation only	No special warning beyond standard XAI/transferability caution.	No special warning beyond standard XAI/transferability caution.	RQ4, RQ5	Predictive transferability; Interpretive discipline

99	A3	Ullah, H; Khan, S; Zhu, XY; Chen, BL; Rao, ZP; Wu, NC; Idris, AM (2025). Machine learning-aided biochar design for the adsorptive removal of emerging inorganic pollutants in water. Separation and Purification Technology. DOI: 10.1016/j.seppur.2025.131421	Not explicitly normalized in predictive audit	Multiple biomass-derived adsorbents / dataset-dependent	Include — core	Predictive / ML evidence	Not necessarily; literature /dataset-based predictive evidence	Yes	Algorithms: RF; XGBoost; GBDT/GBR; CatBoost; LightGBM; ANN/BPNN; SVM/SVR; KNN XAI: SHAP; PDP; feature importance Descriptors: textural; surface/chemical; molecular; operational; preparation	Not applicable / no DFT model quality audit for predictive-only record	Validation: train/test split; XAI: SHAP; PDP; feature importance; descriptors: textural; surface/chemical; molecular; operational; preparation	Not normalized / predictive study	Predictive / descriptor-level evidence	High/unclear: transferability not demonstrated	XAI/feature importance is not causal proof of an adsorption mechanism.	XAI/feature importance is not causal proof of an adsorption mechanism.	RQ5	Predictive transferability; Interpretive discipline
100	A3	Yadav S.; Rajput P.; Balasubramanian P.; Liu C.; Li F.; Zhang P. (2025). Machine learning-driven prediction of biochar adsorption capacity for effective removal of Congo red dye. Carbon Research. DOI: 10.1007/s44246-024-00168-3	Multiple / dataset-dependent	Multiple biomass-derived adsorbents / dataset-dependent	Include — core	Predictive / ML evidence	Not necessarily; literature /dataset-based predictive evidence	Yes	Algorithms: RF; XGBoost; GBDT/GBR; SVM/SVR; DT XAI: SHAP; PDP; feature importance Descriptors: textural; surface/chemical; operational; preparation	Not applicable / no DFT model quality audit for predictive-only record	Validation: 10-fold CV; K-fold CV; train/test split; XAI: SHAP; PDP; feature importance; descriptors: textural; surface/chemical; operational; preparation	Not normalized / predictive study	Predictive / descriptor-level evidence	Moderate-to-high: internal validation only	Related Balasubramanian/Liu modeling series; dataset is literature-derived Congo red and should not be assumed independent of other dye compilations.; XAI/feature importance is not causal proof of an adsorption mechanism.; High reported R^2 should not be equated with transferability to unseen adsorbents or pollutants. (see row 114)	Related Balasubramanian/Liu modeling series; dataset is literature-derived Congo red and should not be assumed independent of other dye compilations.; XAI/feature importance is not causal proof of an adsorption mechanism.; High reported R^2 should not be equated with transferability to unseen adsorbents or pollutants. (see row 114)	RQ4, RQ5	Predictive transferability; Interpretive discipline
101	A3	Wang L.; Tang R.; Wu Z.; Gu Y.; Peng X.; Zhang P.; Tan H. (2025). Magnetic iron-crosslinked sodium alginate modified sugarcane bagasse biochar for rapid and efficient removal of tetracycline hydrochloride in water. International Journal of Biological Macromolecules.	Not explicitly normalized in predictive audit	Multiple biomass-derived adsorbents / dataset-dependent	Include — core	Predictive / ML evidence	Not necessarily; literature /dataset-based predictive evidence	Yes	Algorithms: XGBoost; GBDT/GBR; CatBoost XAI: SHAP; PDP; feature importance Descriptors: textural; surface/chemical; operational; preparation	Not applicable / no DFT model quality audit for predictive-only record	Validation: Validation details require targeted extraction; XAI: SHAP; PDP; feature importance	Not normalized / predictive study	Predictive / descriptor-level evidence	Moderate: practical matrix tested, but not necessarily external ML validation	XAI/feature importance is not causal proof of an adsorption mechanism.	XAI/feature importance is not causal proof of an adsorption mechanism.	RQ1, RQ2, RQ4, RQ5	Predictive transferability; Interpretive discipline

		DOI: 10.1016/j.ijbiomac.2025.148099								; descriptors: textural; surface/chemical; operational; preparation							
102	A3	Mahdi Z.; Hanandeh A.E.; Pratt C. (2025). Nonlinear modeling and machine learning techniques are needed for accurate prediction of contaminant sorption. International Journal of Environmental Science and Technology. DOI: 10.1007/s13762-024-06280-6	Not explicitly normalized in predictive audit	Not explicitly normalized in predictive audit	Include — core	Predictive / ML evidence	Not necessarily; literature/dataset-based predictive evidence	Yes	Algorithms: DT XAI: feature importance Descriptors: textural; surface/chemical; molecular; operational; preparation	Not applicable / no DFT model quality audit for predictive-only record	Validation: train/test split; XAI: feature importance; descriptors: textural; surface/chemical; molecular; operational; preparation	Not normalized / predictive study	Predictive / descriptor-level evidence	Moderate-to-high: internal validation only	XAI/feature importance is not causal proof of an adsorption mechanism.; High reported R^2 should not be equated with transferability to unseen adsorbents or pollutants.	XAI/feature importance is not causal proof of an adsorption mechanism.; High reported R^2 should not be equated with transferability to unseen adsorbents or pollutants.	RQ5	Predictive transferability; Interpretive discipline
103	A3	Afrah Solih F.; Buthiyappan A.; Hasikin K.; Myo Aung K.; Abdul Raman A.A. (2025). Optimization-driven modelling of hydrochar derived from fruit waste for adsorption performance evaluation using response surface methodology and machine learning. Journal of Industrial and Engineering Chemistry. DOI: 10.1016/j.jiec.2024.06.042	Not explicitly normalized in predictive audit	Not explicitly normalized in predictive audit	Include — supporting	Predictive / ML evidence	Not necessarily; literature/dataset-based predictive evidence	Yes	Algorithms: RF; ANN/BPNN; SVM/SVR; DT XAI: None/limited Descriptors: textural; surface/chemical; operational	Not applicable / no DFT model quality audit for predictive-only record	Validation: train/test split; XAI: None/limited; descriptors: textural; surface/chemical; operational	Not normalized / predictive study	Predictive / descriptor-level evidence	High/unclear: transferability not demonstrated	No special warning beyond standard XAI/transferability caution.	No special warning beyond standard XAI/transferability caution.	RQ4, RQ5	Predictive transferability; Interpretive discipline
104	A3	Lu, HN; Li, P; Huang, YJ; Liu, F; Gao, BS; Yang, J; Liang, YH; Feng, ZJ (2025). Predicting the Cu adsorption performance of original biochar in aquatic environments via interpretable machine learning models. Journal of Water Process Engineering. DOI: 10.1016/j.jwpe.2025.108079	Not explicitly normalized in predictive audit	Multiple biomass-derived adsorbents / dataset-dependent	Include — core	Predictive / ML evidence	Yes / predictive study includes experimental component	Yes	Algorithms: RF; GBDT/GBR; ANN/BPNN; SVM/SVR; KNN; DT XAI: SHAP; PDP; feature importance Descriptors: textural; surface/chemical; molecular; operational; preparation	Not applicable / no DFT model quality audit for predictive-only record	Validation: K-fold CV; experimental validation; train/test split; XAI: SHAP; PDP; feature importance; descriptors: textural; surface/chemical; molecular; operational	Not normalized / predictive study	Predictive / descriptor-level evidence	Moderate-to-high: internal validation only	XAI/feature importance is not causal proof of an adsorption mechanism.; High reported R^2 should not be equated with transferability to unseen adsorbents or pollutants.	XAI/feature importance is not causal proof of an adsorption mechanism.; High reported R^2 should not be equated with transferability to unseen adsorbents or pollutants.	RQ4, RQ5	Predictive transferability; Interpretive discipline; Evidence convergence

											; preparation							
105	A3	Xie G.; Zhu C.; Li C.; Fan Z.; Wang B. (2025). Predicting the adsorption of ammonia nitrogen by biochar in water bodies using machine learning strategies: Model optimization and analysis of key characteristic variables. Environmental Research. DOI: 10.1016/j.envres.2024.120618	Not explicitly normalized in predictive audit	Multiple biomass-derived adsorbents / dataset-dependent	Include — core	Predictive / ML evidence	Not necessarily; literature /dataset-based predictive evidence	Yes	Algorithms: RF; XGBoost; GBDT/GBR; CatBoost; ANN/BPNN; SVM/SVR; KNN; DT XAI: SHAP; PDP; feature importance Descriptors: textural; surface/chemical; molecular; operational; preparation	Not applicable / no DFT model quality audit for predictive-only record	Validation: 5-fold CV; train/test split; XAI: SHAP; PDP; feature importance; descriptors: textural; surface/chemical; molecular; operational; preparation	Not normalized / predictive study	Predictive / descriptor-level evidence	Moderate-to-high: internal validation only	XAI/feature importance is not causal proof of an adsorption mechanism.; High reported R^2 should not be equated with transferability to unseen adsorbents or pollutants.	XAI/feature importance is not causal proof of an adsorption mechanism.; High reported R^2 should not be equated with transferability to unseen adsorbents or pollutants.	RQ5	Predictive transferability; Interpretive discipline
106	A3	He J.; Xiong B.; Ren M.; Dang Z.; Guo C. (2025). Prediction of removal performance of per- and polyfluoroalkyl substances by biochar based on machine learning models. Journal of Environmental Chemical Engineering. DOI: 10.1016/j.jece.2025.119989	Not explicitly normalized in predictive audit	Multiple biomass-derived adsorbents / dataset-dependent	Include — core	Predictive / ML evidence	Yes / predictive study includes experimental component	Yes	Algorithms: RF; XGBoost; CatBoost; LightGBM; DT XAI: feature importance; sensitivity Descriptors: textural; operational; preparation	Not applicable / no DFT model quality audit for predictive-only record	Validation: 5-fold CV; experimental validation; train/test split; XAI: feature importance; sensitivity; descriptors: textural; operational; preparation	Not normalized / predictive study	Predictive / descriptor-level evidence	Moderate: some external/independent validation	XAI/feature importance is not causal proof of an adsorption mechanism.	XAI/feature importance is not causal proof of an adsorption mechanism.	RQ5	Predictive transferability; Interpretive discipline; Evidence convergence
107	A3	Iftikhar S.; Ishtiaq R.; Zahra N.; Ruba F.; Lam S.-M.; Abbas A.; Jaffari Z.H. (2025). Probabilistic prediction of phosphate ion adsorption onto biochar materials using a large dataset and online deployment. Chemosphere. DOI: 10.1016/j.chemosphere.2024.144031	Not explicitly normalized in predictive audit	Multiple biomass-derived adsorbents / dataset-dependent	Include — core	Predictive / ML evidence	Not necessarily; literature /dataset-based predictive evidence	Yes	Algorithms: RF; XGBoost; GBDT/GBR; ANN/BPNN; DT; Transformer; CNN XAI: SHAP; PDP; feature importance Descriptors: textural; surface/chemical; operational; preparation	Not applicable / no DFT model quality audit for predictive-only record	Validation: 5-fold CV; train/test split; XAI: SHAP; PDP; feature importance; descriptors: textural; surface/chemical;	Not normalized / predictive study	Predictive / descriptor-level evidence	Moderate-to-high: internal validation only	XAI/feature importance is not causal proof of an adsorption mechanism.; High reported R^2 should not be equated with transferability to unseen	XAI/feature importance is not causal proof of an adsorption mechanism.; High reported R^2 should not be equated with transferability to unseen adsorbents or pollutants.	RQ4, RQ5	Predictive transferability; Interpretive discipline

											operational; preparation				adsorbents or pollutants.			
108	A3	Zhu T.; Yang H.; Tao C.; Li Y.; Li S.; Chen X. (2025). Revealing aqueous adsorption mechanisms of organic contaminants and optimizing prediction models from the molecular structure scale. Materials Chemistry and Physics. DOI: 10.1016/j.matchemphys.2025.131234	Not explicitly normalized in predictive audit	Not explicitly normalized in predictive audit	Include — supporting	Predictive / ML evidence	Not necessarily; literature /dataset-based predictive evidence	Yes	Algorithms: RF; XGBoost; GBDT/GBR; CatBoost; ANN/BPNN; SVM/SVR; KNN; DT XAI: SHAP; feature importance Descriptors: textural; surface/chemical; molecular; operational	Not applicable / no DFT model quality audit for predictive-only record	Validation: 10-fold CV; external validation; train/test split; XAI: SHAP; feature importance; descriptors: textural; surface/chemical; molecular; operational	Not normalized / predictive study	Predictive / descriptor-level evidence	Moderate: some external/independent validation	XAI/feature importance is not causal proof of an adsorption mechanism.; Includes multiple carbonaceous adsorbent classes; biochar-specific conclusions must be separated.	XAI/feature importance is not causal proof of an adsorption mechanism.; Includes multiple carbonaceous adsorbent classes; biochar-specific conclusions must be separated.	RQ1, RQ2, RQ4, RQ5	Predictive transferability; Interpretive discipline; Evidence convergence
109	A3	Huang W.; Wang L.; Zhu J.; Dong L.; Hu H.; Yao H.; Wang L.; Lin Z. (2024). Application of machine learning in prediction of Pb2+ adsorption of biochar prepared by tube furnace and fluidized bed. Environmental Science and Pollution Research. DOI: 10.1007/s11356-024-32951-5	Not explicitly normalized in predictive audit	Multiple biomass-derived adsorbents / dataset-dependent	Include — core	Predictive / ML evidence	Not necessarily; literature /dataset-based predictive evidence	Yes	Algorithms: RF; XGBoost; GBDT/GBR; ANN/BPNN; SVM/SVR; DT; GPR/Kriging; AdaBoost XAI: SHAP; PDP; feature importance Descriptors: textural; surface/chemical; molecular; operational; preparation	Not applicable / no DFT model quality audit for predictive-only record	Validation: 5-fold CV; XAI: SHAP; PDP; feature importance; descriptors: textural; surface/chemical; molecular; operational; preparation	Not normalized / predictive study	Predictive / descriptor-level evidence	Moderate-to-high: internal validation only	XAI/feature importance is not causal proof of an adsorption mechanism.; High reported R² should not be equated with transferability to unseen adsorbents or pollutants.	XAI/feature importance is not causal proof of an adsorption mechanism.; High reported R² should not be equated with transferability to unseen adsorbents or pollutants.	RQ4, RQ5	Predictive transferability; Interpretive discipline
110	A3	Wang, YK; Wang, C; An, XM; Wang, R; Li, YY; Xu, YY; Cheng, XW (2024). Effectively removal of PPCPs by catalytic activated biochar derived from hazelnut shell: Modeled and predicted by machine learning. Colloids and Surfaces A: Physicochemical and Engineering Aspects. DOI: 10.1016/j.colsurfa.2024.135059	Not explicitly normalized in predictive audit	Multiple biomass-derived adsorbents / dataset-dependent	Include — core	Predictive / ML evidence	Not necessarily; literature /dataset-based predictive evidence	Yes	Algorithms: RF; GBDT/GBR; ANN/BPNN; SVM/SVR; DT XAI: feature importance Descriptors: textural; surface/chemical; molecular; operational; preparation	Not applicable / no DFT model quality audit for predictive-only record	Validation: train/test split; XAI: feature importance; descriptors: textural; surface/chemical; molecular; operational; preparation	Not normalized / predictive study	Predictive / descriptor-level evidence	Moderate: practical matrix tested, but not necessarily external ML validation	XAI/feature importance is not causal proof of an adsorption mechanism.	XAI/feature importance is not causal proof of an adsorption mechanism.	RQ1, RQ2, RQ4, RQ5	Predictive transferability; Interpretive discipline

111	A3	Shen T.; Peng H.; Yuan X.; Liang Y.; Liu S.; Wu Z.; Leng L.; Qin P. (2024). Feature engineering for improved machine-learning-aided studying heavy metal adsorption on biochar. Journal of Hazardous Materials. DOI: 10.1016/j.jhazmat.2024.133442	Multiple / dataset-dependent	Multiple biomass-derived adsorbents / dataset-dependent	Include — core	Predictive / ML evidence	Not necessarily; literature /dataset-based predictive evidence	Yes	Algorithms: RF; GBDT/GBR; ANN/BPNN; SVM/SVR; DT; GPR/Kriging XAI: PDP; feature importance Descriptors: textural; surface/chemical; operational; preparation	Not applicable / no DFT model quality audit for predictive-only record	Validation: train/test split; XAI: PDP; feature importance; descriptors: textural; surface/chemical; operational; preparation	Not normalized / predictive study	Predictive / descriptor-level evidence	Moderate-to-high: internal validation only	XAI/feature importance is not causal proof of an adsorption mechanism.; High reported R^2 should not be equated with transferability to unseen adsorbents or pollutants.	XAI/feature importance is not causal proof of an adsorption mechanism.; High reported R^2 should not be equated with transferability to unseen adsorbents or pollutants.	RQ1, RQ2, RQ4, RQ5	Predictive transferability; Interpretive discipline
112	A3	Jiang, SY; Hou, YL; Man, ZH; Wang, C; Shi, XQ; Shang, JW; Cheng, XW (2024). Guiding experiment with Machine Learning: A case study of biochar adsorption of Ciprofloxacin. Separation and Purification Technology. DOI: 10.1016/j.seppur.2023.126023	Not explicitly normalized in predictive audit	Multiple biomass-derived adsorbents / dataset-dependent	Include — core	Predictive / ML evidence	Not necessarily; literature /dataset-based predictive evidence	Yes	Algorithms: RF; XGBoost; GBDT/GBR; CatBoost; LightGBM; DT XAI: SHAP; PDP Descriptors: textural; surface/chemical; operational; preparation	Not applicable / no DFT model quality audit for predictive-only record	Validation: K-fold CV; train/test split; XAI: SHAP; PDP; descriptors: textural; surface/chemical; operational; preparation	Not normalized / predictive study	Predictive / descriptor-level evidence	Moderate-to-high: internal validation only	XAI/feature importance is not causal proof of an adsorption mechanism.; High reported R^2 should not be equated with transferability to unseen adsorbents or pollutants.	XAI/feature importance is not causal proof of an adsorption mechanism.; High reported R^2 should not be equated with transferability to unseen adsorbents or pollutants.	RQ5	Predictive transferability; Interpretive discipline
113	A3	Duan Q.; Yan P.; Feng Y.; Wan Q.; Zhu X. (2024). Machine learning assisted adsorption performance evaluation of biochar on heavy metal. Frontiers of Environmental Science and Engineering. DOI: 10.1007/s11783-024-1815-4	Multiple / dataset-dependent	Multiple biomass-derived adsorbents / dataset-dependent	Include — core	Predictive / ML evidence	Not necessarily; literature /dataset-based predictive evidence	Yes	Algorithms: ANN/BPNN XAI: None/limited Descriptors: textural; surface/chemical	Not applicable / no DFT model quality audit for predictive-only record	Validation: Validation details require targeted extraction; XAI: None/limited; descriptors: textural; surface/chemical	Not normalized / predictive study	Predictive / descriptor-level evidence	High/unclear: transferability not demonstrated	No special warning beyond standard XAI/transferability caution.	No special warning beyond standard XAI/transferability caution.	RQ4, RQ5	Predictive transferability; Interpretive discipline
114	A3	Liu C.; Balasubramanian P.; Li F.; Huang H. (2024). Machine learning prediction of dye adsorption by hydrochar: Parameter optimization and experimental validation. Journal of Hazardous Materials. DOI: 10.1016/j.jhazmat.2024.135853	Not explicitly normalized in predictive audit	Not explicitly normalized in predictive audit	Include — core	Predictive / ML evidence	Yes / predictive study includes experimental component	Yes	Algorithms: RF; XGBoost; GBDT/GBR; CatBoost; ANN/BPNN; SVM/SVR; KNN; DT XAI: SHAP; PDP; feature importance Descriptors: textural; surface/chemical; molecular; operational; preparation	Not applicable / no DFT model quality audit for predictive-only record	Validation: 5-fold CV; experimental validation; train/test split; XAI: SHAP; PDP; feature importance; descriptors: textural; surface/che	Not normalized / predictive study	Predictive / descriptor-level evidence	Moderate-to-high: internal validation only	Related Balasubramanian/Liu modeling series; treat as independent only after verifying the experimental dataset.; XAI/feature importance is not causal proof of an adsorption	Related Balasubramanian/Liu modeling series; treat as independent only after verifying the experimental dataset.; XAI/feature importance is not causal proof of an adsorption mechanism.;	RQ4, RQ5	Predictive transferability; Interpretive discipline; Evidence convergence

											mical; molecular; operational; preparation				mechanism.; High reported R^2 should not be equated with transferability to unseen adsorbents or pollutants. (see row 100)	High reported R^2 should not be equated with transferability to unseen adsorbents or pollutants. (see row 100)		
115	A3	Vinayagam R.; Nagendran V.; Goveas L.C.; Narasimhan M.K.; Varadavenkatesan T.; Samanth A.; Selvaraj R. (2024). Machine learning, conventional and statistical physics modeling of 2,4-Dichlorophenoxyacetic acid (2,4-D) herbicide removal using biochar prepared from Vateria indica fruit biomass. Chemosphere. DOI: 10.1016/j.chemosphere.2024.141130	Not explicitly normalized in predictive audit	Multiple biomass-derived adsorbents / dataset-dependent	Include — supporting	Predictive / ML evidence	Not necessarily; literature /dataset-based predictive evidence	Yes	Algorithms: ANN/BPNN; SVM/SVR; Stacking XAI: None/limited Descriptors: textural; surface/chemical; molecular; operational	Not applicable / no DFT model quality audit for predictive-only record	Validation: train/test split; XAI: None/limited; descriptors: textural; surface/chemical; molecular; operational	Not normalized / predictive study	Predictive / descriptor-level evidence	High/unclear: transferability not demonstrated	No special warning beyond standard XAI/transferability caution.	No special warning beyond standard XAI/transferability caution.	RQ1, RQ2, RQ4, RQ5	Predictive transferability; Interpretive discipline
116	A3	Lyu H.; Xu Z.; Zhong J.; Gao W.; Liu J.; Duan M. (2024). Machine learning-driven prediction of phosphorus adsorption capacity of biochar: Insights for adsorbent design and process optimization. Journal of Environmental Management. DOI: 10.1016/j.jenvman.2024.122405	Multiple / dataset-dependent	Multiple biomass-derived adsorbents / dataset-dependent	Include — core	Predictive / ML evidence	Not necessarily; literature /dataset-based predictive evidence	Yes	Algorithms: RF; XGBoost; GBDT/GBR; CatBoost; ANN/BPNN; SVM/SVR; DT XAI: PDP; feature importance Descriptors: textural; surface/chemical; operational; preparation	Not applicable / no DFT model quality audit for predictive-only record	Validation: 5-fold CV; train/test split; XAI: PDP; feature importance; descriptors: textural; surface/chemical; operational; preparation	Not normalized / predictive study	Predictive / descriptor-level evidence	Moderate-to-high: internal validation only	XAI/feature importance is not causal proof of an adsorption mechanism.; High reported R^2 should not be equated with transferability to unseen adsorbents or pollutants.	XAI/feature importance is not causal proof of an adsorption mechanism.; High reported R^2 should not be equated with transferability to unseen adsorbents or pollutants.	RQ5	Predictive transferability; Interpretive discipline
117	A3	Chen, L; Hu, J; Wang, H; He, YY; Deng, QY; Wu, FF (2024). Predicting Cd(II) adsorption capacity of biochar materials using typical machine learning models for effective remediation of aquatic environments. Science of the Total Environment. DOI:	Multiple / dataset-dependent	Multiple biomass-derived adsorbents / dataset-dependent	Include — core	Predictive / ML evidence	Not necessarily; literature /dataset-based predictive evidence	Yes	Algorithms: RF; XGBoost; GBDT/GBR; CatBoost; LightGBM; ANN/BPNN; SVM/SVR; KNN XAI: SHAP; PDP; feature importance Descriptors: textural; surface/chemical; molecular; operational; preparation	Not applicable / no DFT model quality audit for predictive-only record	Validation: 5-fold CV; train/test split; XAI: SHAP; PDP; feature importance; descriptors: textural; surface/che	Not normalized / predictive study	Predictive / descriptor-level evidence	Moderate-to-high: internal validation only	XAI/feature importance is not causal proof of an adsorption mechanism.; High reported R^2 should not be equated with transferability to unseen	XAI/feature importance is not causal proof of an adsorption mechanism.; High reported R^2 should not be equated with transferability to unseen	RQ5	Predictive transferability; Interpretive discipline

		10.1016/j.scitotenv.2024.173955									mical; molecular; operational; preparation				adsorbents or pollutants.	adsorbents or pollutants.		
118	A3	Jaffari Z.H.; Abbas A.; Kim C.-M.; Shin J.; Kwak J.; Son C.; Lee Y.-G.; Kim S.; Chon K.; Cho K.H. (2024). Transformer-based deep learning models for adsorption capacity prediction of heavy metal ions toward biochar-based adsorbents. Journal of Hazardous Materials. DOI: 10.1016/j.jhazmat.2023.132773	Multiple / dataset-dependent	Multiple biomass-derived adsorbents / dataset-dependent	Include — core	Predictive / ML evidence	Not necessarily; literature /dataset-based predictive evidence	Yes	Algorithms: RF; ANN/BPNN; DT; Transformer; Stacking XAI: SHAP; PDP; feature importance Descriptors: textural; surface/chemical; molecular; operational; preparation	Not applicable / no DFT model quality audit for predictive-only record	Validation: K-fold CV; train/test split; XAI: SHAP; PDP; feature importance; descriptors: textural; surface/chemical; molecular; operational; preparation	Not normalized / predictive study	Predictive / descriptor-level evidence	Moderate: practical matrix tested, but not necessarily external ML validation	Same research family as row 119; check dataset reuse before quantitative synthesis.; XAI/feature importance is not causal proof of an adsorption mechanism.	Same research family as row 119; check dataset reuse before quantitative synthesis.; XAI/feature importance is not causal proof of an adsorption mechanism.	RQ5	Predictive transferability; Interpretive discipline
119	A3	Haider Jaffari Z.; Jeong H.; Shin J.; Kwak J.; Son C.; Lee Y.-G.; Kim S.; Chon K.; Hwa Cho K. (2023). Machine-learning-based prediction and optimization of emerging contaminants' adsorption capacity on biochar materials. Chemical Engineering Journal. DOI: 10.1016/j.cej.2023.143073	Multiple / dataset-dependent	Multiple biomass-derived adsorbents / dataset-dependent	Include — core	Predictive / ML evidence	Not necessarily; literature /dataset-based predictive evidence	Yes	Algorithms: RF; XGBoost; GBDT/GBR; CatBoost; ANN/BPNN; SVM/SVR; KNN; DT XAI: SHAP; PDP; feature importance Descriptors: textural; surface/chemical; molecular; operational; preparation	Not applicable / no DFT model quality audit for predictive-only record	Validation: train/test split; XAI: SHAP; PDP; feature importance; descriptors: textural; surface/chemical; molecular; operational; preparation	Not normalized / predictive study	Predictive / descriptor-level evidence	Moderate: practical matrix tested, but not necessarily external ML validation	Same research family as row 118; check dataset reuse before quantitative synthesis.; XAI/feature importance is not causal proof of an adsorption mechanism.	Same research family as row 118; check dataset reuse before quantitative synthesis.; XAI/feature importance is not causal proof of an adsorption mechanism.	RQ4, RQ5	Predictive transferability; Interpretive discipline
120	A3	Nighojkar A.; Nagpal J.; Soboyejo W.; Plappally A.; Pandey S. (2023). Prediction of organophosphorus pesticide adsorption by biochar using ensemble learning algorithms. Environmental Monitoring and Assessment. DOI: 10.1007/s10661-023-11599-7	Not explicitly normalized in predictive audit	Multiple biomass-derived adsorbents / dataset-dependent	Include — core	Predictive / ML evidence	Yes / predictive study includes experimental component	Yes	Algorithms: RF; XGBoost; GBDT/GBR; SVM/SVR; KNN; DT; Transformer XAI: SHAP; PDP; feature importance Descriptors: textural; surface/chemical; operational; preparation	Not applicable / no DFT model quality audit for predictive-only record	Validation: 5-fold CV; external validation; experimental validation; train/test split; XAI: SHAP; PDP; feature importance	Not normalized / predictive study	Predictive / descriptor-level evidence	Moderate: some external/independent validation	XAI/feature importance is not causal proof of an adsorption mechanism.	XAI/feature importance is not causal proof of an adsorption mechanism.	RQ4, RQ5	Predictive transferability; Interpretive discipline; Evidence convergence

											; descriptors: textural; surface/chemical; operational; preparation							
121	A3	Liu, JX; Xu, ZL; Zhang, WJ (2023). Unraveling the role of Fe in As(III & V) removal by biochar via machine learning exploration. SEPARATION AND PURIFICATION TECHNOLOGY. DOI: 10.1016/j.seppur.2023.123245	Not explicitly normalized in predictive audit	Multiple biomass-derived adsorbents / dataset-dependent	Include — core	Predictive / ML evidence	Not necessarily; literature /dataset-based predictive evidence	Yes	Algorithms: RF; DT XAI: PDP; feature importance Descriptors: textural; surface/chemical; operational; preparation	Not applicable / no DFT model quality audit for predictive-only record	Validation: train/test split; XAI: PDP; feature importance; descriptors: textural; surface/chemical; operational; preparation	Not normalized / predictive study	Predictive / descriptor-level evidence	Moderate: practical matrix tested, but not necessarily external ML validation	XAI/feature importance is not causal proof of an adsorption mechanism.	XAI/feature importance is not causal proof of an adsorption mechanism.	RQ4, RQ5	Predictive transferability; Interpretive discipline
122	A3	Zhu, XZ; He, MJ; Sun, YQ; Xu, ZB; Wan, ZH; Hou, DY; Alessi, DS; Tsang, DCW (2022). Insights into the adsorption of pharmaceuticals and personal care products (PPCPs) on biochar and activated carbon with the aid of machine learning. Journal of Hazardous Materials. DOI: 10.1016/j.jhazmat.2021.127060	Not explicitly normalized in predictive audit	Multiple biomass-derived adsorbents / dataset-dependent	Include — core	Predictive / ML evidence	Not necessarily; literature /dataset-based predictive evidence	Yes	Algorithms: RF; GBDT/GBR; DT XAI: PDP; feature importance Descriptors: textural; surface/chemical; molecular; operational; preparation	Not applicable / no DFT model quality audit for predictive-only record	Validation: 5-fold CV; external validation; XAI: PDP; feature importance; descriptors: textural; surface/chemical; molecular; operational; preparation	Not normalized / predictive study	Predictive / descriptor-level evidence	Moderate: some external/independent validation	Related research family/data domain to row 124; check overlap of carbon-material/PPCP datasets.; XAI/feature importance is not causal proof of an adsorption mechanism.	Related research family/data domain to row 124; check overlap of carbon-material/PPCP datasets.; XAI/feature importance is not causal proof of an adsorption mechanism.	RQ4, RQ5	Predictive transferability; Interpretive discipline; Evidence convergence
123	A3	Nguyen X.C.; Ly Q.V.; Nguyen T.T.H.; Ngo H.T.T.; Hu Y.; Zhang Z. (2022). Potential application of machine learning for exploring adsorption mechanisms of pharmaceuticals onto biochars. Chemosphere. DOI: 10.1016/j.chemosphere.2021.132203	Not explicitly normalized in predictive audit	Multiple biomass-derived adsorbents / dataset-dependent	Include — core	Predictive / ML evidence	Not necessarily; literature /dataset-based predictive evidence	Yes	Algorithms: RF; XGBoost; GBDT/GBR; ANN/BPNN; SVM/SVR; KNN; DT XAI: feature importance; sensitivity Descriptors: textural; surface/chemical; molecular; operational; preparation	Not applicable / no DFT model quality audit for predictive-only record	Validation: 10-fold CV; train/test split; XAI: feature importance; sensitivity; descriptors: textural; surface/chemical; molecular; operational;	Not normalized / predictive study	Predictive / descriptor-level evidence	Moderate: practical matrix tested, but not necessarily external ML validation	XAI/feature importance is not causal proof of an adsorption mechanism.	XAI/feature importance is not causal proof of an adsorption mechanism.	RQ4, RQ5	Predictive transferability; Interpretive discipline

											preparation							
124	A3	Zhu, XZ; Wan, ZH; Tsang, DCW; He, MJ; Hou, DY; Su, ZS; Shang, J (2021). Machine learning for the selection of carbon-based materials for tetracycline and sulfamethoxazole adsorption. Chemical Engineering Journal. DOI: 10.1016/j.cej.2020.126782	Not explicitly normalized in predictive audit	Not explicitly normalized in predictive audit	Include — supporting	Predictive / ML evidence	Not necessarily; literature /dataset-based predictive evidence	Yes	Algorithms: RF; GBDT/GBR; ANN/BPNN; DT; Stacking XAI: PDP; feature importance Descriptors: textural; surface/chemical; molecular; operational; preparation	Not applicable / no DFT model quality audit for predictive-only record	Validation: 5-fold CV; external validation; train/test split; XAI: PDP; feature importance; descriptors: textural; surface/chemical; molecular; operational; preparation	Not normalized / predictive study	Predictive / descriptor-level evidence	Moderate: some external/independent validation	Related research family/data domain to row 122; check overlap of carbon-material/PPCP datasets.; XAI/feature importance is not causal proof of an adsorption mechanism.	Related research family/data domain to row 122; check overlap of carbon-material/PPCP datasets.; XAI/feature importance is not causal proof of an adsorption mechanism.	RQ4, RQ5	Predictive transferability; Interpretive discipline; Evidence convergence
125	A3	Zhu X.; Wang X.; Ok Y.S. (2019). The application of machine learning methods for prediction of metal sorption onto biochars. Journal of Hazardous Materials. DOI: 10.1016/j.jhazmat.2019.06.004	Not explicitly normalized in predictive audit	Multiple biomass-derived adsorbents / dataset-dependent	Include — core	Predictive / ML evidence	Not necessarily; literature /dataset-based predictive evidence	Yes	Algorithms: RF; ANN/BPNN; SVM/SVR; DT XAI: feature importance Descriptors: textural; surface/chemical; molecular; operational; preparation	Not applicable / no DFT model quality audit for predictive-only record	Validation: 10-fold CV; train/test split; XAI: feature importance; descriptors: textural; surface/chemical; molecular; operational; preparation	Not normalized / predictive study	Predictive / descriptor-level evidence	Moderate: practical matrix tested, but not necessarily external ML validation	XAI/feature importance is not causal proof of an adsorption mechanism.; Foundational ML study; interpretability methods are older and less granular than current XAI workflows.	XAI/feature importance is not causal proof of an adsorption mechanism.; Foundational ML study; interpretability methods are older and less granular than current XAI workflows.	RQ4, RQ5	Predictive transferability; Interpretive discipline

Note on the RQ2 columns. "RQ2 manual-audit status" reports the outcome of the claim-level overinterpretation audit described in Section 9.3 and Supplementary Table S6. Candidate claims were identified through a rule-based screen and the evidence-charting notes and were verified at full text. "Confirmed overinterpretation" denotes a mechanistic inference from kinetic or equilibrium-model fitting confirmed at full text, irrespective of whether independent evidence also supported a similar mechanistic conclusion; "False positive" denotes a candidate that did not meet the criterion; and "Unresolved" denotes a candidate whose full text could not be verified, which is excluded from the confirmed counts. "No RQ2 flag identified" indicates that no candidate claim was identified, not a demonstrated absence of such an inference. A confirmed flag identifies a specific unsupported inferential step and does not imply that the overall mechanistic conclusion of the study is incorrect. For predictive studies, the "Experiment–theory convergence (mechanistic) / transferability risk (predictive)" column reports the risk that predictive transferability is overstated, from Low (generalization explicitly tested beyond the training domain) to High (transferability not demonstrated). This rating reflects how rigorously transferability was tested, not the predictive performance achieved outside the training domain; rigorous grouped or external validation may reveal a substantial loss of performance (e.g., row 83). Final corpus: 70 mechanistic + 55 predictive studies. Cells marked 'not explicitly normalized/charted' were deliberately left conservative rather than inferred beyond the audited workbooks.

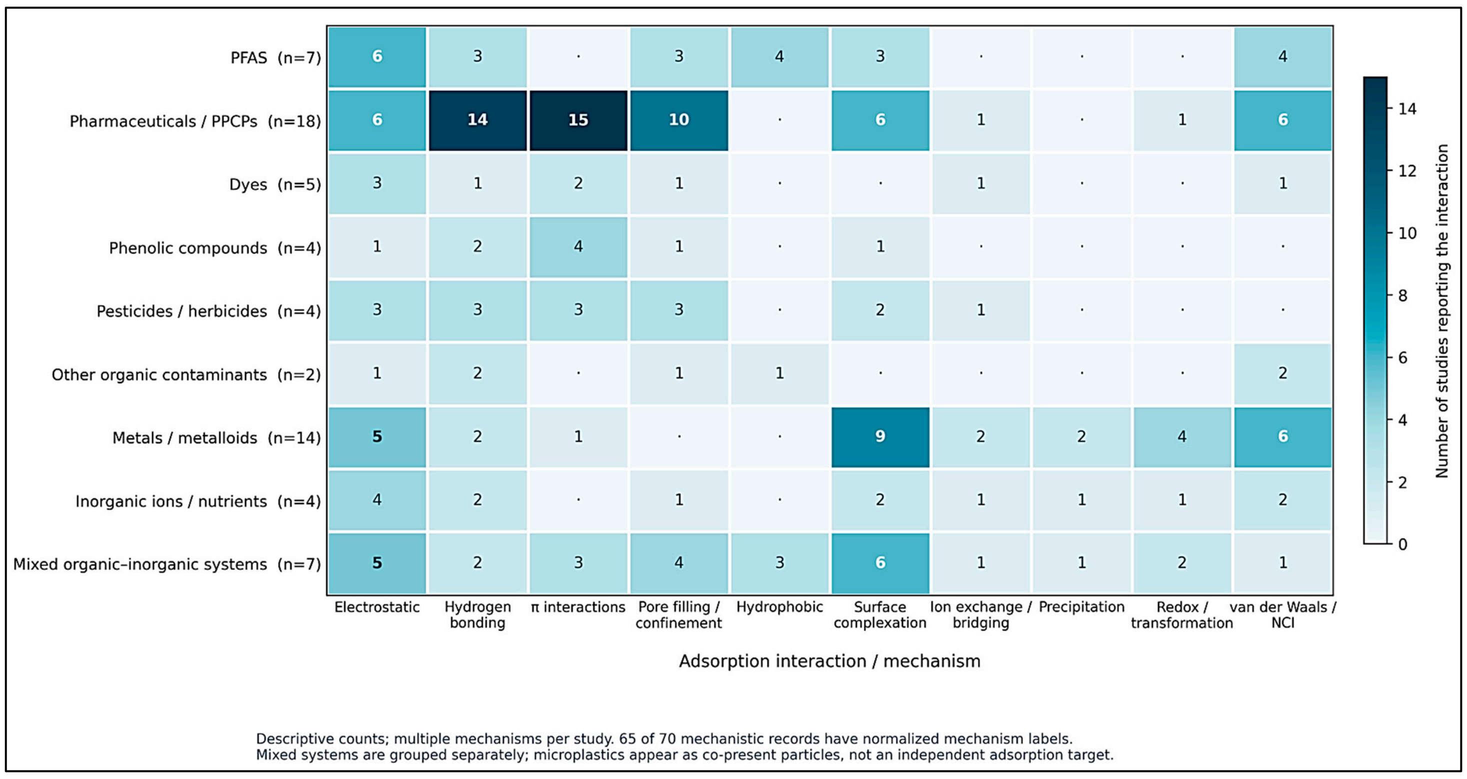

Figure S1. Heat map of reported adsorption interactions across pollutant classes in the mechanistic evidence corpus. Cell values indicate the number of studies reporting each pollutant–mechanism association. The analysis includes 65 of the 70 retained mechanistic studies; five records were excluded from the heat-map counts because their mechanism labels were not explicitly normalized in the source-level evidence charting. Pollutant classes were assigned to mutually exclusive categories for visualization, with mixed organic–inorganic systems represented separately. Multiple adsorption mechanisms could be assigned to a single study. Therefore, the frequencies describe reported associations rather than interaction strengths, mechanistic dominance, or independent experimental confirmation. Microplastics were identified as co-present particulate materials in a multicomponent system rather than as a separate adsorption-target category.
